# HPV16 integration probably contributes to cervical oncogenesis through interrupting tumor suppressor genes and inducing chromosome instability

**DOI:** 10.1186/s13046-016-0454-4

**Published:** 2016-11-25

**Authors:** Jun-Wei Zhao, Fang Fang, Yi Guo, Tai-Lin Zhu, Yun-Yun Yu, Fan-Fei Kong, Ling-Fei Han, Dong-Sheng Chen, Fang Li

**Affiliations:** 1 Department of Gynecology, Shanghai First Maternity and Infant Hospital, Tongji University School of Medicine, Shanghai, 200040 China; 2 Abbey College Cambridge, Homerton Gardens, Cambridge, CB2 8EB UK; 3 Department of Genetics, University of Cambridge, Cambridge, CB2 3EH UK; 4 Fitzwilliam College, University of Cambridge, Storey’s Way, Cambridge, CB3 0DG UK

**Keywords:** HPV16, Integration, Cervical oncogenesis, Chromosome instability

## Abstract

**Background:**

The integration of human papilloma virus (HPV) into host genome is one of the critical steps that lead to the progression of precancerous lesion into cancer. However, the mechanisms and consequences of such integration events are poorly understood. This study aims to explore those questions by studying high risk HPV16 integration in women with cervical intraepithelial neoplasia (CIN) and cervical squamous cell carcinoma (SCC).

**Methods:**

Specifically, HPV integration status of 13 HPV16-infected patients were investigated by ligation-mediated PCR (DIPS-PCR) followed by DNA sequencing.

**Results:**

In total, 8 HPV16 integration sites were identified inside or around genes associated with cancer development. In particular, the well-studied tumor suppressor genes *SCAI* was found to be integrated by HPV16, which would likely disrupt its expression and therefore facilitate the migration of tumor. On top of that, we observed several cases of chromosome translocation events coincide with HPV integration, which suggests the existence of chromosome instability. Additionally, short overlapping sequences were observed between viral derived and host derived fragments in viral-cellular junctions, indicating that integration was mediated by micro homology-mediated DNA repair pathway.

**Conclusions:**

Overall, our study suggests a model in which HPV16 might contribute to oncogenesis not only by disrupting tumor suppressor genes, but also by inducing chromosome instability.

**Electronic supplementary material:**

The online version of this article (doi:10.1186/s13046-016-0454-4) contains supplementary material, which is available to authorized users.

## Background

Cervical cancer is one of the most common types of cancer in women around the world. HPV types 16 and 18 are predominant genotypes leading to cervical cancer, while other genotypes such as HPV 31, 45 and 58 are mainly associated with intra-epithelial and cancerous lesions [1, 2]. Although HPV16 and 18 are considered to be the most prevalent HPV types contributing to cervical cancers worldwide, HPV18 is not the most common genotype in Asian countries and our previous results suggested that HPV16 and 52, instead of HPV18, are the top two prevalent types in Shanghai women [3–6]. The viral DNA is usually found to integrate into the host genome with subsequent disruption of one or more viral open reading frames (ORFs) [7]. HPV integration frequently disrupts in the E1 and/or E2 ORFs. Luft, F. et al. reported that the viral E1 open reading frame (ORF) was fused to cellular sequences in 20 of 22 cases [8]. Interestingly, recent study suggests that HPV16 could establish latent infection in morphologically normal women [9]. By now, most of the research about HPV integration focused on the population of cervical cancer patients, which left the integration of viral fragments in CIN or SCC patients largely unexplored. Besides, the distribution characteristics about the HPV integration sites in the host genomes are not fully understood, and the study on mechanisms by which HPV integrated into human genes is still in its infancy. In this study, we focus on integration sites analysis of HPV16, the most common types of HPV in Shanghai, trying to understand the HPV16 integration characteristics and the potential consequence of viral fragment integration.

In a systematic analysis on more than 1500 integration sites of HPV collected from literature, Bodelon and colleagues found integration events were enriched in 10 cytobands (3q28, 8q24.21 and 13q22.1,2q22.3, 3p14.2, 8q24.22, 14q24.1, 17p11.1, 17q23.1 and 17q23.2). Besides, they noticed that there was significantly higher chance for HPV18 to be integrated in 8q24.21 than HPV16 in Cervical infections (*p* = 6.93e-9). Based on the observation that integration sites were closely linked to transcriptionally active regions, fragile sites, CpG regions, and enhancers, they proposed that HPV tend to integrate in open chromatin regions, which might affect the transcription of corresponding genes. Another interesting finding in their study was that HPV integration events rarely occurred in the vicinity of known cervical cancer driver genes (within 50 Kb) [10].

## Methods

### Clinical samples collection

Cast-off cells of end cervical tissues were obtained from patients using cell brush in Cervical Disease Centre of Shanghai First Maternity and Infant Health Hospital, TongJi University School of Medicine (Shanghai, China).

### Genomic DNA extraction

Genomic DNA from cervical cell brush samples was purified by silica gel columns (TIANamp Genomic DNA Kit No: 3304–9; TIANGEN Biotech Corporation, China) according to the manufacturer’s procedure. Final elution of DNA was resuspended in 50 μl of distilled water. And the DNA samples with concentration greater than 80 ng/μl were used to detect the HPV16 integration sites by DIPS-PCR.

### DIPS-PCR

The DIPS-PCR was performed following the protocol in Luft F et al’s paper [8].

### Sequencing

PCR products were excised from an agarose gel, purified. Subsequently, direct sequencing was performed by SAIYIN gene biotechnology company (SaiYin gene biotechnology company, Shanghai, China).

### Mapping of viral and cellular sequences in viral-cellular junctions

The whole junction sequences were blast against NCBI HPV16 (taxid: 333760) database, the option ‘Somewhat similar sequences (blastn)’ was used. The part of sequences with alignment with HPV sequences were annotated as viral sequences. Subsequently, the whole junction sequences were blasted against Ensemble database (GRCh38) using the blastn tool and with search sensitivity ‘distant homologies’. The aligned sequences with human reference genome were annotated as cellular sequence.

### Extract of gene lists around HPV integration sites

The locations of cellular fragments were obtained by manually checking the blast result. In Ensemble Biomart [11], all the protein encoding genes within certain distances (1 mega bases or 10 mega bases) from HPV16 integration center were downloaded for further analysis.

### GO enrichment analysis of gene lists

GO enrichment was performed using Gorilla [12], with the option ‘two unranked lists of genes (target and background lists)’. The background gene list (all protein coding genes in human) was downloaded from Ensemble database. FDR method was used to calculate the adjusted *P* value.

### Protein interaction network analysis

Those genes directly overlapped with HPV integrating sites were searched in STRING database [13] one by one. The protein interaction networks acquired from STRING were downloaded for further analysis.

### Hi-C data analysis

The chromosome interaction data of GM12878 was downloaded from the Interactive Hi-C Data Browser [14].

## Results

### Overview of viral-cellular junctions in 13 patients infected with HPV16

In this study, 13 DNA samples from HPV16 positive patients with different grades of cervical neoplasia and cervical cancer were analyzed using ligation-mediated PCR (DIPS-PCR) combined with Sanger sequencing (Table 1). In total, 6 out of 13 samples were found to be integrated by HPV16 in cellular genome. Specifically, two integration sites were identified in samples S2-25 and C5-87 respectively. One integration site was found in each of the remaining 4 samples (S1-2, C3-64, C4-77 and S6-95).

**Table 1:** The samples collected from HPV positive patients

Sample name	Age (years)	Pathology	Number of validated viral-cellular junctions
S1-2	41	SCC	1
S2-25	45	HSIL	2
C3-64	45	HSIL	1
C4-77	32	HSIL	1
C5-87	49	HSIL	2
S6-95	48	SCC	1
C7-35	44	Normal	0
C8-7	51	HSIL	0
S9-10	38	SCC	0
S10-11	43	SCC	0
C11-60	28	HSIL	0
C12-67	42	SCC	0
C13-75	35	LSIL	0

HSIL: high-grade squamous intraepithelial lesion, LSIL: low grade squamous intraepithelial lesion, SCC: squamous cell carcinoma. The sample ID, age of pateints, Pathology of patients and number of validated viral-cellular junctions in each sample were shown in column 1, 2, 3 and 4 respectively

### Architectures of 8 viral-cellular junctions

In total, there are 8 viral-cellular junctions were identified from those 6 samples (Table 2 and Additional file 1: File S1). For the sake of clarity, each junction was assigned an unique ID such as ‘S1-2:J-01’ which begins with sample number and follows by junction number. Those 8 junctions can be divided into type 1 junctions (S2-25:J-03, S2-25:J-04 and C5-87:J-08) and type 2 junctions (S1-2:J-01, C3-64:J-05, C4-77:J-06, C5-87:J-07 and S6-95:J-09). Type 1 junctions are 3-element chimera with the architecture of virus-human-human or human-human-virus, while type 2 junctions are 2-element chimera with the architecture of virus-human or human-virus (Table 2).

**Table 2:** Overview of architectures of 8 viral-cellular junctions

Junction ID	Type	Left element location	Middle element location	Right element location	Overlapping
S1-2:J-01	Type 2	HPV16 *E1*(211 bp)	NA	chr3:175701251-175701313 (63 bp) *NAALADL2*	Left & Right: No overlapping
S2-25:J-03	Type 1	HPV16 *E1*(122 bp)	chr9:125080078-125080338(261 bp) *SCAI*	chr4:148216738-148216850 (159 bp) *NR3C2*	Left & Middle: GATGCA Middle & Right: GATC
S2-25:J-04	Type 1	HPV16 *E1*(185 bp)	chr17:30708288-30708380(93 bp)	chr1:165472779-165473100(322 bp)	Left &Middle: No overlapping Middle & Right: AGATC
C3-64:J-05	Type 2	HPV16 *E2*(325 bp)	NA	chr3:124457727-124457915 (189 bp) *KALRN*	Left & Right: AA
C4-77:J-06	Type 2	chr4:185352615-185352687 *SNX25*(72 bp)	NA	HPV16 *L1*(310 bp)	Left & Right: No overlapping
C5-87:J-07	Type 2	chr22:34824077-34824170(93 bp)	NA	HPV16 *L1*(224 bp)	Left & Right: CAATA
C5-87:J-08	Type 1	chr22:34819634-3481983 (201 bp)	chrX:109333446-109333511(66 bp)	HPV16 *E5* (299 bp)	Left & Middle: GTGG Middle & Right: GTGTT
S6-95:J-09	Type 2	HPV16 *E1*(239 bp)	NA	chr16:82782336-82782494 (159 bp) *CDH13*	Left & Right: CTGCAA

Each viral-cellular junction was assigned an unique ID. 8 junctions were divided into 2 types according to the architecture of viral-cellular junctions. Type 1: 3-element junction with the architecture of virus-human-human or human-human-virus. Type 2: 2-element junction with the architecture of virus-human or human-virus. The origin of each elements were shown in column 3, 4 and 5 respectively. The overlapping between every two elements were shown in column 6. NA, not available

### Cellular genes directly overlapping with viral-cellular junctions

S1-2:J-01 belongs to type 2 junction (virus-human), with a 211 base pairs (bp) left element from HPV16 *E1* gene and a 63 bp right element from chromosome 3 located in the intron of *N-acetyl-l-aspartyl-l-glutamate peptidase-like 2 (NAALADL2*). S2-25:J-03 belongs to type 1 junction (virus-human-human), with the left element from HPV16 *E1* gene (122 bp), middle element from chromosome 9 (261 bp) overlapped with *suppressor of cancer cell invasion* (*SCAI*) gene and the right element from chromosome 4 (159 bp) overlapping with *Nuclear Receptor Subfamily 3, Group C, Member 2* (*NR3C2*) gene. Six nt (GATGCA) were overlapped between left viral element and middle cellular element while four nt (GATC) were found to be overlapped between middle and right cellular elements.S2-25:J-04 is a type 1 junction (virus-human-human), composed of HPV16 *E1* gene (185 bp) flowed by chromosome 17 (93 bp) and chromosome 1 (322 bp). No overlapping cellular gene was found in this junction.C3-64:J-05 is a type 2 junction (human-virus), with HPV16 *E2* gene (325 bp) in the left, chromosome 3 (189 bp) in the right, located in the intron of *Kalirin, RhoGEF Kinase* (*KALRN*). Two nt (AA) were found in those two elements.C4-77:J-06 belongs to type 2 junction (human-virus) with the left element from chromosome 4 (72 bp) overlapping with *SNX25* and right element from HPV16 *L1* gene (310 bp). No overlapping was found in this junction. C5-87: J-07 belongs to type 2 junction (human-virus) composed of chromosome 22 (93 bp) and HPV16 *L1* gene (224 bp) with left and right elements overlap by 5 nt (CAATA). C5-87: J-08 is a type 1 junction (human-human-virus) with chromosome 22 (201 bp) in the left, followed by chromosome X (66 bp) and HPV16 E5&L2 gene (299 bp). The left and middle element overlap by four nt (GTGG) while middle and right element overlap by five nt (GTGTT). S6-95: J-09 is a type 2 junction (virus-human) composed of HPV16 E1 gene (239 bp) and chromosome 16 (159 bp) which directly overlap with the intron of *Cadherin 13* (*CDH13*). Six nt (CTGCAA) were found to be overlapped between left viral and right cellular fragments.

### Cellular genes located around HPV16 integration sites

Among the 8 integration events, 5 are directly overlapped with the intron region of protein encoding genes (*NAALADL2*, *SCAI*, *KALRN*, *SNX25*, *CDH13*). Others are located in intergenic regions of protein coding genes. To investigate those genes around integrating sites, we extracted all genes located within 1 mega bases and 10 mega bases from the integrating site center. They were called G1 and G10 data sets, and 58 and 758 protein encoding genes were found in those two data sets respectively (Additional file 1: Tables S1 and S2). After that, we performed Gene Ontology (GO) enrichment analysis to reveal the potential biological process affected by HPV16 integration. Our result suggested that there were no enriched GO terms in G1 data set while 5 GO terms were found to be enriched with adjusted p value (FDR correction) smaller than 0.05 in G10 data set (Table 3). Interestingly, they are related to the transfer of genetic materials (GO:0010528 regulation of transposition), the migration of tumor (GO:0002548 monocyte chemotaxis), immune response (GO:0071346 cellular response to interferon-gamma and GO:0034341 response to interferon-gamma) and DNA repair (GO:0070383 DNA cytosine deamination).

**Table 3:** Enriched GO terms in G10 data sets (758 genes which are located with 10 mega from the HPV16 integration sites)

GOterm	Description	*P*-value	FDR q-value
GO:0010528	Regulation of transposition	1.37E-07	1.92E-03
GO:0002548	Monocyte chemotaxis	6.18E-07	2.88E-03
GO:0071346	Cellular response to interferon-gamma	1.47E-06	5.14E-03
GO:0034341	Response to interferon-gamma	4.00E-06	1.12E-02
GO:0070383	DNA cytosine deamination	1.47E-05	3.44E-02

The ID, description, *P*-value and FDR q-value (adjusted P value) were shown in column 1, 2, 3 and 4 respectively

### Common fragile sites located around HPV16 integrating sites

Chromosomal fragile sites are unstable chromosome regions that are prone to breakage under replication stress [15]. Fragile sites can be classified into ‘rare’ and ‘common’ ones based on the frequencies of those sites appearing in population. Common fragile sites (CFSs) are those present in almost all individuals. By now, more than 120 CFSs have been identified from human chromosomes [15, 16]. We compared the 8 HPV16 integration sites with those known CFSs and found that 11 CFSs have been to found to be located around HPV16 integration sites (Table 4).

**Table 4:** The CFSs located around HPV16 integration sites

Junction ID	Integrating location	Nearest CFSs
S1-2:J-01	3q26.31	FRA3C(3q27)
S2-25:J-03	9q33.3 and 4q31.22	FRA9E(9q32) and FRA4C(4q31.1)
S2-25:J-04	17q11.2 and 1q23.3	FRA17A(17q23) and FRA1G(1q25)
C3-64:J-05	3q21.2	FRA3F(3q22)
C4-77:J-06	4q35.1	FRA4C(4q31.1)
C5-87:J-07	22q12.3	FRA22B(22q12.2)
C5-87:J-08	22q12.3 and Xq23	FRA22B(22q12.2) and FRAXD(Xq27)
S6-95:J-09	16q23.3	FRA16D(16q23)

The junction ID, integrating location in the chromosome (shown as cytobands), nearest common fragile sites (CFSs) to the integration sites were shown in column 1, 2 and 3 respectively

### Chromosome translocation around HPV16 integration sites

**Fig. 1:**
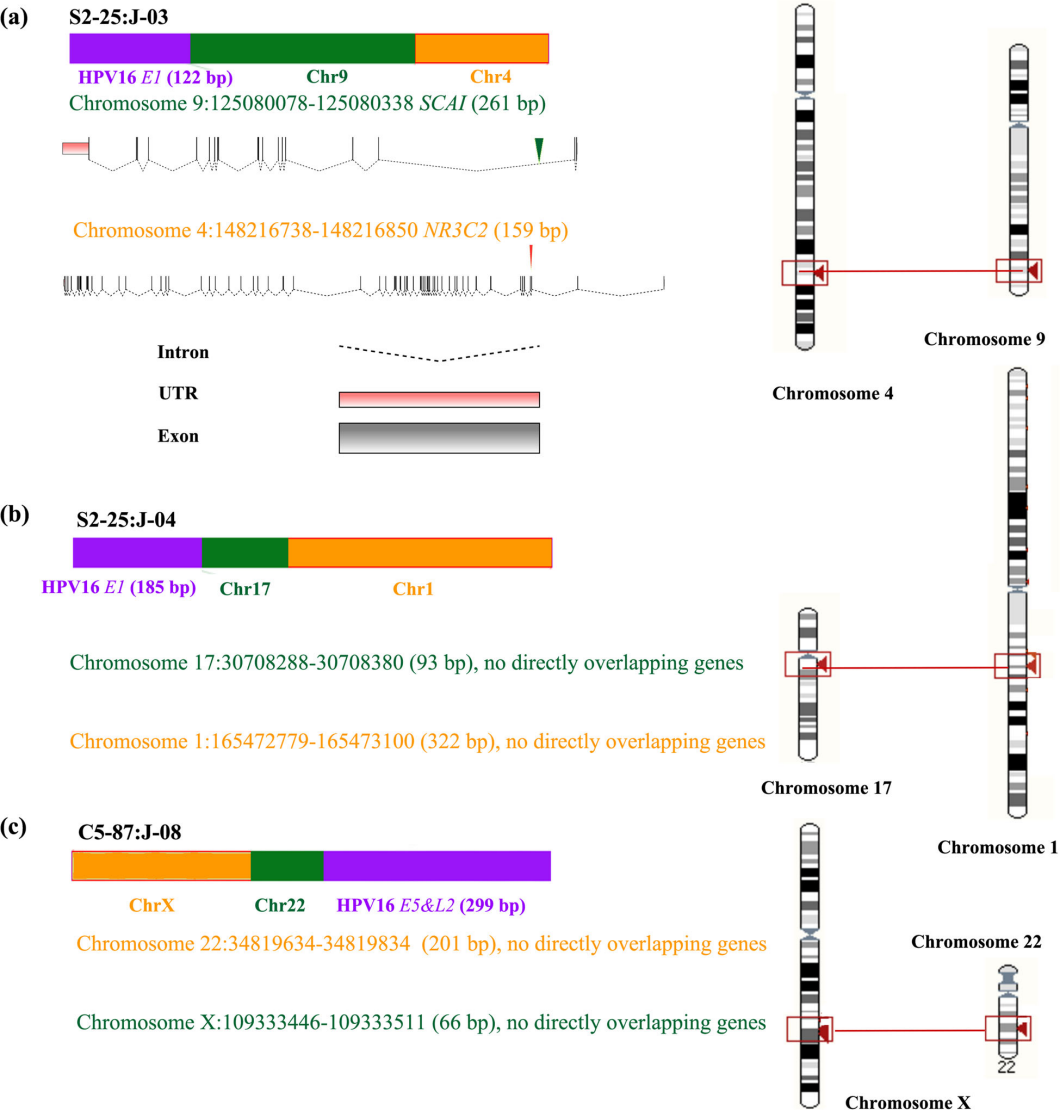
Chromosome translocation events identified by sequencing in this study. The ideograms for the architectures of viral-cellular junctions (*left*) and the location of fusion DNA on chromosomes (*right*) were shown for junctions S2-25:J-03 (**a**), S2-25:J-04 (**b**) and C5-87:J-08 (**c**) respectively. The HPV16 viral fragment was shown in *purple rectangle*, cellular fragments were shown in *orange* or *green rectangle*. The gene structure of *SCAI* and *NR3C2* genes were shown in (**a**) below the ideogram of junction S2-25:J-03, the location of human fragment deriving from *SCAI* and *NR3C2* genes were shown as *green* and *red arrow* respectively

Among the 8 integrating sites identified in this study, 3 of them are found to coincide with chromosome translocation. In the case of S2-25: J-03, we observed the translocation between chromosome 9 and chromosome 4. In S2-25: J-04, the translocation happened between chromosome 17 and chromosome 1. In C5-87: J-08, we identified another translocation event which happened between chromosome 22 and chromosome X (Fig. 1).

### HPV affected cancer-related genes documented in Dr.VIS v2.0 database

In order to address the HPV affected cancer-related genes at a larger scale, we downloaded the annotation of HPV integration events from Dr.VIS v2.0 database. After filtering out records without gene annotation and those missing the information of distance between integration sites and nearest gene, 343 well-annotated records were left for further analysis. By checking the gene list manually, we found several cancer related genes integrated by HPV either in the intron or 5’ flanking region (Table 5), they were *TNFSF4 (tumor necrosis factor superfamily member 4*), *TP63 (tumor protein p63*), *TP73* (*tumor protein p73*), *ARHGEF4* (*Rho guanine nucleotide exchange factor 4*), *CDH13* (*cadherin 13*), *RASSF6* (*Ras association domain family member 6*) [17–30].

**Table 5:** Overview of cancer related genes closely linked to HPV integration sites documented in Dr.VIS v2.0 database [72]

ID	Method	Disease	Subtype	Cytoband	Nearest gene
DRVIS03185	PCR	HNC	HPV16	1q25	*TNFSF4*
DRVIS03195	Exome-Seq	CC	HPV56	3q28	*TP63*
DRVIS03198	Mapping	CC	HPV16	1p36.32	*TP73*
DRVIS02845	RS-PCR	CC	HPV16	4q13.3	*RASSF6*
DRVIS00089	DIPS-PCR	CC	HPV16	2q21.2	*ARHGEF4*
DRVIS00246	DIPS-PCR	CC	HPV16	16q24	*CDH13*

The ID of integration events in Dr.VIS v2.0 database, methods for the detection of viral integration, disease associated with samples, HPV subtype and integration locations were shown in column 1, 2, 3, 4, 5 and 6 respectively. HNC, head and neck cancer; CC, cervical carcinoma; Exome-Seq, Exome sequencing; DIPS-PCR, detection of integrated papillomavirus sequences by ligation-mediated PCR; RS-PCR, RNA Template-Specific PCR; Mapping, mapping analysis. *TNFSF4* (*tumor necrosis factor superfamily member 4*), *TP63* (*tumor protein p63*), *TP73* (*tumor protein p73*), *ARHGEF4* (*Rho guanine nucleotide exchange factor 4*), *CDH13* (*cadherin 13*), *RASSF6* (*Ras association domain family member 6*)

### Variability of inserted HPV fragments

**Fig. 2:**
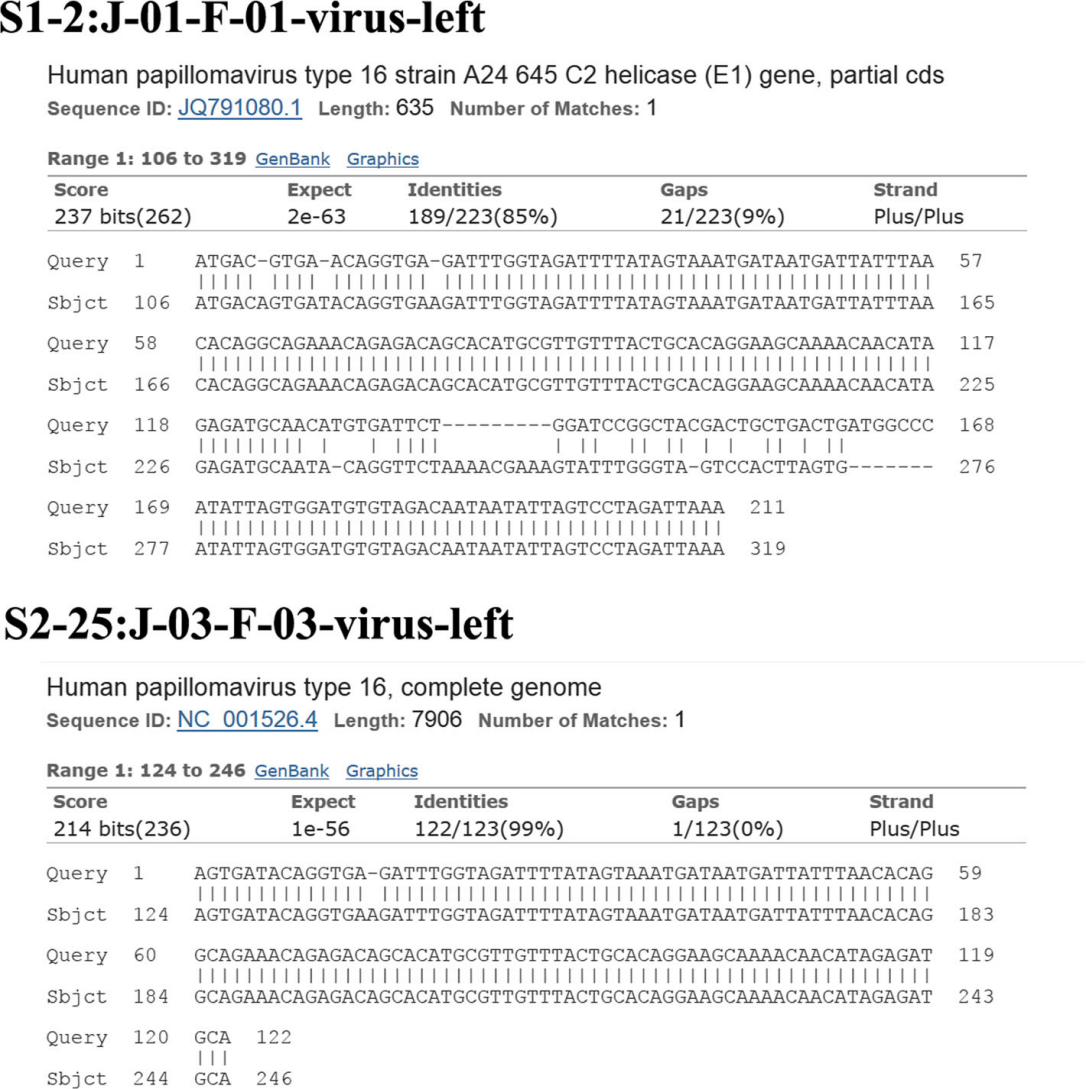
The alignment between inserted viral elements (S1-2:J-01-F-01-virus-left and S2-25:J-03-F-03-virus-left) and most closely related HPV16 sequences (HPV16 strain A24645 and HPV16 strain NC_001526.4). The “Query” indicates the viral sequences while the “sbjct” indicates the HPV16 sequences acquired from the NCBI database

In this study, we analyzed the variability of inserted HPV fragments, and found they showed a range of variability compared to the reference HPV16 strains. Briefly, the inserted viral elements were searched against NCBI database [31–33] and the alignment between the viral sequences and HPV16 sequences were checked manually to investigate the variability (Fig. 2 and Additional file 2: Figure S1). Among the 8 viral elements, 4 viral elements derived from *E1* gene, 2 from *L1* gene, 1 from *E2* gene and 1 from *E5* gene.

S1-2:J-01-F-01-virus-left was found to be most similar to the *E1* gene of HPV16 strain A24645 (GenBank: JQ791080.1). There were 12 nucleotides of deletions, 8 nucleotides of insertions and 11 nucleotides of mismatches. Notably, there was a insertion consisting of 7 consecutive nucleotides and a deletion comprising of 9 consecutive nucleotides in S1-2:J-01-F-01-virus-left.

S2-25:J-03-F-03-virus-left shared highest identity to the *E1* gene of HPV16 strain (GenBank: NC_001526.4). One nucleotide deletion was found in S2-25:J-03-F-03-virus-left compared to *E1* gene of HPV16 (NC_001526.4).

S2-25:J-04-virus-left was most similar to the *E1* gene of HPV16 strain NC_001526.4. One nucleotide deletion and one nucleotide mismatch was found in S2-25:J-03-F-03-virus-left compared to *E1* gene of the reference HPV (NC_001526.4).

C3-64:J-05-virus-left most resembled the *E2* gene of HPV16 isolate D15 (HM162476). 2 nucleotides of deletions, 2 nucleotides of insertions and 3 nucleotides of mismatches were found in C3-64:J-05-virus-left compared to *E1* gene of the reference HPV16 isolate D15.

C4-77:J-06-virus-right was more similar to the *L1* gene of HPV16 strain KU951195.1 than that any other published HPV strains. Single nucleotide deletion was found in C4-77:J-06-virus-right compared to *L1* gene of the HPV16 strain KU951195.1.

C5-87:J-07-virus-right was most similar to the *L1* gene of HPV16 isolate 16CN46 (GenBank: KU951194.1). 100% identity was found between C5-87:J-07-virus-right and *L1* gene of HPV16 isolate 16CN46, without any deletion, insertion or mismatch.

C5-87:J-08-virus-right was 100% identical to the corresponding region of *E5* gene of HPV16 isolate 16-Anhui12 from China (GenBank: KC935953.1). Again, no deletion, insertion or mismatch were detected.

S6-95:J-09-virus-left was most closely related to the *E1* gene of HPV16 isolate (GenBank: NC_001526). A deletion comprising of single nucleotide and a deletion consisting of 4 consecutive nucleotide were discovered in S6-95:J-09-virus-left, compared to the *E1* gene of HPV16 isolate (NC_001526).

## Discussion

### Variability of inserted viral fragments and possible outcomes of viral mutation

HPV were reported to exist in 2 forms: free episomes in the nucleus or integrated form in the cells genome. The transition from high-grade cervical intraepithelial neoplasia to micro-invasive carcinoma has been proposed to be characterized by the integration of HPV 16/18 [34]. The E2 protein is a transcriptional regulator for viral promoters located in the long control region, which negatively regulates the expression level of viral oncogenes (*E6* and *E7*) [35, 36]. The integration of HPV were found to coincide with the mutation of *E2* gene [37, 38]. In addition, the disruption of *E2* gene is reported as a common and early event during the Cervical HPV infection [39]. Among the 8 viral fragments identified in our study, half of them derived from *E1* gene of HPV16, 2 derived from *L2* gene, the remaining 2 viral elements were found to origin from *E2* and *E5* gene respectively. The C3-64:J-05-virus-left shared highest similarity with the *E2* gene of HPV16 isolate D15, with 3 nucleotides mismatch and 2 nucleotides insertion. The 3-nucleotide mismatches were expected to cause missense mutation while the 2 nucleotides insertion would lead to frame-shift mutation. Consequently, the functions of HPV E2 protein would be disrupted by those mutations. *E1* encodes for a protein which binds to the viral origin of replication, promoting the replication of viral genome. We found 4 cases of HPV16 *E1* gene integration coinciding with extensive mutations, probably leading to the disruption of the replication-promoting function of *E1* gene. L1 was proposed to self-assemble into pentameric capsomers and cooperate with L2 to package HPV DNA into virion. The surface loops of L1 were found to vary substantially even among members in the same papillomavirus species, probably facilitating its evasion of cellular immune responses [40]. We observed 2 cases of HPV *L1* integration, and viral protein mutation was found in one of them (C4-77:J-06-virus-right). E5 protein is reported destabilize many membrane proteins in HPV infected cells, probably preventing infected cell from being wiping out by killer T cells. The C5-87:J-08-virus-right derived from *E5* gene of HPV16, and no mutation was detected in the inserted viral *E5* element. In summary, 1 case of un-mutated *E5* gene (C5-87:J-08-virus-right) and 1 case of un-mutated *L1* gene fragment (C5-87:J-07-virus-right) were found to be inserted into cellular genome. 1 case of mutated *L1* gene (C4-77:J-06-virus-right),1 case of mutated *E2* gene fragment (C3-64:J-05-virus-left) and 4 cases of mutated *E1* gene fragments (S1-2:J-01-F-01-virus-left, S2-25:J-03-F-03-virus-left, S2-25:J-04-virus-left and C3-64:J-05-virus-left) were found to be integrated into host genome. Considering that E1 protein promotes the replication of viral genome and E2 protein negatively regulates the expression of onco-gene. The integration and mutation of *E1* and *E2* genes would probably inhibit the viral DNA replication while promoting viral onco-gene E6 and E7 expression. The mutation of L1 might lead to the change in the antigenic epitope in the capsid of HPV16.

### The HPV16 integration seem to occur around common fragile sites through micro homology-mediated DNA repair pathway

There is a heated debate regarding the integration mechanism of HPV. Some studies suggest that the HPV integration occurs at a random manner. However, more and more evidence have been proposed to challenge this idea. Wentzensen, N. et al. performed a systematic review on more than 190 reported HPV integration loci and confirmed that HPV integration sites are distributed over the chromosomes with a clear preference at genomic fragile sites [41]. In a study on 47 HPV16 and HPV18 positive cervical carcinoma, HPV were found to integrate in a non-random manner, preferably to hotspots region [42, 43]. Similarly, a high throughput screen on 3667 HPV integration breakpoints identified clustered genomic hot spots and this study indicates that HPV integration was likely to have occurred through micro homology-mediated DNA repair pathway, based on the evidence that micro homologous sequence was significantly enriched around integration sites [44]. In consistent with previous finding that the HPV sequence are more likely to integrate into the CFSs [43, 45]. In our results, among 8 integration sites identified, all of them occurred near the CFSs. In particular, CFS FRA22B site was affected twice. In addition, we observed short overlapping sequences between the viral and cellular fragments in 8 out of 11 cases of virus-cell fusions among the 8 junctions, and the overlapping DNA residuals range from 2 nt to 6 nt. The observation that all the HPV16 integration events occurred around CFSs combined with the phenomena that short overlapping nucleotides existed between viral and celluar derived fragments indicate that the fusion of HPV and cellular fragments probably happened through micro homology-mediated DNA repair pathway.

### The effect of HPV integration on the expression of cellular genes

**Fig. 3:**
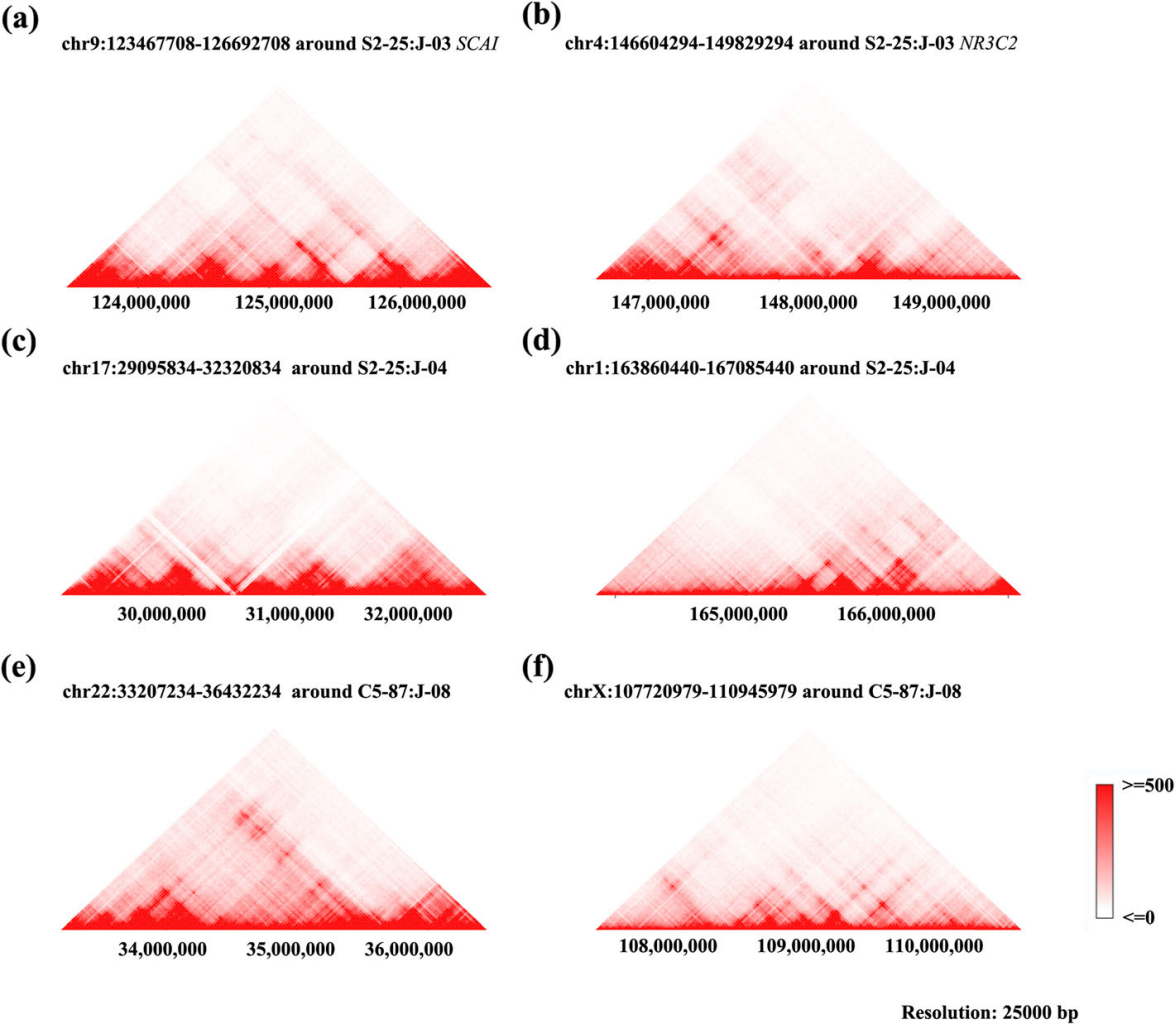
The Hi-C data showing that there are intensive chromosome interactions around chromosome regions within 3225000 bp of 6 HPV16 integrating sites corresponding to 3 chromosome translocation events (**a**-**f**) at the resolution of 25,000 bp. Hi-C data of GM12878 cell line was downloaded from the Interactive Hi-C Data Browser (http://promoter.bx.psu.edu/hi-c/) based on work of Rao, S. S. P. et al. [13]. The strengths of interaction between different genome regions are positively correlated to the red color (deep red for interaction score > =500, white for interaction score < =0)

After observing 3 cases of chromosome translocation, we next asked what effect it would have on those genes overlapped with integrating sites. Apparently, the gene structure would be interrupted by such translocation events. Taken *SCAI* and *NR3C2* for instance, *SCAI* gene is composed of 7 transcripts with the longest transcripts having 18 exons and 17 introns. *NR3C2* could be transcript into 8 mRNA isoforms with the longest one composed of 9 exons and 8 introns. As the breakpoints was located in the second intron of *SCAI* and the third intron of *NR3C2* genes. After translocation, *SCAI* would lose its last 16 exons and *NR3C2* would lose its first 3 exons. Therefore, the functions of both *SCAI* and *NR3C2* are expected to be largely disrupted, if not totally abolished. In the other two translocation events, there are no genes found to be directly overlapped with integrating sites, however, the translocation would likely disrupt the genes located nearby. Furthermore, the effects of those translocation events will not be limited to regions directly overlapped with or closely located. Instead, translocation might affect the global interaction architectures of translocated chromosomes. According to Hi-C data reported by Rao S, et al., there are extensive interactions between DNA cis-regulatory elements inter- and intra- chromosomes (Fig. 3) [14]. As the timing of gene expression of target genes is largely determined by its chromosome 3D architectures through epigenetic or transcriptional regulation. It might be quite possible that many master regulator genes and cell effectors genes will be altered by such translocation. As the normal growth of cells is controlled by a dedicated balance between oncogenes which promote cell proliferation and tumor suppressor genes which repress cell growth, it would be not surprising that the translocation between chromosomes could lead to substantial disruption to this balance and result in uncontrolled growth of cells.

In a systematic screen on more than 1500 integration sites of HPV, Bodelon et al. found few integration events happening in the neighborhood of cancer driver genes [10]. In our study, we discovered several integration sites located inside the introns of tumor suppressor genes (including the famous *SCAI* gene), implying a link between HPV integration and cervical oncogenesis, probably through manipulating the expression of cancer related genes. As introns are likely to accommodate multiple binding sites of several transcription factors [46–52], the integration of HPV would probably interrupt the original expression patterns of affected genes either through the introducing of novel transcription factors binding sites (TFBSs) or the disruption of existing TFBSs which located inside or around the breakpoints.

Peter, M. et al. performed a study on the HPV integration sites in 9 cell lines and found that HPV16 or 18 sequences were found to be integrated at chromosome 8q24, the location of proto-oncogene *MYC*. The *MYC* gene alteration and viral insertion were observed at the *MYC* locus in vivo in primary tumors [53]. Previous study suggests that HPV integration, even in intron regions, could affect gene expression and contribute to the complete loss of gene function in some occasions [54, 55]. In our study, the integration of HPV16 in genes have been identified in the introns of 4 genes (*SNX25*, *KALRN*, *NAALADL2* and *CDH13*) without chromosome translocation. Although the HPV16 integration would not disrupt the structures of exons, there might be quite a lot of cis regulatory elements such as enhancers or insulators located in intron regions, and the insertion of viral fragment would disrupt the interaction between TFs and cis regulatory elements and block the chromosome interactions. Therefore, the HPV16 integration without chromosome translocation would also affect the expression level of corresponding genes though the coding sequences might still keep intact. Overall, the impact of HPV16 integration could be profound and extensive. It would not only alter the expression of overlapping and nearby genes, but also reshape the architecture landscapes of affected chromosomes.

### Integration of HPV16 might contribute to cancer development through disruption tumor suppressor genes and inducing chromosome instability

Hanahan, D. & Weinberg, R. A suggest that there are 8 hallmarks of human cancer: the sustaining of proliferative signal, the evasion of growth suppressing, the resisting to cell death, the promoting of replication immortality, the inducing of angiogenesis process, the activating of metastasis, the reprogramming of energy metabolism process and the evading of immune destruction [56]. In our study, the integration of HPV16 seems to be linked to several those hallmarks, the sustaining of proliferative signal, the evasion of growth suppressing and immune destruction.

In our study, the integration of HPV16 combined with translocation is expected to disrupt the function of tumor suppresser gene *SCAI*. As a well-studied genes which have been shown to negatively regulate Rho protein signal transduction. Rho proteins play important roles in signal transduction which result in cytoskeletal-dependent responses such as cell migration and phagocytosis. Besides, Rho proteins are important regulators of matrix-degrading proteases which are crucial to cancer invasion [57]. Thus the disruption of *SCAI* gene would abolish its repression on Rho protein mediated signal transduction and lead to the facilitating of cell migration, which contribute substantially to the metastasis of tumor. In consistent with this hypothesis, Brandt, D. T. et al. proposed that *SCAI* regulates the migration of invasive cells via cell matrices based on the observation that the expression level of *SCAI* was negatively correlated with the degree of invasive cell migration, and *SCAI* was found to be down regulated in several human tumours [58]. Camilla Kreßner found that the expression of *SCAI* diminished in a variety of primary human breast cancer samples [59].

Based on the GO annotation of Kalirin protein (O60229) in UniProt database [60], it was predicted to involve in positive regulation of apoptotic process which triggers the apoptotic death of a cell (GO:0043065). It was also predicted to regulates Rho protein signal transduction (GO:0035023), which leads to cytoskeletal-dependent responses such as cell migration [57]. Therefore, Kalirin might be related to two key factors of oncogenesis: the ability to migrate and escape apoptosis.

*Sorting nexin 25 gene* (*SNX25*) is also a cancer related gene, which is localized in the cytoplasm and could affect membrane association. *SNX25* gene has the phosphatidylinositol binding and type I transforming growth factor beta receptor binding activity according to its GO annotation. It also has signal transducer activity (GO: 0004871). *SNX25* is reported to be able to negative regulate transforming growth factor beta receptor signaling pathway. In addition, SNX25 was reported to regulate TGF-β signaling through enhancing the receptor degradation [61].

*N-acetyl*-*l*-*aspartyl*-*l*-*glutamate peptidase*-*like 2* (*NAALADL2*) belongs to glutamate carboxypeptidase II family, NAALADL2 has been reported to localize to the basal cell surface and affect the adhesion, migration and invasion of tumor cells. In addition, NAALADL2 also regulate the expression of master regulator (Ser133 phosphorylated C-AMP-binding protein) of cellular processes involved in the development and progression of cancer [62].

*Cadherin 13* (*CDH13*) gene encodes cadherin cellular adhesion molecular, which localized to the cell membrane surface. CDH13 is related to ERK Signaling and Nanog pathway, and its GO annotations include ‘calcium ion binding and cadherin binding’. The expression level of *CDH13* was found to be significantly reduced in breast cancer specimen and breast carcinoma cell lines [63].

**Fig. 4:**
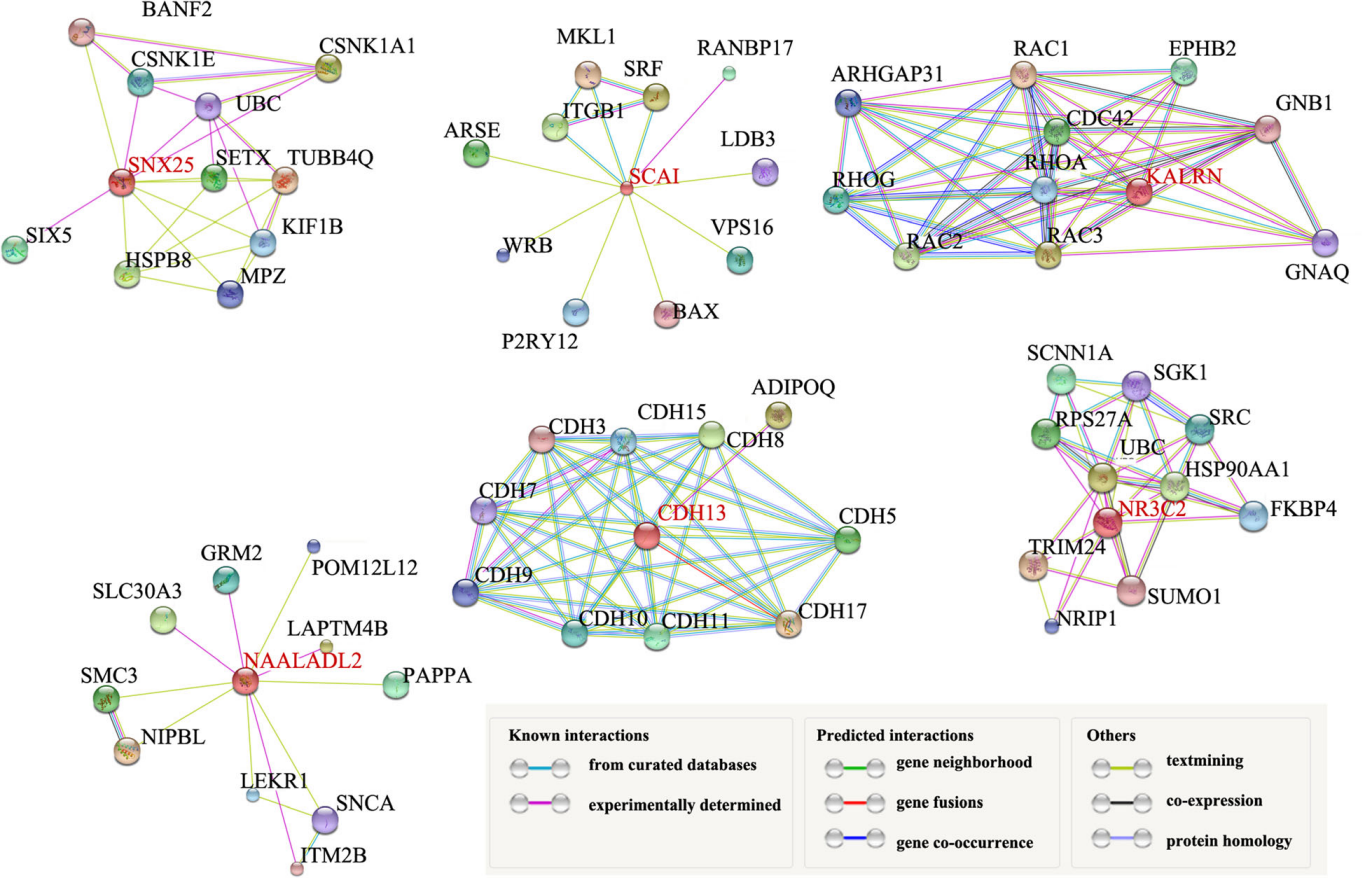
Interaction network of 6 proteins (SNX25, SCAI, KALRN, NAALADL2, CDH13 and NR3C2) encoded by genes directly overlapping with 8 HPV16 integration sites. The protein-protein interaction networks were downloaded from STRING database [13]

In addition to inferring the potential biological effects posed by HPV16 integration on those overlapping genes, we also analyzed the protein-protein interaction network of those 6 proteins (SNX25, SCAI, KALRN, NAALADL2, CDH13 and NR3C2) based on the interaction relationships annotated in STRING database [13]. It indicates that those 6 proteins interact with several effectors or master regulators, involving in a variety of pathways or biological processes (Fig. 4). Therefore, the disruption of those 6 proteins would undermine those protein-protein interaction relationships and interrupt the pathways and biological functions regulated by those proteins.

Genomic instability is a common characteristic shared by most cancers [64–66]. Previous studies suggest that human chromosomal instability or insertion mutagenesis by integrated viral sequences may damage the key hotspots oncology-related genes and cause the structural and numerical chromosome changes [67–69]. In our study, we only observed three cases of chromosome translocation events coincide with HPV integration. However, we assume there are other chromosome variations such as genome insertion/deletion, gene copy number variation though they were impossible to be observed by us as we only focused on investigation to study the integration of HPV16 into human genome and the subsequent fusion between viral genome fragment and cellular fragment. Nevertheless, we need to point out that the chromosome translocation we observed is just the tip of the iceberg, which serves a good indicator of chromosome instability at genomic level. The observation that genes around HPV16 integrating sites are enriched in GO terms associated with DNA repair is particularly interesting. If the expression level of DNA repair genes were affected by HPV16 integration. Then we would expect to see accelerated genome instability due to lacking of repaired for damaged or mutated DNA. Therefore, we could deduce that HPV16 integration is likely to result in chromosome instability by combining two pieces of independent evidence: the chromosome translocation events around HPV16 integrating sites, and the enrichment of DNA repair genes within 10 Mb of HPV integrating sites.

Intriguingly, we also observed the enrichment of immune related genes (GO: 0071346 cellular response to interferon-gamma and GO:0034341 response to interferon-gamma) around HPV16 integrating sites. Interferon-gamma has been reported to be a cytokine that promotes both innate and adaptive immune responses. As a crucial immune response modifier which could upregulate immune destruction against tumor, the integration by HPV16 might undermine its protection against cancer development and tumor immunoediting [38]. In addition, GO: 0002548 monocyte chemotaxis’ is also found to be enriched. Considering that chemotaxis of tumor cells is essential for tumor dissemination during the process of tumor progression and metastasis [70], we are tempted to predict that HPV16 integration might affect the migration and metastasis of tumor cells.

**Fig. 5:**
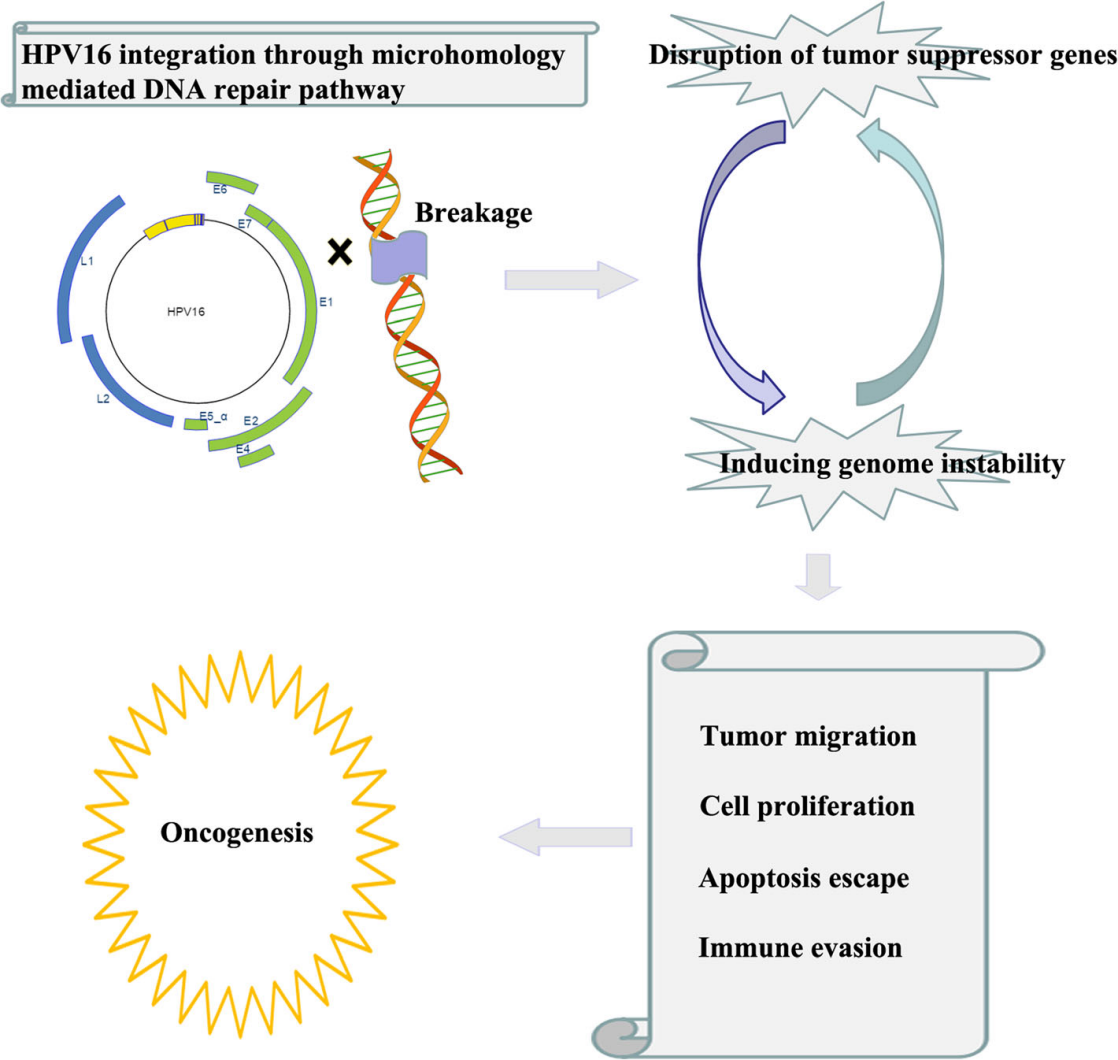
HPV16 integration probably contributes to tumor progression through disrupting tumor suppressor gene expression and inducing chromosome instability

Previous reports suggest that the main function of HPV integration is to stabilize the expression of viral oncogenes instead of disruption of cellular genes [71]. However, our study suggest that manipulating the expression of cellular genes, especially cancer related genes, and inducing chromosome instability might play much more significant roles for the progress from lesions to invasive cancers than previously thought based on following observations: 1) Cancer related genes, especially tumor suppressor genes were integrated by HPV16 and combined. 2) Genes directly overlapping with HPV16 integration sites were known or predicted to involve in a variety of biological processes regarding the adhesions, apoptosis, proliferation, migration, immune evasion of tumor cells. 3) The genes around HPV16 integrations sites were enriched in GO terms related to the immune response, DNA repair, tumor migration and the transfer of genetic materials. 4) There were extensive chromosome interactions in regions around HPV16 integrating sites. 5) Chromosome translocation events around HPV16 integration sites were detected through sequencing, which indicates there might exist widespread genomic variations induced by HPV16 integration. Taken together, our study suggests that HPV16 integration probably contributes to tumor development, through disruption tumor repressor genes and inducing chromosome instability (Fig. 5).

## Conclusion

In the current work, we focus on integration sites analysis of HPV16, the most common type of HPV in Shanghai, trying to understand the HPV16 integration characteristics and the potential consequence of viral fragment integration. We found that 8 HPV16 integration sites inside or around genes associated with cancer development. On top of that, we observed several cases of chromosome translocation events coincide with HPV integration, which suggests the existence of chromosome instability. Additionally, short overlapping sequences were observed between viral derived and host derived fragments in viral-cellular junctions, indicating that integration was mediated by micro homology-mediated DNA repair pathway. Overall, our study suggests a model in which HPV16 might contribute to oncogenesis not only by disrupting tumor suppressor genes, but also by inducing chromosome instability. Nevertheless, it should be noted that our hypothesis is simply based on the analysis of relatively a small amount of HPV16 positive samples, which should be interpreted cautiously. Comprehensive analysis on large scale HPV screening experiments needs to be conducted to confirm this hypothesis.

## Additional files

Additional file 1:
**File S1.** Viral or cellular derived sequences in virus-human junctions. **Table S1.** List of genes located in 1 mega bases of HPV16 integration sites. **Table S2.** List of genes located in 10 mega bases of integration sites. (DOC 1198 kb)
Additional file 2: Figure S1. The alignment between inserted viral elements (S2-25:J-04-virus-left, C3-64:J-05-virus-left, C4-77:J-06-virus-right, C5-87:J-07-virus-right, C5-87:J-08-virus-right, S6-95:J-09-virus-left) and most closely related HPV16 sequences. The “Query” indicates the viral sequences while the “sbjct” indicates the HPV16 sequences acquired from the NCBI database. (TIF 3984 kb)
