# Supplementary material for: HPV16 integration probably contributes to cervical oncogenesis through interrupting tumor suppressor genes and inducing chromosome instability
Source: J Exp Clin Cancer Res. 2016 Nov 25;35:180. doi: 10.1186/s13046-016-0454-4 (PMC5123399; doi:10.1186/s13046-016-0454-4)
Supplement: Additional file 1: — File S1. Viral or cellular derived sequences in virus-human junctions. Table S1. List of genes located in 1 mega bases of HPV16 integration sites. Table S2. List of genes located in 10 mega bases of integration sites. (DOC 1198 kb) [file 13046_2016_454_MOESM1_ESM.doc]

### HPV16 integration probably contributes to oncogenesis through interrupting [tumor suppressor genes](http://www.ncbi.nlm.nih.gov/pubmed/2573189) and inducing chromosome instability

###

Jun-Wei Zhao, Fang Fang, Yi Guo, Tai-Lin Zhu, Yun-Yun Yu, Fan-Fei Kong, Ling-Fei Han, Fang Li, Dong-Sheng Chen

[Supplementary file 1. Viral or cellular derived sequences in virus-human junctions 2](#__RefHeading___Toc451085485)

[Table S1. List of genes located in 1 mega bases of HPV16 integration sites 4](#__RefHeading___Toc451085486)

[Table S2. List of genes located in 10 mega bases of integration sites 7](#__RefHeading___Toc451085487)

# Supplementary file 1. Viral or cellular derived sequences in virus-human junctions

>S1-2:J-01-F-01-virus-left

ATGACGTGAACAGGTGAGATTTGGTAGATTTTATAGTAAATGATAATGATTATTTAACACAGGCAGAAACAGAGACAGCACATGCGTTGTTTACTGCACAGGAAGCAAAACAACATAGAGATGCAACATGTGATTCTGGATCCGGCTACGACTGCTGACTGATGGCCCATATTAGTGGATGTGTAGACAATAATATTAGTCCTAGATTAAA

>S1-2:J-01-F-01-human-right

GCTTTTCTGGGTTGGACATGGGAGTAAAGCGGGGGTGAAAATATAATAAGCTATCAATTATTG

>S2-25:J-03-F-03-virus-left

AGTGATACAGGTGAGATTTGGTAGATTTTATAGTAAATGATAATGATTATTTAACACAGGCAGAAACAGAGACAGCACATGCGTTGTTTACTGCACAGGAAGCAAAACAACATAGAGATGCA

>S2-25:J-03-F-03-human-middle

GATGCATACTATGAGGGTCCTTTGGGAAGAAGAACATGACTTTGGGGTTTTTTTTCTAACTGTGTAATTCTGGAGAAATAACTTACTTAAGCCTGTTTTTTCATCTCTAAATTCTGGTGACAGTGCCTACCTTATAGGTGTGTGCAGATGATATGTATATAAAATGCCTGGCATAGTTTCTGGAGCGTGGGAGCTTGATTTCTTCTGTACCCTGCCCCCTGCCTTTTTCTTCCTCCTTCCAGCTACAAGTGACTAAGGATC

>S2-25:J-03-F-03-human-right

GATCATGAGCTAGGTAACATGTTCAGATAGCAGGGTGATTTGACATTCAAGATAAATAAGTTGTTTATCAGTAGGACCATGTCTGATACTACTTAGTCAACAAAGCAAAGATCCAACGTGTAAGTCTGGATCCGGCTACGACTGCTGCTGGAATGGCCA

>S2-25:J-04-virus-left

AGTGATACAGGTGAGATTTGGTAGATTTTATAGTAAATGATAATGATTATTTAACACAGGCAGAAACAGAGACAGCACATGCGTTGTTTACTGCACAGGAAGCAAAACAACATAGAGATGCAGTACAGGTTCTAAAACGAAAGTATTTGGGTAGTCCACTTAGTGATATTAGTGGATGTGTAAAC

>S2-25:J-04-human-middle

GGGGCAGGGGCATAAGGAGGGCTGGACTCGGGTACAAGTGGATTCGGATTACTGGCTGCACCGAAGCGGACAGGTAAAAGCGGTTTCTAGATC

>S2-25:J-04-human-right

AGATCTTCATTTTACTGCCGCTCCTGCTGGTGACTAATATTCCTGGGACATGTTATTTAACCTCTCTGAGGCTCATGGTTCTAATTTTCAAATCAGTGATGATAAACCTTTGAGAGATGCAGGTTTATTGTGAGGTGAAAGAATGACGGTTCCTGATACAAAAAAGAATCCTACAAATATTCGTTTTCCTCCTCTTGTTCCACTGCTCCCCATTCAGGTCCTGAACAAACTAAGGAATTGGCCCAACATCCCCCTACCTCCCAGTTGTGCCGATGAAGCTCTGGGACAAGTGGTTCGCCCTCACAACCAAGCAGCACTGATC

>C3-64:J-05-virus-left

TTTGCCCCGTTTAATGTGTGTCAGGACAAATACTAACACATTGATGAAAATGATAGTACATTACCTACGTGACCATATAGACTATTGAAAACACATGCGCCTAGAATGTGCTATTTATTACAAGGCCAGAGAAATGGGATTTAAACATATTAACCACCAGGTGGTGCCAACACTGGCTGTATCAAAGAATAAAGCATTACAAGCAATTGAACTGCAACTAACGTTAGAAACAATATATAACTCACAATATAGTAATGAAAAGTGGACATTACAAGACGTTAGCCTTGAAGTGTATTTAACTGCACCAACAGGATGTATAAAAAAA

>C3-64:J-05-human-right

AATGAAGCCAGAATTTAATCTAGCTTTGACTCTTCAGCATTCTTTGATTTCTTTCCATTATGTCTCTCACCAGCTTCAAATTGTAAATGGTTTGCTACTACCCCTGGAATAATGATAAAACTCAAGGTACTGCTATCGTTTGTCCTCATCTAAGTCAATTTTACTTCAAAATACTTTCTAGTTCAGATC

> C4-77:J-06-human-left

ACAGCATCACAGAAATCCTGCAACAGCGACGCCTCAAAAGAGGCTGAGAAAGGTAAAGACTGAGAGGAAGCC

> C4-77:J-06-virus-right

CGAGCACAGGGCCACAATAATGGCATTTGTTGGGGTAACCAACTATTTGTTACTGTTGTTGATACTACACGCAGTACAAATATGTCATTATGTGCTGCCATATCTACTTCAGAAACTACATATAAAAATACTAACTTTAAGGAGTACCTACGACATGGGGAGGAATATGATTTACAGTTTATTTTTCAACTGTGCAAAATAACCTTAACTGCAGACGTTATGACATACATACATTCTATGAATTCCACTATTTTGGAGGACTGGAATTTTGGTCTACAACCTCCCCCAGGAGGCACACTAGAAGTACTTA

> C5-87:J-07-human-left

GATCTCTCCTTCCACAGTGTTTTTCACAGTCCTCCAAGGTACAAATCATCTTGCTGTGCTGAATTGCCTTTTGGCTTGACTGTCACTCCAATA

> C5-87:J-07-virus-right

CAATAACAAAATATTAGTTCCTAAAGTATCAGGATTACAATACAGGGTATTTAGAATACATTTACCTGACCCCAATAAGTTTGGTTTTCCTGACACCTCATTTTATAATCCAGATACACAGCGGCTGGTTTGGGCCTGTGTAGGTGTTGAGGTAGGTCGTGGTCAGCCATTAGGTGTGGGCATTAGTGGCCATCCTTTATTAAATAAATTGGATGACACAGAAA

> C5-87:J-08-human-left

GATCTTTCCTGCTTTCTTTCGTGGGCATTTAATGCTATAAATTTCCCTCTACACACTGCTTTAAATGTATCCCAGAGATTCTGGTATGTTGTGTCTTTGTTCTCATTGGTTTCAAAGAACATCTTTATTTCTGCCTTCATTTCATTATGTACCCAGTATTCATTCAGGAGCAGGTTTTTCAGTTTCCATGTAGTTGAGTGG

>C5-87:J-08-human-middle

GTGGGAGTCTAAGTCTCTTTGTAGGTCTCTAAGGACTTGCTTTATGAATCTGGGTGCTCCTGTGTT

> C5-87:J-08-virus-right

GTGTTTTATTGTATATATTGTATTTGTTTATATACCATTATTTTTAATACATACTCATGCACGCTTTTTAATTACATAATGTATATGTACATAATGTAATTGTTACATATAATTGTTGTATACCATAACTTACTATTTTTTCTTTTTTATTTTTATATATAATTTTTTGTTTGTTTGTGTGTTTGTTTTTTAATAAACTGTTATCACTTAACAATGCGACACAAACGTTCTGCAAAACGCACAAAACGTGCATCGGCTACCCAACTTTATAAAACATGCAAACAGGCAGGTACATGTCC

> S6-95:J-09-virus-left

AGTGATACAGGTGAGATTTGGTAGATTTTATAGTAAATGATAATGATTATTTAACACAGGCAGAAACAGAGACAGCACATGCGTTGTTTACTGCACAGGAAGCAAAACAACATAGAGATGCAGTACAGGTTCTAAAACGAAAGTATTTGGGTAGTCCACTTAGTGATATTAGTGGATGTGTAGACAATAATATTAGTCCTAGATTAAAAGCTATATGTATAGAAAAACAAAGGCTGCAA

> S6-95:J-09-human-right

CTGCAACCTCCGCCTCCCGAGTTCAAGTGACTCTCCTGCCTCAGCCTCCCCAGTAGCTAGGATTACAGGCACTTGCCTGGCTAATGTTTGTATTTTTAGTAAAGACAGAGTTTCACCATGTTGGCCAGGCTGGTCTCCAACTCCTGACCTCGTGATCCA

# Table S1. List of genes located in 1 mega bases of HPV16 integration sites

| **Ensembl Gene ID** | **Associated Gene Name** |
| --- | --- |
| [ENSG00000143183](http://www.ensembl.org/Homo_sapiens/Gene/Summary?db=core;g=ENSG00000143183) | [TMCO1](http://www.ensembl.org/Homo_sapiens/Gene/Summary?db=core;g=ENSG00000143183) |
| [ENSG00000162763](http://www.ensembl.org/Homo_sapiens/Gene/Summary?db=core;g=ENSG00000162763) | [LRRC52](http://www.ensembl.org/Homo_sapiens/Gene/Summary?db=core;g=ENSG00000162763) |
| [ENSG00000143149](http://www.ensembl.org/Homo_sapiens/Gene/Summary?db=core;g=ENSG00000143149) | [ALDH9A1](http://www.ensembl.org/Homo_sapiens/Gene/Summary?db=core;g=ENSG00000143149) |
| [ENSG00000162761](http://www.ensembl.org/Homo_sapiens/Gene/Summary?db=core;g=ENSG00000162761) | [LMX1A](http://www.ensembl.org/Homo_sapiens/Gene/Summary?db=core;g=ENSG00000162761) |
| [ENSG00000143171](http://www.ensembl.org/Homo_sapiens/Gene/Summary?db=core;g=ENSG00000143171) | [RXRG](http://www.ensembl.org/Homo_sapiens/Gene/Summary?db=core;g=ENSG00000143171) |
| [ENSG00000143198](http://www.ensembl.org/Homo_sapiens/Gene/Summary?db=core;g=ENSG00000143198) | [MGST3](http://www.ensembl.org/Homo_sapiens/Gene/Summary?db=core;g=ENSG00000143198) |
| [ENSG00000143179](http://www.ensembl.org/Homo_sapiens/Gene/Summary?db=core;g=ENSG00000143179) | [UCK2](http://www.ensembl.org/Homo_sapiens/Gene/Summary?db=core;g=ENSG00000143179) |
| [ENSG00000140945](http://www.ensembl.org/Homo_sapiens/Gene/Summary?db=core;g=ENSG00000140945) | [CDH13](http://www.ensembl.org/Homo_sapiens/Gene/Summary?db=core;g=ENSG00000140945) |
| [ENSG00000108578](http://www.ensembl.org/Homo_sapiens/Gene/Summary?db=core;g=ENSG00000108578) | [BLMH](http://www.ensembl.org/Homo_sapiens/Gene/Summary?db=core;g=ENSG00000108578) |
| [ENSG00000108576](http://www.ensembl.org/Homo_sapiens/Gene/Summary?db=core;g=ENSG00000108576) | [SLC6A4](http://www.ensembl.org/Homo_sapiens/Gene/Summary?db=core;g=ENSG00000108576) |
| [ENSG00000176390](http://www.ensembl.org/Homo_sapiens/Gene/Summary?db=core;g=ENSG00000176390) | [CRLF3](http://www.ensembl.org/Homo_sapiens/Gene/Summary?db=core;g=ENSG00000176390) |
| [ENSG00000108582](http://www.ensembl.org/Homo_sapiens/Gene/Summary?db=core;g=ENSG00000108582) | [CPD](http://www.ensembl.org/Homo_sapiens/Gene/Summary?db=core;g=ENSG00000108582) |
| [ENSG00000176208](http://www.ensembl.org/Homo_sapiens/Gene/Summary?db=core;g=ENSG00000176208) | [ATAD5](http://www.ensembl.org/Homo_sapiens/Gene/Summary?db=core;g=ENSG00000176208) |
| [ENSG00000196712](http://www.ensembl.org/Homo_sapiens/Gene/Summary?db=core;g=ENSG00000196712) | [NF1](http://www.ensembl.org/Homo_sapiens/Gene/Summary?db=core;g=ENSG00000196712) |
| [ENSG00000182271](http://www.ensembl.org/Homo_sapiens/Gene/Summary?db=core;g=ENSG00000182271) | [TMIGD1](http://www.ensembl.org/Homo_sapiens/Gene/Summary?db=core;g=ENSG00000182271) |
| [ENSG00000181481](http://www.ensembl.org/Homo_sapiens/Gene/Summary?db=core;g=ENSG00000181481) | [RNF135](http://www.ensembl.org/Homo_sapiens/Gene/Summary?db=core;g=ENSG00000181481) |
| [ENSG00000172171](http://www.ensembl.org/Homo_sapiens/Gene/Summary?db=core;g=ENSG00000172171) | [TEFM](http://www.ensembl.org/Homo_sapiens/Gene/Summary?db=core;g=ENSG00000172171) |
| [ENSG00000184060](http://www.ensembl.org/Homo_sapiens/Gene/Summary?db=core;g=ENSG00000184060) | [ADAP2](http://www.ensembl.org/Homo_sapiens/Gene/Summary?db=core;g=ENSG00000184060) |
| [ENSG00000108587](http://www.ensembl.org/Homo_sapiens/Gene/Summary?db=core;g=ENSG00000108587) | [GOSR1](http://www.ensembl.org/Homo_sapiens/Gene/Summary?db=core;g=ENSG00000108587) |
| [ENSG00000175329](http://www.ensembl.org/Homo_sapiens/Gene/Summary?db=core;g=ENSG00000175329) | [ISX](http://www.ensembl.org/Homo_sapiens/Gene/Summary?db=core;g=ENSG00000175329) |
| [ENSG00000100284](http://www.ensembl.org/Homo_sapiens/Gene/Summary?db=core;g=ENSG00000100284) | [TOM1](http://www.ensembl.org/Homo_sapiens/Gene/Summary?db=core;g=ENSG00000100284) |
| [ENSG00000100281](http://www.ensembl.org/Homo_sapiens/Gene/Summary?db=core;g=ENSG00000100281) | [HMGXB4](http://www.ensembl.org/Homo_sapiens/Gene/Summary?db=core;g=ENSG00000100281) |
| [ENSG00000160145](http://www.ensembl.org/Homo_sapiens/Gene/Summary?db=core;g=ENSG00000160145) | [KALRN](http://www.ensembl.org/Homo_sapiens/Gene/Summary?db=core;g=ENSG00000160145) |
| [ENSG00000082781](http://www.ensembl.org/Homo_sapiens/Gene/Summary?db=core;g=ENSG00000082781) | [ITGB5](http://www.ensembl.org/Homo_sapiens/Gene/Summary?db=core;g=ENSG00000082781) |
| [ENSG00000065371](http://www.ensembl.org/Homo_sapiens/Gene/Summary?db=core;g=ENSG00000065371) | [ROPN1](http://www.ensembl.org/Homo_sapiens/Gene/Summary?db=core;g=ENSG00000065371) |
| [ENSG00000175455](http://www.ensembl.org/Homo_sapiens/Gene/Summary?db=core;g=ENSG00000175455) | [CCDC14](http://www.ensembl.org/Homo_sapiens/Gene/Summary?db=core;g=ENSG00000175455) |
| [ENSG00000173702](http://www.ensembl.org/Homo_sapiens/Gene/Summary?db=core;g=ENSG00000173702) | [MUC13](http://www.ensembl.org/Homo_sapiens/Gene/Summary?db=core;g=ENSG00000173702) |
| [ENSG00000177694](http://www.ensembl.org/Homo_sapiens/Gene/Summary?db=core;g=ENSG00000177694) | [NAALADL2](http://www.ensembl.org/Homo_sapiens/Gene/Summary?db=core;g=ENSG00000177694) |
| [ENSG00000114491](http://www.ensembl.org/Homo_sapiens/Gene/Summary?db=core;g=ENSG00000114491) | [UMPS](http://www.ensembl.org/Homo_sapiens/Gene/Summary?db=core;g=ENSG00000114491) |
| [ENSG00000168491](http://www.ensembl.org/Homo_sapiens/Gene/Summary?db=core;g=ENSG00000168491) | [CCDC110](http://www.ensembl.org/Homo_sapiens/Gene/Summary?db=core;g=ENSG00000168491) |
| [ENSG00000205129](http://www.ensembl.org/Homo_sapiens/Gene/Summary?db=core;g=ENSG00000205129) | [C4orf47](http://www.ensembl.org/Homo_sapiens/Gene/Summary?db=core;g=ENSG00000205129) |
| [ENSG00000154553](http://www.ensembl.org/Homo_sapiens/Gene/Summary?db=core;g=ENSG00000154553) | [PDLIM3](http://www.ensembl.org/Homo_sapiens/Gene/Summary?db=core;g=ENSG00000154553) |
| [ENSG00000187821](http://www.ensembl.org/Homo_sapiens/Gene/Summary?db=core;g=ENSG00000187821) | [HELT](http://www.ensembl.org/Homo_sapiens/Gene/Summary?db=core;g=ENSG00000187821) |
| [ENSG00000109762](http://www.ensembl.org/Homo_sapiens/Gene/Summary?db=core;g=ENSG00000109762) | [SNX25](http://www.ensembl.org/Homo_sapiens/Gene/Summary?db=core;g=ENSG00000109762) |
| [ENSG00000154556](http://www.ensembl.org/Homo_sapiens/Gene/Summary?db=core;g=ENSG00000154556) | [SORBS2](http://www.ensembl.org/Homo_sapiens/Gene/Summary?db=core;g=ENSG00000154556) |
| [ENSG00000151729](http://www.ensembl.org/Homo_sapiens/Gene/Summary?db=core;g=ENSG00000151729) | [SLC25A4](http://www.ensembl.org/Homo_sapiens/Gene/Summary?db=core;g=ENSG00000151729) |
| [ENSG00000186352](http://www.ensembl.org/Homo_sapiens/Gene/Summary?db=core;g=ENSG00000186352) | [ANKRD37](http://www.ensembl.org/Homo_sapiens/Gene/Summary?db=core;g=ENSG00000186352) |
| [ENSG00000071205](http://www.ensembl.org/Homo_sapiens/Gene/Summary?db=core;g=ENSG00000071205) | [ARHGAP10](http://www.ensembl.org/Homo_sapiens/Gene/Summary?db=core;g=ENSG00000071205) |
| [ENSG00000109775](http://www.ensembl.org/Homo_sapiens/Gene/Summary?db=core;g=ENSG00000109775) | [UFSP2](http://www.ensembl.org/Homo_sapiens/Gene/Summary?db=core;g=ENSG00000109775) |
| [ENSG00000109771](http://www.ensembl.org/Homo_sapiens/Gene/Summary?db=core;g=ENSG00000109771) | [LRP2BP](http://www.ensembl.org/Homo_sapiens/Gene/Summary?db=core;g=ENSG00000109771) |
| [ENSG00000151623](http://www.ensembl.org/Homo_sapiens/Gene/Summary?db=core;g=ENSG00000151623) | [NR3C2](http://www.ensembl.org/Homo_sapiens/Gene/Summary?db=core;g=ENSG00000151623) |
| [ENSG00000136933](http://www.ensembl.org/Homo_sapiens/Gene/Summary?db=core;g=ENSG00000136933) | [RABEPK](http://www.ensembl.org/Homo_sapiens/Gene/Summary?db=core;g=ENSG00000136933) |
| [ENSG00000136935](http://www.ensembl.org/Homo_sapiens/Gene/Summary?db=core;g=ENSG00000136935) | [GOLGA1](http://www.ensembl.org/Homo_sapiens/Gene/Summary?db=core;g=ENSG00000136935) |
| [ENSG00000173611](http://www.ensembl.org/Homo_sapiens/Gene/Summary?db=core;g=ENSG00000173611) | [SCAI](http://www.ensembl.org/Homo_sapiens/Gene/Summary?db=core;g=ENSG00000173611) |
| [ENSG00000044574](http://www.ensembl.org/Homo_sapiens/Gene/Summary?db=core;g=ENSG00000044574) | [HSPA5](http://www.ensembl.org/Homo_sapiens/Gene/Summary?db=core;g=ENSG00000044574) |
| [ENSG00000136950](http://www.ensembl.org/Homo_sapiens/Gene/Summary?db=core;g=ENSG00000136950) | [ARPC5L](http://www.ensembl.org/Homo_sapiens/Gene/Summary?db=core;g=ENSG00000136950) |
| [ENSG00000119487](http://www.ensembl.org/Homo_sapiens/Gene/Summary?db=core;g=ENSG00000119487) | [MAPKAP1](http://www.ensembl.org/Homo_sapiens/Gene/Summary?db=core;g=ENSG00000119487) |
| [ENSG00000119414](http://www.ensembl.org/Homo_sapiens/Gene/Summary?db=core;g=ENSG00000119414) | [PPP6C](http://www.ensembl.org/Homo_sapiens/Gene/Summary?db=core;g=ENSG00000119414) |
| [ENSG00000136918](http://www.ensembl.org/Homo_sapiens/Gene/Summary?db=core;g=ENSG00000136918) | [WDR38](http://www.ensembl.org/Homo_sapiens/Gene/Summary?db=core;g=ENSG00000136918) |
| [ENSG00000136942](http://www.ensembl.org/Homo_sapiens/Gene/Summary?db=core;g=ENSG00000136942) | [RPL35](http://www.ensembl.org/Homo_sapiens/Gene/Summary?db=core;g=ENSG00000136942) |
| [ENSG00000165219](http://www.ensembl.org/Homo_sapiens/Gene/Summary?db=core;g=ENSG00000165219) | [GAPVD1](http://www.ensembl.org/Homo_sapiens/Gene/Summary?db=core;g=ENSG00000165219) |
| [ENSG00000148200](http://www.ensembl.org/Homo_sapiens/Gene/Summary?db=core;g=ENSG00000148200) | [NR6A1](http://www.ensembl.org/Homo_sapiens/Gene/Summary?db=core;g=ENSG00000148200) |
| [ENSG00000185585](http://www.ensembl.org/Homo_sapiens/Gene/Summary?db=core;g=ENSG00000185585) | [OLFML2A](http://www.ensembl.org/Homo_sapiens/Gene/Summary?db=core;g=ENSG00000185585) |
| [ENSG00000101888](http://www.ensembl.org/Homo_sapiens/Gene/Summary?db=core;g=ENSG00000101888) | [NXT2](http://www.ensembl.org/Homo_sapiens/Gene/Summary?db=core;g=ENSG00000101888) |
| [ENSG00000101890](http://www.ensembl.org/Homo_sapiens/Gene/Summary?db=core;g=ENSG00000101890) | [GUCY2F](http://www.ensembl.org/Homo_sapiens/Gene/Summary?db=core;g=ENSG00000101890) |
| [ENSG00000068366](http://www.ensembl.org/Homo_sapiens/Gene/Summary?db=core;g=ENSG00000068366) | [ACSL4](http://www.ensembl.org/Homo_sapiens/Gene/Summary?db=core;g=ENSG00000068366) |
| [ENSG00000176076](http://www.ensembl.org/Homo_sapiens/Gene/Summary?db=core;g=ENSG00000176076) | [KCNE5](http://www.ensembl.org/Homo_sapiens/Gene/Summary?db=core;g=ENSG00000176076) |

# Table S2. List of genes located in 10 mega bases of integration sites

| **Ensembl Gene ID** | **Associated Gene Name** |
| --- | --- |
| [ENSG00000158869](http://www.ensembl.org/Homo_sapiens/Gene/Summary?db=core;g=ENSG00000158869) | [FCER1G](http://www.ensembl.org/Homo_sapiens/Gene/Summary?db=core;g=ENSG00000158869) |
| [ENSG00000117475](http://www.ensembl.org/Homo_sapiens/Gene/Summary?db=core;g=ENSG00000117475) | [BLZF1](http://www.ensembl.org/Homo_sapiens/Gene/Summary?db=core;g=ENSG00000117475) |
| [ENSG00000143183](http://www.ensembl.org/Homo_sapiens/Gene/Summary?db=core;g=ENSG00000143183) | [TMCO1](http://www.ensembl.org/Homo_sapiens/Gene/Summary?db=core;g=ENSG00000143183) |
| [ENSG00000203740](http://www.ensembl.org/Homo_sapiens/Gene/Summary?db=core;g=ENSG00000203740) | [METTL11B](http://www.ensembl.org/Homo_sapiens/Gene/Summary?db=core;g=ENSG00000203740) |
| [ENSG00000143147](http://www.ensembl.org/Homo_sapiens/Gene/Summary?db=core;g=ENSG00000143147) | [GPR161](http://www.ensembl.org/Homo_sapiens/Gene/Summary?db=core;g=ENSG00000143147) |
| [ENSG00000143217](http://www.ensembl.org/Homo_sapiens/Gene/Summary?db=core;g=ENSG00000143217) | [PVRL4](http://www.ensembl.org/Homo_sapiens/Gene/Summary?db=core;g=ENSG00000143217) |
| [ENSG00000158793](http://www.ensembl.org/Homo_sapiens/Gene/Summary?db=core;g=ENSG00000158793) | [NIT1](http://www.ensembl.org/Homo_sapiens/Gene/Summary?db=core;g=ENSG00000158793) |
| [ENSG00000197965](http://www.ensembl.org/Homo_sapiens/Gene/Summary?db=core;g=ENSG00000197965) | [MPZL1](http://www.ensembl.org/Homo_sapiens/Gene/Summary?db=core;g=ENSG00000197965) |
| [ENSG00000143158](http://www.ensembl.org/Homo_sapiens/Gene/Summary?db=core;g=ENSG00000143158) | [MPC2](http://www.ensembl.org/Homo_sapiens/Gene/Summary?db=core;g=ENSG00000143158) |
| [ENSG00000158796](http://www.ensembl.org/Homo_sapiens/Gene/Summary?db=core;g=ENSG00000158796) | [DEDD](http://www.ensembl.org/Homo_sapiens/Gene/Summary?db=core;g=ENSG00000158796) |
| [ENSG00000158874](http://www.ensembl.org/Homo_sapiens/Gene/Summary?db=core;g=ENSG00000158874) | [APOA2](http://www.ensembl.org/Homo_sapiens/Gene/Summary?db=core;g=ENSG00000158874) |
| [ENSG00000143224](http://www.ensembl.org/Homo_sapiens/Gene/Summary?db=core;g=ENSG00000143224) | [PPOX](http://www.ensembl.org/Homo_sapiens/Gene/Summary?db=core;g=ENSG00000143224) |
| [ENSG00000122223](http://www.ensembl.org/Homo_sapiens/Gene/Summary?db=core;g=ENSG00000122223) | [CD244](http://www.ensembl.org/Homo_sapiens/Gene/Summary?db=core;g=ENSG00000122223) |
| [ENSG00000158864](http://www.ensembl.org/Homo_sapiens/Gene/Summary?db=core;g=ENSG00000158864) | [NDUFS2](http://www.ensembl.org/Homo_sapiens/Gene/Summary?db=core;g=ENSG00000158864) |
| [ENSG00000186517](http://www.ensembl.org/Homo_sapiens/Gene/Summary?db=core;g=ENSG00000186517) | [ARHGAP30](http://www.ensembl.org/Homo_sapiens/Gene/Summary?db=core;g=ENSG00000186517) |
| [ENSG00000179914](http://www.ensembl.org/Homo_sapiens/Gene/Summary?db=core;g=ENSG00000179914) | [ITLN1](http://www.ensembl.org/Homo_sapiens/Gene/Summary?db=core;g=ENSG00000179914) |
| [ENSG00000158859](http://www.ensembl.org/Homo_sapiens/Gene/Summary?db=core;g=ENSG00000158859) | [ADAMTS4](http://www.ensembl.org/Homo_sapiens/Gene/Summary?db=core;g=ENSG00000158859) |
| [ENSG00000143256](http://www.ensembl.org/Homo_sapiens/Gene/Summary?db=core;g=ENSG00000143256) | [PFDN2](http://www.ensembl.org/Homo_sapiens/Gene/Summary?db=core;g=ENSG00000143256) |
| [ENSG00000143222](http://www.ensembl.org/Homo_sapiens/Gene/Summary?db=core;g=ENSG00000143222) | [UFC1](http://www.ensembl.org/Homo_sapiens/Gene/Summary?db=core;g=ENSG00000143222) |
| [ENSG00000122224](http://www.ensembl.org/Homo_sapiens/Gene/Summary?db=core;g=ENSG00000122224) | [LY9](http://www.ensembl.org/Homo_sapiens/Gene/Summary?db=core;g=ENSG00000122224) |
| [ENSG00000198842](http://www.ensembl.org/Homo_sapiens/Gene/Summary?db=core;g=ENSG00000198842) | [DUSP27](http://www.ensembl.org/Homo_sapiens/Gene/Summary?db=core;g=ENSG00000198842) |
| [ENSG00000158773](http://www.ensembl.org/Homo_sapiens/Gene/Summary?db=core;g=ENSG00000158773) | [USF1](http://www.ensembl.org/Homo_sapiens/Gene/Summary?db=core;g=ENSG00000158773) |
| [ENSG00000143156](http://www.ensembl.org/Homo_sapiens/Gene/Summary?db=core;g=ENSG00000143156) | [NME7](http://www.ensembl.org/Homo_sapiens/Gene/Summary?db=core;g=ENSG00000143156) |
| [ENSG00000162763](http://www.ensembl.org/Homo_sapiens/Gene/Summary?db=core;g=ENSG00000162763) | [LRRC52](http://www.ensembl.org/Homo_sapiens/Gene/Summary?db=core;g=ENSG00000162763) |
| [ENSG00000117091](http://www.ensembl.org/Homo_sapiens/Gene/Summary?db=core;g=ENSG00000117091) | [CD48](http://www.ensembl.org/Homo_sapiens/Gene/Summary?db=core;g=ENSG00000117091) |
| [ENSG00000143167](http://www.ensembl.org/Homo_sapiens/Gene/Summary?db=core;g=ENSG00000143167) | [GPA33](http://www.ensembl.org/Homo_sapiens/Gene/Summary?db=core;g=ENSG00000143167) |
| [ENSG00000143258](http://www.ensembl.org/Homo_sapiens/Gene/Summary?db=core;g=ENSG00000143258) | [USP21](http://www.ensembl.org/Homo_sapiens/Gene/Summary?db=core;g=ENSG00000143258) |
| [ENSG00000198821](http://www.ensembl.org/Homo_sapiens/Gene/Summary?db=core;g=ENSG00000198821) | [CD247](http://www.ensembl.org/Homo_sapiens/Gene/Summary?db=core;g=ENSG00000198821) |
| [ENSG00000075945](http://www.ensembl.org/Homo_sapiens/Gene/Summary?db=core;g=ENSG00000075945) | [KIFAP3](http://www.ensembl.org/Homo_sapiens/Gene/Summary?db=core;g=ENSG00000075945) |
| [ENSG00000117479](http://www.ensembl.org/Homo_sapiens/Gene/Summary?db=core;g=ENSG00000117479) | [SLC19A2](http://www.ensembl.org/Homo_sapiens/Gene/Summary?db=core;g=ENSG00000117479) |
| [ENSG00000026751](http://www.ensembl.org/Homo_sapiens/Gene/Summary?db=core;g=ENSG00000026751) | [SLAMF7](http://www.ensembl.org/Homo_sapiens/Gene/Summary?db=core;g=ENSG00000026751) |
| [ENSG00000143162](http://www.ensembl.org/Homo_sapiens/Gene/Summary?db=core;g=ENSG00000143162) | [CREG1](http://www.ensembl.org/Homo_sapiens/Gene/Summary?db=core;g=ENSG00000143162) |
| [ENSG00000158850](http://www.ensembl.org/Homo_sapiens/Gene/Summary?db=core;g=ENSG00000158850) | [B4GALT3](http://www.ensembl.org/Homo_sapiens/Gene/Summary?db=core;g=ENSG00000158850) |
| [ENSG00000174175](http://www.ensembl.org/Homo_sapiens/Gene/Summary?db=core;g=ENSG00000174175) | [SELP](http://www.ensembl.org/Homo_sapiens/Gene/Summary?db=core;g=ENSG00000174175) |
| [ENSG00000198771](http://www.ensembl.org/Homo_sapiens/Gene/Summary?db=core;g=ENSG00000198771) | [RCSD1](http://www.ensembl.org/Homo_sapiens/Gene/Summary?db=core;g=ENSG00000198771) |
| [ENSG00000143149](http://www.ensembl.org/Homo_sapiens/Gene/Summary?db=core;g=ENSG00000143149) | [ALDH9A1](http://www.ensembl.org/Homo_sapiens/Gene/Summary?db=core;g=ENSG00000143149) |
| [ENSG00000143164](http://www.ensembl.org/Homo_sapiens/Gene/Summary?db=core;g=ENSG00000143164) | [DCAF6](http://www.ensembl.org/Homo_sapiens/Gene/Summary?db=core;g=ENSG00000143164) |
| [ENSG00000143190](http://www.ensembl.org/Homo_sapiens/Gene/Summary?db=core;g=ENSG00000143190) | [POU2F1](http://www.ensembl.org/Homo_sapiens/Gene/Summary?db=core;g=ENSG00000143190) |
| [ENSG00000162761](http://www.ensembl.org/Homo_sapiens/Gene/Summary?db=core;g=ENSG00000162761) | [LMX1A](http://www.ensembl.org/Homo_sapiens/Gene/Summary?db=core;g=ENSG00000162761) |
| [ENSG00000198734](http://www.ensembl.org/Homo_sapiens/Gene/Summary?db=core;g=ENSG00000198734) | [F5](http://www.ensembl.org/Homo_sapiens/Gene/Summary?db=core;g=ENSG00000198734) |
| [ENSG00000143171](http://www.ensembl.org/Homo_sapiens/Gene/Summary?db=core;g=ENSG00000143171) | [RXRG](http://www.ensembl.org/Homo_sapiens/Gene/Summary?db=core;g=ENSG00000143171) |
| [ENSG00000143198](http://www.ensembl.org/Homo_sapiens/Gene/Summary?db=core;g=ENSG00000143198) | [MGST3](http://www.ensembl.org/Homo_sapiens/Gene/Summary?db=core;g=ENSG00000143198) |
| [ENSG00000162739](http://www.ensembl.org/Homo_sapiens/Gene/Summary?db=core;g=ENSG00000162739) | [SLAMF6](http://www.ensembl.org/Homo_sapiens/Gene/Summary?db=core;g=ENSG00000162739) |
| [ENSG00000244682](http://www.ensembl.org/Homo_sapiens/Gene/Summary?db=core;g=ENSG00000244682) | [FCGR2C](http://www.ensembl.org/Homo_sapiens/Gene/Summary?db=core;g=ENSG00000244682) |
| [ENSG00000173110](http://www.ensembl.org/Homo_sapiens/Gene/Summary?db=core;g=ENSG00000173110) | [HSPA6](http://www.ensembl.org/Homo_sapiens/Gene/Summary?db=core;g=ENSG00000173110) |
| [ENSG00000143226](http://www.ensembl.org/Homo_sapiens/Gene/Summary?db=core;g=ENSG00000143226) | [FCGR2A](http://www.ensembl.org/Homo_sapiens/Gene/Summary?db=core;g=ENSG00000143226) |
| [ENSG00000203747](http://www.ensembl.org/Homo_sapiens/Gene/Summary?db=core;g=ENSG00000203747) | [FCGR3A](http://www.ensembl.org/Homo_sapiens/Gene/Summary?db=core;g=ENSG00000203747) |
| [ENSG00000072694](http://www.ensembl.org/Homo_sapiens/Gene/Summary?db=core;g=ENSG00000072694) | [FCGR2B](http://www.ensembl.org/Homo_sapiens/Gene/Summary?db=core;g=ENSG00000072694) |
| [ENSG00000143153](http://www.ensembl.org/Homo_sapiens/Gene/Summary?db=core;g=ENSG00000143153) | [ATP1B1](http://www.ensembl.org/Homo_sapiens/Gene/Summary?db=core;g=ENSG00000143153) |
| [ENSG00000066294](http://www.ensembl.org/Homo_sapiens/Gene/Summary?db=core;g=ENSG00000066294) | [CD84](http://www.ensembl.org/Homo_sapiens/Gene/Summary?db=core;g=ENSG00000066294) |
| [ENSG00000171806](http://www.ensembl.org/Homo_sapiens/Gene/Summary?db=core;g=ENSG00000171806) | [METTL18](http://www.ensembl.org/Homo_sapiens/Gene/Summary?db=core;g=ENSG00000171806) |
| [ENSG00000143179](http://www.ensembl.org/Homo_sapiens/Gene/Summary?db=core;g=ENSG00000143179) | [UCK2](http://www.ensembl.org/Homo_sapiens/Gene/Summary?db=core;g=ENSG00000143179) |
| [ENSG00000000457](http://www.ensembl.org/Homo_sapiens/Gene/Summary?db=core;g=ENSG00000000457) | [SCYL3](http://www.ensembl.org/Homo_sapiens/Gene/Summary?db=core;g=ENSG00000000457) |
| [ENSG00000117477](http://www.ensembl.org/Homo_sapiens/Gene/Summary?db=core;g=ENSG00000117477) | [CCDC181](http://www.ensembl.org/Homo_sapiens/Gene/Summary?db=core;g=ENSG00000117477) |
| [ENSG00000188404](http://www.ensembl.org/Homo_sapiens/Gene/Summary?db=core;g=ENSG00000188404) | [SELL](http://www.ensembl.org/Homo_sapiens/Gene/Summary?db=core;g=ENSG00000188404) |
| [ENSG00000143195](http://www.ensembl.org/Homo_sapiens/Gene/Summary?db=core;g=ENSG00000143195) | [ILDR2](http://www.ensembl.org/Homo_sapiens/Gene/Summary?db=core;g=ENSG00000143195) |
| [ENSG00000143199](http://www.ensembl.org/Homo_sapiens/Gene/Summary?db=core;g=ENSG00000143199) | [ADCY10](http://www.ensembl.org/Homo_sapiens/Gene/Summary?db=core;g=ENSG00000143199) |
| [ENSG00000213064](http://www.ensembl.org/Homo_sapiens/Gene/Summary?db=core;g=ENSG00000213064) | [SFT2D2](http://www.ensembl.org/Homo_sapiens/Gene/Summary?db=core;g=ENSG00000213064) |
| [ENSG00000158769](http://www.ensembl.org/Homo_sapiens/Gene/Summary?db=core;g=ENSG00000158769) | [F11R](http://www.ensembl.org/Homo_sapiens/Gene/Summary?db=core;g=ENSG00000158769) |
| [ENSG00000162747](http://www.ensembl.org/Homo_sapiens/Gene/Summary?db=core;g=ENSG00000162747) | [FCGR3B](http://www.ensembl.org/Homo_sapiens/Gene/Summary?db=core;g=ENSG00000162747) |
| [ENSG00000215845](http://www.ensembl.org/Homo_sapiens/Gene/Summary?db=core;g=ENSG00000215845) | [TSTD1](http://www.ensembl.org/Homo_sapiens/Gene/Summary?db=core;g=ENSG00000215845) |
| [ENSG00000143178](http://www.ensembl.org/Homo_sapiens/Gene/Summary?db=core;g=ENSG00000143178) | [TBX19](http://www.ensembl.org/Homo_sapiens/Gene/Summary?db=core;g=ENSG00000143178) |
| [ENSG00000132185](http://www.ensembl.org/Homo_sapiens/Gene/Summary?db=core;g=ENSG00000132185) | [FCRLA](http://www.ensembl.org/Homo_sapiens/Gene/Summary?db=core;g=ENSG00000132185) |
| [ENSG00000117090](http://www.ensembl.org/Homo_sapiens/Gene/Summary?db=core;g=ENSG00000117090) | [SLAMF1](http://www.ensembl.org/Homo_sapiens/Gene/Summary?db=core;g=ENSG00000117090) |
| [ENSG00000162746](http://www.ensembl.org/Homo_sapiens/Gene/Summary?db=core;g=ENSG00000162746) | [FCRLB](http://www.ensembl.org/Homo_sapiens/Gene/Summary?db=core;g=ENSG00000162746) |
| [ENSG00000081721](http://www.ensembl.org/Homo_sapiens/Gene/Summary?db=core;g=ENSG00000081721) | [DUSP12](http://www.ensembl.org/Homo_sapiens/Gene/Summary?db=core;g=ENSG00000081721) |
| [ENSG00000118217](http://www.ensembl.org/Homo_sapiens/Gene/Summary?db=core;g=ENSG00000118217) | [ATF6](http://www.ensembl.org/Homo_sapiens/Gene/Summary?db=core;g=ENSG00000118217) |
| [ENSG00000162745](http://www.ensembl.org/Homo_sapiens/Gene/Summary?db=core;g=ENSG00000162745) | [OLFML2B](http://www.ensembl.org/Homo_sapiens/Gene/Summary?db=core;g=ENSG00000162745) |
| [ENSG00000198929](http://www.ensembl.org/Homo_sapiens/Gene/Summary?db=core;g=ENSG00000198929) | [NOS1AP](http://www.ensembl.org/Homo_sapiens/Gene/Summary?db=core;g=ENSG00000198929) |
| [ENSG00000171722](http://www.ensembl.org/Homo_sapiens/Gene/Summary?db=core;g=ENSG00000171722) | [C1orf111](http://www.ensembl.org/Homo_sapiens/Gene/Summary?db=core;g=ENSG00000171722) |
| [ENSG00000143194](http://www.ensembl.org/Homo_sapiens/Gene/Summary?db=core;g=ENSG00000143194) | [MAEL](http://www.ensembl.org/Homo_sapiens/Gene/Summary?db=core;g=ENSG00000143194) |
| [ENSG00000185630](http://www.ensembl.org/Homo_sapiens/Gene/Summary?db=core;g=ENSG00000185630) | [PBX1](http://www.ensembl.org/Homo_sapiens/Gene/Summary?db=core;g=ENSG00000185630) |
| [ENSG00000007908](http://www.ensembl.org/Homo_sapiens/Gene/Summary?db=core;g=ENSG00000007908) | [SELE](http://www.ensembl.org/Homo_sapiens/Gene/Summary?db=core;g=ENSG00000007908) |
| [ENSG00000198574](http://www.ensembl.org/Homo_sapiens/Gene/Summary?db=core;g=ENSG00000198574) | [SH2D1B](http://www.ensembl.org/Homo_sapiens/Gene/Summary?db=core;g=ENSG00000198574) |
| [ENSG00000152332](http://www.ensembl.org/Homo_sapiens/Gene/Summary?db=core;g=ENSG00000152332) | [UHMK1](http://www.ensembl.org/Homo_sapiens/Gene/Summary?db=core;g=ENSG00000152332) |
| [ENSG00000117143](http://www.ensembl.org/Homo_sapiens/Gene/Summary?db=core;g=ENSG00000117143) | [UAP1](http://www.ensembl.org/Homo_sapiens/Gene/Summary?db=core;g=ENSG00000117143) |
| [ENSG00000162733](http://www.ensembl.org/Homo_sapiens/Gene/Summary?db=core;g=ENSG00000162733) | [DDR2](http://www.ensembl.org/Homo_sapiens/Gene/Summary?db=core;g=ENSG00000162733) |
| [ENSG00000132196](http://www.ensembl.org/Homo_sapiens/Gene/Summary?db=core;g=ENSG00000132196) | [HSD17B7](http://www.ensembl.org/Homo_sapiens/Gene/Summary?db=core;g=ENSG00000132196) |
| [ENSG00000117152](http://www.ensembl.org/Homo_sapiens/Gene/Summary?db=core;g=ENSG00000117152) | [RGS4](http://www.ensembl.org/Homo_sapiens/Gene/Summary?db=core;g=ENSG00000117152) |
| [ENSG00000143228](http://www.ensembl.org/Homo_sapiens/Gene/Summary?db=core;g=ENSG00000143228) | [NUF2](http://www.ensembl.org/Homo_sapiens/Gene/Summary?db=core;g=ENSG00000143228) |
| [ENSG00000143248](http://www.ensembl.org/Homo_sapiens/Gene/Summary?db=core;g=ENSG00000143248) | [RGS5](http://www.ensembl.org/Homo_sapiens/Gene/Summary?db=core;g=ENSG00000143248) |
| [ENSG00000143185](http://www.ensembl.org/Homo_sapiens/Gene/Summary?db=core;g=ENSG00000143185) | [XCL2](http://www.ensembl.org/Homo_sapiens/Gene/Summary?db=core;g=ENSG00000143185) |
| [ENSG00000143184](http://www.ensembl.org/Homo_sapiens/Gene/Summary?db=core;g=ENSG00000143184) | [XCL1](http://www.ensembl.org/Homo_sapiens/Gene/Summary?db=core;g=ENSG00000143184) |
| [ENSG00000143196](http://www.ensembl.org/Homo_sapiens/Gene/Summary?db=core;g=ENSG00000143196) | [DPT](http://www.ensembl.org/Homo_sapiens/Gene/Summary?db=core;g=ENSG00000143196) |
| [ENSG00000158764](http://www.ensembl.org/Homo_sapiens/Gene/Summary?db=core;g=ENSG00000158764) | [ITLN2](http://www.ensembl.org/Homo_sapiens/Gene/Summary?db=core;g=ENSG00000158764) |
| [ENSG00000143155](http://www.ensembl.org/Homo_sapiens/Gene/Summary?db=core;g=ENSG00000143155) | [TIPRL](http://www.ensembl.org/Homo_sapiens/Gene/Summary?db=core;g=ENSG00000143155) |
| [ENSG00000143257](http://www.ensembl.org/Homo_sapiens/Gene/Summary?db=core;g=ENSG00000143257) | [NR1I3](http://www.ensembl.org/Homo_sapiens/Gene/Summary?db=core;g=ENSG00000143257) |
| [ENSG00000158882](http://www.ensembl.org/Homo_sapiens/Gene/Summary?db=core;g=ENSG00000158882) | [TOMM40L](http://www.ensembl.org/Homo_sapiens/Gene/Summary?db=core;g=ENSG00000158882) |
| [ENSG00000143157](http://www.ensembl.org/Homo_sapiens/Gene/Summary?db=core;g=ENSG00000143157) | [POGK](http://www.ensembl.org/Homo_sapiens/Gene/Summary?db=core;g=ENSG00000143157) |
| [ENSG00000152382](http://www.ensembl.org/Homo_sapiens/Gene/Summary?db=core;g=ENSG00000152382) | [TADA1](http://www.ensembl.org/Homo_sapiens/Gene/Summary?db=core;g=ENSG00000152382) |
| [ENSG00000158887](http://www.ensembl.org/Homo_sapiens/Gene/Summary?db=core;g=ENSG00000158887) | [MPZ](http://www.ensembl.org/Homo_sapiens/Gene/Summary?db=core;g=ENSG00000158887) |
| [ENSG00000188931](http://www.ensembl.org/Homo_sapiens/Gene/Summary?db=core;g=ENSG00000188931) | [CFAP126](http://www.ensembl.org/Homo_sapiens/Gene/Summary?db=core;g=ENSG00000188931) |
| [ENSG00000143252](http://www.ensembl.org/Homo_sapiens/Gene/Summary?db=core;g=ENSG00000143252) | [SDHC](http://www.ensembl.org/Homo_sapiens/Gene/Summary?db=core;g=ENSG00000143252) |
| [ENSG00000140941](http://www.ensembl.org/Homo_sapiens/Gene/Summary?db=core;g=ENSG00000140941) | [MAP1LC3B](http://www.ensembl.org/Homo_sapiens/Gene/Summary?db=core;g=ENSG00000140941) |
| [ENSG00000153815](http://www.ensembl.org/Homo_sapiens/Gene/Summary?db=core;g=ENSG00000153815) | [CMIP](http://www.ensembl.org/Homo_sapiens/Gene/Summary?db=core;g=ENSG00000153815) |
| [ENSG00000064270](http://www.ensembl.org/Homo_sapiens/Gene/Summary?db=core;g=ENSG00000064270) | [ATP2C2](http://www.ensembl.org/Homo_sapiens/Gene/Summary?db=core;g=ENSG00000064270) |
| [ENSG00000166509](http://www.ensembl.org/Homo_sapiens/Gene/Summary?db=core;g=ENSG00000166509) | [CLEC3A](http://www.ensembl.org/Homo_sapiens/Gene/Summary?db=core;g=ENSG00000166509) |
| [ENSG00000140950](http://www.ensembl.org/Homo_sapiens/Gene/Summary?db=core;g=ENSG00000140950) | [TLDC1](http://www.ensembl.org/Homo_sapiens/Gene/Summary?db=core;g=ENSG00000140950) |
| [ENSG00000186153](http://www.ensembl.org/Homo_sapiens/Gene/Summary?db=core;g=ENSG00000186153) | [WWOX](http://www.ensembl.org/Homo_sapiens/Gene/Summary?db=core;g=ENSG00000186153) |
| [ENSG00000103150](http://www.ensembl.org/Homo_sapiens/Gene/Summary?db=core;g=ENSG00000103150) | [MLYCD](http://www.ensembl.org/Homo_sapiens/Gene/Summary?db=core;g=ENSG00000103150) |
| [ENSG00000171724](http://www.ensembl.org/Homo_sapiens/Gene/Summary?db=core;g=ENSG00000171724) | [VAT1L](http://www.ensembl.org/Homo_sapiens/Gene/Summary?db=core;g=ENSG00000171724) |
| [ENSG00000184860](http://www.ensembl.org/Homo_sapiens/Gene/Summary?db=core;g=ENSG00000184860) | [SDR42E1](http://www.ensembl.org/Homo_sapiens/Gene/Summary?db=core;g=ENSG00000184860) |
| [ENSG00000260300](http://www.ensembl.org/Homo_sapiens/Gene/Summary?db=core;g=ENSG00000260300) | [RP11-505K9.4](http://www.ensembl.org/Homo_sapiens/Gene/Summary?db=core;g=ENSG00000260300) |
| [ENSG00000140961](http://www.ensembl.org/Homo_sapiens/Gene/Summary?db=core;g=ENSG00000140961) | [OSGIN1](http://www.ensembl.org/Homo_sapiens/Gene/Summary?db=core;g=ENSG00000140961) |
| [ENSG00000131153](http://www.ensembl.org/Homo_sapiens/Gene/Summary?db=core;g=ENSG00000131153) | [GINS2](http://www.ensembl.org/Homo_sapiens/Gene/Summary?db=core;g=ENSG00000131153) |
| [ENSG00000154099](http://www.ensembl.org/Homo_sapiens/Gene/Summary?db=core;g=ENSG00000154099) | [DNAAF1](http://www.ensembl.org/Homo_sapiens/Gene/Summary?db=core;g=ENSG00000154099) |
| [ENSG00000103175](http://www.ensembl.org/Homo_sapiens/Gene/Summary?db=core;g=ENSG00000103175) | [WFDC1](http://www.ensembl.org/Homo_sapiens/Gene/Summary?db=core;g=ENSG00000103175) |
| [ENSG00000103196](http://www.ensembl.org/Homo_sapiens/Gene/Summary?db=core;g=ENSG00000103196) | [CRISPLD2](http://www.ensembl.org/Homo_sapiens/Gene/Summary?db=core;g=ENSG00000103196) |
| [ENSG00000176692](http://www.ensembl.org/Homo_sapiens/Gene/Summary?db=core;g=ENSG00000176692) | [FOXC2](http://www.ensembl.org/Homo_sapiens/Gene/Summary?db=core;g=ENSG00000176692) |
| [ENSG00000166454](http://www.ensembl.org/Homo_sapiens/Gene/Summary?db=core;g=ENSG00000166454) | [ATMIN](http://www.ensembl.org/Homo_sapiens/Gene/Summary?db=core;g=ENSG00000166454) |
| [ENSG00000176678](http://www.ensembl.org/Homo_sapiens/Gene/Summary?db=core;g=ENSG00000176678) | [FOXL1](http://www.ensembl.org/Homo_sapiens/Gene/Summary?db=core;g=ENSG00000176678) |
| [ENSG00000154118](http://www.ensembl.org/Homo_sapiens/Gene/Summary?db=core;g=ENSG00000154118) | [JPH3](http://www.ensembl.org/Homo_sapiens/Gene/Summary?db=core;g=ENSG00000154118) |
| [ENSG00000103168](http://www.ensembl.org/Homo_sapiens/Gene/Summary?db=core;g=ENSG00000103168) | [TAF1C](http://www.ensembl.org/Homo_sapiens/Gene/Summary?db=core;g=ENSG00000103168) |
| [ENSG00000168418](http://www.ensembl.org/Homo_sapiens/Gene/Summary?db=core;g=ENSG00000168418) | [KCNG4](http://www.ensembl.org/Homo_sapiens/Gene/Summary?db=core;g=ENSG00000168418) |
| [ENSG00000166473](http://www.ensembl.org/Homo_sapiens/Gene/Summary?db=core;g=ENSG00000166473) | [PKD1L2](http://www.ensembl.org/Homo_sapiens/Gene/Summary?db=core;g=ENSG00000166473) |
| [ENSG00000230989](http://www.ensembl.org/Homo_sapiens/Gene/Summary?db=core;g=ENSG00000230989) | [HSBP1](http://www.ensembl.org/Homo_sapiens/Gene/Summary?db=core;g=ENSG00000230989) |
| [ENSG00000131149](http://www.ensembl.org/Homo_sapiens/Gene/Summary?db=core;g=ENSG00000131149) | [GSE1](http://www.ensembl.org/Homo_sapiens/Gene/Summary?db=core;g=ENSG00000131149) |
| [ENSG00000140955](http://www.ensembl.org/Homo_sapiens/Gene/Summary?db=core;g=ENSG00000140955) | [ADAD2](http://www.ensembl.org/Homo_sapiens/Gene/Summary?db=core;g=ENSG00000140955) |
| [ENSG00000103194](http://www.ensembl.org/Homo_sapiens/Gene/Summary?db=core;g=ENSG00000103194) | [USP10](http://www.ensembl.org/Homo_sapiens/Gene/Summary?db=core;g=ENSG00000103194) |
| [ENSG00000103121](http://www.ensembl.org/Homo_sapiens/Gene/Summary?db=core;g=ENSG00000103121) | [CMC2](http://www.ensembl.org/Homo_sapiens/Gene/Summary?db=core;g=ENSG00000103121) |
| [ENSG00000166558](http://www.ensembl.org/Homo_sapiens/Gene/Summary?db=core;g=ENSG00000166558) | [SLC38A8](http://www.ensembl.org/Homo_sapiens/Gene/Summary?db=core;g=ENSG00000166558) |
| [ENSG00000103241](http://www.ensembl.org/Homo_sapiens/Gene/Summary?db=core;g=ENSG00000103241) | [FOXF1](http://www.ensembl.org/Homo_sapiens/Gene/Summary?db=core;g=ENSG00000103241) |
| [ENSG00000104731](http://www.ensembl.org/Homo_sapiens/Gene/Summary?db=core;g=ENSG00000104731) | [KLHDC4](http://www.ensembl.org/Homo_sapiens/Gene/Summary?db=core;g=ENSG00000104731) |
| [ENSG00000086696](http://www.ensembl.org/Homo_sapiens/Gene/Summary?db=core;g=ENSG00000086696) | [HSD17B2](http://www.ensembl.org/Homo_sapiens/Gene/Summary?db=core;g=ENSG00000086696) |
| [ENSG00000140948](http://www.ensembl.org/Homo_sapiens/Gene/Summary?db=core;g=ENSG00000140948) | [ZCCHC14](http://www.ensembl.org/Homo_sapiens/Gene/Summary?db=core;g=ENSG00000140948) |
| [ENSG00000103248](http://www.ensembl.org/Homo_sapiens/Gene/Summary?db=core;g=ENSG00000103248) | [MTHFSD](http://www.ensembl.org/Homo_sapiens/Gene/Summary?db=core;g=ENSG00000103248) |
| [ENSG00000135698](http://www.ensembl.org/Homo_sapiens/Gene/Summary?db=core;g=ENSG00000135698) | [MPHOSPH6](http://www.ensembl.org/Homo_sapiens/Gene/Summary?db=core;g=ENSG00000135698) |
| [ENSG00000166451](http://www.ensembl.org/Homo_sapiens/Gene/Summary?db=core;g=ENSG00000166451) | [CENPN](http://www.ensembl.org/Homo_sapiens/Gene/Summary?db=core;g=ENSG00000166451) |
| [ENSG00000166446](http://www.ensembl.org/Homo_sapiens/Gene/Summary?db=core;g=ENSG00000166446) | [CDYL2](http://www.ensembl.org/Homo_sapiens/Gene/Summary?db=core;g=ENSG00000166446) |
| [ENSG00000103187](http://www.ensembl.org/Homo_sapiens/Gene/Summary?db=core;g=ENSG00000103187) | [COTL1](http://www.ensembl.org/Homo_sapiens/Gene/Summary?db=core;g=ENSG00000103187) |
| [ENSG00000135686](http://www.ensembl.org/Homo_sapiens/Gene/Summary?db=core;g=ENSG00000135686) | [KLHL36](http://www.ensembl.org/Homo_sapiens/Gene/Summary?db=core;g=ENSG00000135686) |
| [ENSG00000140943](http://www.ensembl.org/Homo_sapiens/Gene/Summary?db=core;g=ENSG00000140943) | [MBTPS1](http://www.ensembl.org/Homo_sapiens/Gene/Summary?db=core;g=ENSG00000140943) |
| [ENSG00000178573](http://www.ensembl.org/Homo_sapiens/Gene/Summary?db=core;g=ENSG00000178573) | [MAF](http://www.ensembl.org/Homo_sapiens/Gene/Summary?db=core;g=ENSG00000178573) |
| [ENSG00000140968](http://www.ensembl.org/Homo_sapiens/Gene/Summary?db=core;g=ENSG00000140968) | [IRF8](http://www.ensembl.org/Homo_sapiens/Gene/Summary?db=core;g=ENSG00000140968) |
| [ENSG00000135697](http://www.ensembl.org/Homo_sapiens/Gene/Summary?db=core;g=ENSG00000135697) | [BCO1](http://www.ensembl.org/Homo_sapiens/Gene/Summary?db=core;g=ENSG00000135697) |
| [ENSG00000154102](http://www.ensembl.org/Homo_sapiens/Gene/Summary?db=core;g=ENSG00000154102) | [C16orf74](http://www.ensembl.org/Homo_sapiens/Gene/Summary?db=core;g=ENSG00000154102) |
| [ENSG00000153786](http://www.ensembl.org/Homo_sapiens/Gene/Summary?db=core;g=ENSG00000153786) | [ZDHHC7](http://www.ensembl.org/Homo_sapiens/Gene/Summary?db=core;g=ENSG00000153786) |
| [ENSG00000261609](http://www.ensembl.org/Homo_sapiens/Gene/Summary?db=core;g=ENSG00000261609) | [GAN](http://www.ensembl.org/Homo_sapiens/Gene/Summary?db=core;g=ENSG00000261609) |
| [ENSG00000140945](http://www.ensembl.org/Homo_sapiens/Gene/Summary?db=core;g=ENSG00000140945) | [CDH13](http://www.ensembl.org/Homo_sapiens/Gene/Summary?db=core;g=ENSG00000140945) |
| [ENSG00000140905](http://www.ensembl.org/Homo_sapiens/Gene/Summary?db=core;g=ENSG00000140905) | [GCSH](http://www.ensembl.org/Homo_sapiens/Gene/Summary?db=core;g=ENSG00000140905) |
| [ENSG00000135709](http://www.ensembl.org/Homo_sapiens/Gene/Summary?db=core;g=ENSG00000135709) | [KIAA0513](http://www.ensembl.org/Homo_sapiens/Gene/Summary?db=core;g=ENSG00000135709) |
| [ENSG00000131148](http://www.ensembl.org/Homo_sapiens/Gene/Summary?db=core;g=ENSG00000131148) | [EMC8](http://www.ensembl.org/Homo_sapiens/Gene/Summary?db=core;g=ENSG00000131148) |
| [ENSG00000197943](http://www.ensembl.org/Homo_sapiens/Gene/Summary?db=core;g=ENSG00000197943) | [PLCG2](http://www.ensembl.org/Homo_sapiens/Gene/Summary?db=core;g=ENSG00000197943) |
| [ENSG00000166455](http://www.ensembl.org/Homo_sapiens/Gene/Summary?db=core;g=ENSG00000166455) | [C16orf46](http://www.ensembl.org/Homo_sapiens/Gene/Summary?db=core;g=ENSG00000166455) |
| [ENSG00000103154](http://www.ensembl.org/Homo_sapiens/Gene/Summary?db=core;g=ENSG00000103154) | [NECAB2](http://www.ensembl.org/Homo_sapiens/Gene/Summary?db=core;g=ENSG00000103154) |
| [ENSG00000103160](http://www.ensembl.org/Homo_sapiens/Gene/Summary?db=core;g=ENSG00000103160) | [HSDL1](http://www.ensembl.org/Homo_sapiens/Gene/Summary?db=core;g=ENSG00000103160) |
| [ENSG00000131143](http://www.ensembl.org/Homo_sapiens/Gene/Summary?db=core;g=ENSG00000131143) | [COX4I1](http://www.ensembl.org/Homo_sapiens/Gene/Summary?db=core;g=ENSG00000131143) |
| [ENSG00000168589](http://www.ensembl.org/Homo_sapiens/Gene/Summary?db=core;g=ENSG00000168589) | [DYNLRB2](http://www.ensembl.org/Homo_sapiens/Gene/Summary?db=core;g=ENSG00000168589) |
| [ENSG00000103264](http://www.ensembl.org/Homo_sapiens/Gene/Summary?db=core;g=ENSG00000103264) | [FBXO31](http://www.ensembl.org/Homo_sapiens/Gene/Summary?db=core;g=ENSG00000103264) |
| [ENSG00000109079](http://www.ensembl.org/Homo_sapiens/Gene/Summary?db=core;g=ENSG00000109079) | [TNFAIP1](http://www.ensembl.org/Homo_sapiens/Gene/Summary?db=core;g=ENSG00000109079) |
| [ENSG00000132591](http://www.ensembl.org/Homo_sapiens/Gene/Summary?db=core;g=ENSG00000132591) | [ERAL1](http://www.ensembl.org/Homo_sapiens/Gene/Summary?db=core;g=ENSG00000132591) |
| [ENSG00000126861](http://www.ensembl.org/Homo_sapiens/Gene/Summary?db=core;g=ENSG00000126861) | [OMG](http://www.ensembl.org/Homo_sapiens/Gene/Summary?db=core;g=ENSG00000126861) |
| [ENSG00000108733](http://www.ensembl.org/Homo_sapiens/Gene/Summary?db=core;g=ENSG00000108733) | [PEX12](http://www.ensembl.org/Homo_sapiens/Gene/Summary?db=core;g=ENSG00000108733) |
| [ENSG00000108671](http://www.ensembl.org/Homo_sapiens/Gene/Summary?db=core;g=ENSG00000108671) | [PSMD11](http://www.ensembl.org/Homo_sapiens/Gene/Summary?db=core;g=ENSG00000108671) |
| [ENSG00000126860](http://www.ensembl.org/Homo_sapiens/Gene/Summary?db=core;g=ENSG00000126860) | [EVI2A](http://www.ensembl.org/Homo_sapiens/Gene/Summary?db=core;g=ENSG00000126860) |
| [ENSG00000265118](http://www.ensembl.org/Homo_sapiens/Gene/Summary?db=core;g=ENSG00000265118) | [CTD-2370N5.3](http://www.ensembl.org/Homo_sapiens/Gene/Summary?db=core;g=ENSG00000265118) |
| [ENSG00000132589](http://www.ensembl.org/Homo_sapiens/Gene/Summary?db=core;g=ENSG00000132589) | [FLOT2](http://www.ensembl.org/Homo_sapiens/Gene/Summary?db=core;g=ENSG00000132589) |
| [ENSG00000167543](http://www.ensembl.org/Homo_sapiens/Gene/Summary?db=core;g=ENSG00000167543) | [TP53I13](http://www.ensembl.org/Homo_sapiens/Gene/Summary?db=core;g=ENSG00000167543) |
| [ENSG00000179761](http://www.ensembl.org/Homo_sapiens/Gene/Summary?db=core;g=ENSG00000179761) | [PIPOX](http://www.ensembl.org/Homo_sapiens/Gene/Summary?db=core;g=ENSG00000179761) |
| [ENSG00000108578](http://www.ensembl.org/Homo_sapiens/Gene/Summary?db=core;g=ENSG00000108578) | [BLMH](http://www.ensembl.org/Homo_sapiens/Gene/Summary?db=core;g=ENSG00000108578) |
| [ENSG00000109046](http://www.ensembl.org/Homo_sapiens/Gene/Summary?db=core;g=ENSG00000109046) | [WSB1](http://www.ensembl.org/Homo_sapiens/Gene/Summary?db=core;g=ENSG00000109046) |
| [ENSG00000126858](http://www.ensembl.org/Homo_sapiens/Gene/Summary?db=core;g=ENSG00000126858) | [RHOT1](http://www.ensembl.org/Homo_sapiens/Gene/Summary?db=core;g=ENSG00000126858) |
| [ENSG00000185862](http://www.ensembl.org/Homo_sapiens/Gene/Summary?db=core;g=ENSG00000185862) | [EVI2B](http://www.ensembl.org/Homo_sapiens/Gene/Summary?db=core;g=ENSG00000185862) |
| [ENSG00000006042](http://www.ensembl.org/Homo_sapiens/Gene/Summary?db=core;g=ENSG00000006042) | [TMEM98](http://www.ensembl.org/Homo_sapiens/Gene/Summary?db=core;g=ENSG00000006042) |
| [ENSG00000108576](http://www.ensembl.org/Homo_sapiens/Gene/Summary?db=core;g=ENSG00000108576) | [SLC6A4](http://www.ensembl.org/Homo_sapiens/Gene/Summary?db=core;g=ENSG00000108576) |
| [ENSG00000176749](http://www.ensembl.org/Homo_sapiens/Gene/Summary?db=core;g=ENSG00000176749) | [CDK5R1](http://www.ensembl.org/Homo_sapiens/Gene/Summary?db=core;g=ENSG00000176749) |
| [ENSG00000267618](http://www.ensembl.org/Homo_sapiens/Gene/Summary?db=core;g=ENSG00000267618) | [RAD51L3-RFFL](http://www.ensembl.org/Homo_sapiens/Gene/Summary?db=core;g=ENSG00000267618) |
| [ENSG00000176390](http://www.ensembl.org/Homo_sapiens/Gene/Summary?db=core;g=ENSG00000176390) | [CRLF3](http://www.ensembl.org/Homo_sapiens/Gene/Summary?db=core;g=ENSG00000176390) |
| [ENSG00000109072](http://www.ensembl.org/Homo_sapiens/Gene/Summary?db=core;g=ENSG00000109072) | [VTN](http://www.ensembl.org/Homo_sapiens/Gene/Summary?db=core;g=ENSG00000109072) |
| [ENSG00000244045](http://www.ensembl.org/Homo_sapiens/Gene/Summary?db=core;g=ENSG00000244045) | [TMEM199](http://www.ensembl.org/Homo_sapiens/Gene/Summary?db=core;g=ENSG00000244045) |
| [ENSG00000126653](http://www.ensembl.org/Homo_sapiens/Gene/Summary?db=core;g=ENSG00000126653) | [NSRP1](http://www.ensembl.org/Homo_sapiens/Gene/Summary?db=core;g=ENSG00000126653) |
| [ENSG00000087111](http://www.ensembl.org/Homo_sapiens/Gene/Summary?db=core;g=ENSG00000087111) | [PIGS](http://www.ensembl.org/Homo_sapiens/Gene/Summary?db=core;g=ENSG00000087111) |
| [ENSG00000087095](http://www.ensembl.org/Homo_sapiens/Gene/Summary?db=core;g=ENSG00000087095) | [NLK](http://www.ensembl.org/Homo_sapiens/Gene/Summary?db=core;g=ENSG00000087095) |
| [ENSG00000108702](http://www.ensembl.org/Homo_sapiens/Gene/Summary?db=core;g=ENSG00000108702) | [CCL1](http://www.ensembl.org/Homo_sapiens/Gene/Summary?db=core;g=ENSG00000108702) |
| [ENSG00000168961](http://www.ensembl.org/Homo_sapiens/Gene/Summary?db=core;g=ENSG00000168961) | [LGALS9](http://www.ensembl.org/Homo_sapiens/Gene/Summary?db=core;g=ENSG00000168961) |
| [ENSG00000076351](http://www.ensembl.org/Homo_sapiens/Gene/Summary?db=core;g=ENSG00000076351) | [SLC46A1](http://www.ensembl.org/Homo_sapiens/Gene/Summary?db=core;g=ENSG00000076351) |
| [ENSG00000181291](http://www.ensembl.org/Homo_sapiens/Gene/Summary?db=core;g=ENSG00000181291) | [TMEM132E](http://www.ensembl.org/Homo_sapiens/Gene/Summary?db=core;g=ENSG00000181291) |
| [ENSG00000173065](http://www.ensembl.org/Homo_sapiens/Gene/Summary?db=core;g=ENSG00000173065) | [FAM222B](http://www.ensembl.org/Homo_sapiens/Gene/Summary?db=core;g=ENSG00000173065) |
| [ENSG00000196535](http://www.ensembl.org/Homo_sapiens/Gene/Summary?db=core;g=ENSG00000196535) | [MYO18A](http://www.ensembl.org/Homo_sapiens/Gene/Summary?db=core;g=ENSG00000196535) |
| [ENSG00000205045](http://www.ensembl.org/Homo_sapiens/Gene/Summary?db=core;g=ENSG00000205045) | [SLFN12L](http://www.ensembl.org/Homo_sapiens/Gene/Summary?db=core;g=ENSG00000205045) |
| [ENSG00000132141](http://www.ensembl.org/Homo_sapiens/Gene/Summary?db=core;g=ENSG00000132141) | [CCT6B](http://www.ensembl.org/Homo_sapiens/Gene/Summary?db=core;g=ENSG00000132141) |
| [ENSG00000108691](http://www.ensembl.org/Homo_sapiens/Gene/Summary?db=core;g=ENSG00000108691) | [CCL2](http://www.ensembl.org/Homo_sapiens/Gene/Summary?db=core;g=ENSG00000108691) |
| [ENSG00000076604](http://www.ensembl.org/Homo_sapiens/Gene/Summary?db=core;g=ENSG00000076604) | [TRAF4](http://www.ensembl.org/Homo_sapiens/Gene/Summary?db=core;g=ENSG00000076604) |
| [ENSG00000109101](http://www.ensembl.org/Homo_sapiens/Gene/Summary?db=core;g=ENSG00000109101) | [FOXN1](http://www.ensembl.org/Homo_sapiens/Gene/Summary?db=core;g=ENSG00000109101) |
| [ENSG00000198783](http://www.ensembl.org/Homo_sapiens/Gene/Summary?db=core;g=ENSG00000198783) | [ZNF830](http://www.ensembl.org/Homo_sapiens/Gene/Summary?db=core;g=ENSG00000198783) |
| [ENSG00000131242](http://www.ensembl.org/Homo_sapiens/Gene/Summary?db=core;g=ENSG00000131242) | [RAB11FIP4](http://www.ensembl.org/Homo_sapiens/Gene/Summary?db=core;g=ENSG00000131242) |
| [ENSG00000160551](http://www.ensembl.org/Homo_sapiens/Gene/Summary?db=core;g=ENSG00000160551) | [TAOK1](http://www.ensembl.org/Homo_sapiens/Gene/Summary?db=core;g=ENSG00000160551) |
| [ENSG00000063015](http://www.ensembl.org/Homo_sapiens/Gene/Summary?db=core;g=ENSG00000063015) | [SEZ6](http://www.ensembl.org/Homo_sapiens/Gene/Summary?db=core;g=ENSG00000063015) |
| [ENSG00000006125](http://www.ensembl.org/Homo_sapiens/Gene/Summary?db=core;g=ENSG00000006125) | [AP2B1](http://www.ensembl.org/Homo_sapiens/Gene/Summary?db=core;g=ENSG00000006125) |
| [ENSG00000185379](http://www.ensembl.org/Homo_sapiens/Gene/Summary?db=core;g=ENSG00000185379) | [RAD51D](http://www.ensembl.org/Homo_sapiens/Gene/Summary?db=core;g=ENSG00000185379) |
| [ENSG00000185158](http://www.ensembl.org/Homo_sapiens/Gene/Summary?db=core;g=ENSG00000185158) | [LRRC37B](http://www.ensembl.org/Homo_sapiens/Gene/Summary?db=core;g=ENSG00000185158) |
| [ENSG00000092871](http://www.ensembl.org/Homo_sapiens/Gene/Summary?db=core;g=ENSG00000092871) | [RFFL](http://www.ensembl.org/Homo_sapiens/Gene/Summary?db=core;g=ENSG00000092871) |
| [ENSG00000168792](http://www.ensembl.org/Homo_sapiens/Gene/Summary?db=core;g=ENSG00000168792) | [ABHD15](http://www.ensembl.org/Homo_sapiens/Gene/Summary?db=core;g=ENSG00000168792) |
| [ENSG00000109111](http://www.ensembl.org/Homo_sapiens/Gene/Summary?db=core;g=ENSG00000109111) | [SUPT6H](http://www.ensembl.org/Homo_sapiens/Gene/Summary?db=core;g=ENSG00000109111) |
| [ENSG00000108582](http://www.ensembl.org/Homo_sapiens/Gene/Summary?db=core;g=ENSG00000108582) | [CPD](http://www.ensembl.org/Homo_sapiens/Gene/Summary?db=core;g=ENSG00000108582) |
| [ENSG00000181374](http://www.ensembl.org/Homo_sapiens/Gene/Summary?db=core;g=ENSG00000181374) | [CCL13](http://www.ensembl.org/Homo_sapiens/Gene/Summary?db=core;g=ENSG00000181374) |
| [ENSG00000172716](http://www.ensembl.org/Homo_sapiens/Gene/Summary?db=core;g=ENSG00000172716) | [SLFN11](http://www.ensembl.org/Homo_sapiens/Gene/Summary?db=core;g=ENSG00000172716) |
| [ENSG00000132581](http://www.ensembl.org/Homo_sapiens/Gene/Summary?db=core;g=ENSG00000132581) | [SDF2](http://www.ensembl.org/Homo_sapiens/Gene/Summary?db=core;g=ENSG00000132581) |
| [ENSG00000007202](http://www.ensembl.org/Homo_sapiens/Gene/Summary?db=core;g=ENSG00000007202) | [KIAA0100](http://www.ensembl.org/Homo_sapiens/Gene/Summary?db=core;g=ENSG00000007202) |
| [ENSG00000154760](http://www.ensembl.org/Homo_sapiens/Gene/Summary?db=core;g=ENSG00000154760) | [SLFN13](http://www.ensembl.org/Homo_sapiens/Gene/Summary?db=core;g=ENSG00000154760) |
| [ENSG00000141316](http://www.ensembl.org/Homo_sapiens/Gene/Summary?db=core;g=ENSG00000141316) | [SPACA3](http://www.ensembl.org/Homo_sapiens/Gene/Summary?db=core;g=ENSG00000141316) |
| [ENSG00000109118](http://www.ensembl.org/Homo_sapiens/Gene/Summary?db=core;g=ENSG00000109118) | [PHF12](http://www.ensembl.org/Homo_sapiens/Gene/Summary?db=core;g=ENSG00000109118) |
| [ENSG00000176658](http://www.ensembl.org/Homo_sapiens/Gene/Summary?db=core;g=ENSG00000176658) | [MYO1D](http://www.ensembl.org/Homo_sapiens/Gene/Summary?db=core;g=ENSG00000176658) |
| [ENSG00000004139](http://www.ensembl.org/Homo_sapiens/Gene/Summary?db=core;g=ENSG00000004139) | [SARM1](http://www.ensembl.org/Homo_sapiens/Gene/Summary?db=core;g=ENSG00000004139) |
| [ENSG00000258472](http://www.ensembl.org/Homo_sapiens/Gene/Summary?db=core;g=ENSG00000258472) | [RP11-192H23.4](http://www.ensembl.org/Homo_sapiens/Gene/Summary?db=core;g=ENSG00000258472) |
| [ENSG00000010244](http://www.ensembl.org/Homo_sapiens/Gene/Summary?db=core;g=ENSG00000010244) | [ZNF207](http://www.ensembl.org/Homo_sapiens/Gene/Summary?db=core;g=ENSG00000010244) |
| [ENSG00000007171](http://www.ensembl.org/Homo_sapiens/Gene/Summary?db=core;g=ENSG00000007171) | [NOS2](http://www.ensembl.org/Homo_sapiens/Gene/Summary?db=core;g=ENSG00000007171) |
| [ENSG00000176208](http://www.ensembl.org/Homo_sapiens/Gene/Summary?db=core;g=ENSG00000176208) | [ATAD5](http://www.ensembl.org/Homo_sapiens/Gene/Summary?db=core;g=ENSG00000176208) |
| [ENSG00000109083](http://www.ensembl.org/Homo_sapiens/Gene/Summary?db=core;g=ENSG00000109083) | [IFT20](http://www.ensembl.org/Homo_sapiens/Gene/Summary?db=core;g=ENSG00000109083) |
| [ENSG00000198720](http://www.ensembl.org/Homo_sapiens/Gene/Summary?db=core;g=ENSG00000198720) | [ANKRD13B](http://www.ensembl.org/Homo_sapiens/Gene/Summary?db=core;g=ENSG00000198720) |
| [ENSG00000108255](http://www.ensembl.org/Homo_sapiens/Gene/Summary?db=core;g=ENSG00000108255) | [CRYBA1](http://www.ensembl.org/Homo_sapiens/Gene/Summary?db=core;g=ENSG00000108255) |
| [ENSG00000196712](http://www.ensembl.org/Homo_sapiens/Gene/Summary?db=core;g=ENSG00000196712) | [NF1](http://www.ensembl.org/Homo_sapiens/Gene/Summary?db=core;g=ENSG00000196712) |
| [ENSG00000166750](http://www.ensembl.org/Homo_sapiens/Gene/Summary?db=core;g=ENSG00000166750) | [SLFN5](http://www.ensembl.org/Homo_sapiens/Gene/Summary?db=core;g=ENSG00000166750) |
| [ENSG00000108700](http://www.ensembl.org/Homo_sapiens/Gene/Summary?db=core;g=ENSG00000108700) | [CCL8](http://www.ensembl.org/Homo_sapiens/Gene/Summary?db=core;g=ENSG00000108700) |
| [ENSG00000172123](http://www.ensembl.org/Homo_sapiens/Gene/Summary?db=core;g=ENSG00000172123) | [SLFN12](http://www.ensembl.org/Homo_sapiens/Gene/Summary?db=core;g=ENSG00000172123) |
| [ENSG00000178691](http://www.ensembl.org/Homo_sapiens/Gene/Summary?db=core;g=ENSG00000178691) | [SUZ12](http://www.ensembl.org/Homo_sapiens/Gene/Summary?db=core;g=ENSG00000178691) |
| [ENSG00000160602](http://www.ensembl.org/Homo_sapiens/Gene/Summary?db=core;g=ENSG00000160602) | [NEK8](http://www.ensembl.org/Homo_sapiens/Gene/Summary?db=core;g=ENSG00000160602) |
| [ENSG00000274529](http://www.ensembl.org/Homo_sapiens/Gene/Summary?db=core;g=ENSG00000274529) | [SEBOX](http://www.ensembl.org/Homo_sapiens/Gene/Summary?db=core;g=ENSG00000274529) |
| [ENSG00000004142](http://www.ensembl.org/Homo_sapiens/Gene/Summary?db=core;g=ENSG00000004142) | [POLDIP2](http://www.ensembl.org/Homo_sapiens/Gene/Summary?db=core;g=ENSG00000004142) |
| [ENSG00000172301](http://www.ensembl.org/Homo_sapiens/Gene/Summary?db=core;g=ENSG00000172301) | [COPRS](http://www.ensembl.org/Homo_sapiens/Gene/Summary?db=core;g=ENSG00000172301) |
| [ENSG00000108688](http://www.ensembl.org/Homo_sapiens/Gene/Summary?db=core;g=ENSG00000108688) | [CCL7](http://www.ensembl.org/Homo_sapiens/Gene/Summary?db=core;g=ENSG00000108688) |
| [ENSG00000198242](http://www.ensembl.org/Homo_sapiens/Gene/Summary?db=core;g=ENSG00000198242) | [RPL23A](http://www.ensembl.org/Homo_sapiens/Gene/Summary?db=core;g=ENSG00000198242) |
| [ENSG00000182271](http://www.ensembl.org/Homo_sapiens/Gene/Summary?db=core;g=ENSG00000182271) | [TMIGD1](http://www.ensembl.org/Homo_sapiens/Gene/Summary?db=core;g=ENSG00000182271) |
| [ENSG00000181481](http://www.ensembl.org/Homo_sapiens/Gene/Summary?db=core;g=ENSG00000181481) | [RNF135](http://www.ensembl.org/Homo_sapiens/Gene/Summary?db=core;g=ENSG00000181481) |
| [ENSG00000076382](http://www.ensembl.org/Homo_sapiens/Gene/Summary?db=core;g=ENSG00000076382) | [SPAG5](http://www.ensembl.org/Homo_sapiens/Gene/Summary?db=core;g=ENSG00000076382) |
| [ENSG00000108262](http://www.ensembl.org/Homo_sapiens/Gene/Summary?db=core;g=ENSG00000108262) | [GIT1](http://www.ensembl.org/Homo_sapiens/Gene/Summary?db=core;g=ENSG00000108262) |
| [ENSG00000073598](http://www.ensembl.org/Homo_sapiens/Gene/Summary?db=core;g=ENSG00000073598) | [FNDC8](http://www.ensembl.org/Homo_sapiens/Gene/Summary?db=core;g=ENSG00000073598) |
| [ENSG00000073536](http://www.ensembl.org/Homo_sapiens/Gene/Summary?db=core;g=ENSG00000073536) | [NLE1](http://www.ensembl.org/Homo_sapiens/Gene/Summary?db=core;g=ENSG00000073536) |
| [ENSG00000109113](http://www.ensembl.org/Homo_sapiens/Gene/Summary?db=core;g=ENSG00000109113) | [RAB34](http://www.ensembl.org/Homo_sapiens/Gene/Summary?db=core;g=ENSG00000109113) |
| [ENSG00000172171](http://www.ensembl.org/Homo_sapiens/Gene/Summary?db=core;g=ENSG00000172171) | [TEFM](http://www.ensembl.org/Homo_sapiens/Gene/Summary?db=core;g=ENSG00000172171) |
| [ENSG00000109103](http://www.ensembl.org/Homo_sapiens/Gene/Summary?db=core;g=ENSG00000109103) | [UNC119](http://www.ensembl.org/Homo_sapiens/Gene/Summary?db=core;g=ENSG00000109103) |
| [ENSG00000184060](http://www.ensembl.org/Homo_sapiens/Gene/Summary?db=core;g=ENSG00000184060) | [ADAP2](http://www.ensembl.org/Homo_sapiens/Gene/Summary?db=core;g=ENSG00000184060) |
| [ENSG00000172156](http://www.ensembl.org/Homo_sapiens/Gene/Summary?db=core;g=ENSG00000172156) | [CCL11](http://www.ensembl.org/Homo_sapiens/Gene/Summary?db=core;g=ENSG00000172156) |
| [ENSG00000141068](http://www.ensembl.org/Homo_sapiens/Gene/Summary?db=core;g=ENSG00000141068) | [KSR1](http://www.ensembl.org/Homo_sapiens/Gene/Summary?db=core;g=ENSG00000141068) |
| [ENSG00000007216](http://www.ensembl.org/Homo_sapiens/Gene/Summary?db=core;g=ENSG00000007216) | [SLC13A2](http://www.ensembl.org/Homo_sapiens/Gene/Summary?db=core;g=ENSG00000007216) |
| [ENSG00000160606](http://www.ensembl.org/Homo_sapiens/Gene/Summary?db=core;g=ENSG00000160606) | [TLCD1](http://www.ensembl.org/Homo_sapiens/Gene/Summary?db=core;g=ENSG00000160606) |
| [ENSG00000141314](http://www.ensembl.org/Homo_sapiens/Gene/Summary?db=core;g=ENSG00000141314) | [RHBDL3](http://www.ensembl.org/Homo_sapiens/Gene/Summary?db=core;g=ENSG00000141314) |
| [ENSG00000164729](http://www.ensembl.org/Homo_sapiens/Gene/Summary?db=core;g=ENSG00000164729) | [SLC35G3](http://www.ensembl.org/Homo_sapiens/Gene/Summary?db=core;g=ENSG00000164729) |
| [ENSG00000109107](http://www.ensembl.org/Homo_sapiens/Gene/Summary?db=core;g=ENSG00000109107) | [ALDOC](http://www.ensembl.org/Homo_sapiens/Gene/Summary?db=core;g=ENSG00000109107) |
| [ENSG00000108651](http://www.ensembl.org/Homo_sapiens/Gene/Summary?db=core;g=ENSG00000108651) | [UTP6](http://www.ensembl.org/Homo_sapiens/Gene/Summary?db=core;g=ENSG00000108651) |
| [ENSG00000108256](http://www.ensembl.org/Homo_sapiens/Gene/Summary?db=core;g=ENSG00000108256) | [NUFIP2](http://www.ensembl.org/Homo_sapiens/Gene/Summary?db=core;g=ENSG00000108256) |
| [ENSG00000141161](http://www.ensembl.org/Homo_sapiens/Gene/Summary?db=core;g=ENSG00000141161) | [UNC45B](http://www.ensembl.org/Homo_sapiens/Gene/Summary?db=core;g=ENSG00000141161) |
| [ENSG00000109084](http://www.ensembl.org/Homo_sapiens/Gene/Summary?db=core;g=ENSG00000109084) | [TMEM97](http://www.ensembl.org/Homo_sapiens/Gene/Summary?db=core;g=ENSG00000109084) |
| [ENSG00000236320](http://www.ensembl.org/Homo_sapiens/Gene/Summary?db=core;g=ENSG00000236320) | [SLFN14](http://www.ensembl.org/Homo_sapiens/Gene/Summary?db=core;g=ENSG00000236320) |
| [ENSG00000005156](http://www.ensembl.org/Homo_sapiens/Gene/Summary?db=core;g=ENSG00000005156) | [LIG3](http://www.ensembl.org/Homo_sapiens/Gene/Summary?db=core;g=ENSG00000005156) |
| [ENSG00000108666](http://www.ensembl.org/Homo_sapiens/Gene/Summary?db=core;g=ENSG00000108666) | [C17orf75](http://www.ensembl.org/Homo_sapiens/Gene/Summary?db=core;g=ENSG00000108666) |
| [ENSG00000108587](http://www.ensembl.org/Homo_sapiens/Gene/Summary?db=core;g=ENSG00000108587) | [GOSR1](http://www.ensembl.org/Homo_sapiens/Gene/Summary?db=core;g=ENSG00000108587) |
| [ENSG00000108684](http://www.ensembl.org/Homo_sapiens/Gene/Summary?db=core;g=ENSG00000108684) | [ASIC2](http://www.ensembl.org/Homo_sapiens/Gene/Summary?db=core;g=ENSG00000108684) |
| [ENSG00000167524](http://www.ensembl.org/Homo_sapiens/Gene/Summary?db=core;g=ENSG00000167524) | [SGK494](http://www.ensembl.org/Homo_sapiens/Gene/Summary?db=core;g=ENSG00000167524) |
| [ENSG00000167536](http://www.ensembl.org/Homo_sapiens/Gene/Summary?db=core;g=ENSG00000167536) | [DHRS13](http://www.ensembl.org/Homo_sapiens/Gene/Summary?db=core;g=ENSG00000167536) |
| [ENSG00000167549](http://www.ensembl.org/Homo_sapiens/Gene/Summary?db=core;g=ENSG00000167549) | [CORO6](http://www.ensembl.org/Homo_sapiens/Gene/Summary?db=core;g=ENSG00000167549) |
| [ENSG00000141298](http://www.ensembl.org/Homo_sapiens/Gene/Summary?db=core;g=ENSG00000141298) | [SSH2](http://www.ensembl.org/Homo_sapiens/Gene/Summary?db=core;g=ENSG00000141298) |
| [ENSG00000176927](http://www.ensembl.org/Homo_sapiens/Gene/Summary?db=core;g=ENSG00000176927) | [EFCAB5](http://www.ensembl.org/Homo_sapiens/Gene/Summary?db=core;g=ENSG00000176927) |
| [ENSG00000221995](http://www.ensembl.org/Homo_sapiens/Gene/Summary?db=core;g=ENSG00000221995) | [TIAF1](http://www.ensembl.org/Homo_sapiens/Gene/Summary?db=core;g=ENSG00000221995) |
| [ENSG00000100297](http://www.ensembl.org/Homo_sapiens/Gene/Summary?db=core;g=ENSG00000100297) | [MCM5](http://www.ensembl.org/Homo_sapiens/Gene/Summary?db=core;g=ENSG00000100297) |
| [ENSG00000100220](http://www.ensembl.org/Homo_sapiens/Gene/Summary?db=core;g=ENSG00000100220) | [RTCB](http://www.ensembl.org/Homo_sapiens/Gene/Summary?db=core;g=ENSG00000100220) |
| [ENSG00000182541](http://www.ensembl.org/Homo_sapiens/Gene/Summary?db=core;g=ENSG00000182541) | [LIMK2](http://www.ensembl.org/Homo_sapiens/Gene/Summary?db=core;g=ENSG00000182541) |
| [ENSG00000100348](http://www.ensembl.org/Homo_sapiens/Gene/Summary?db=core;g=ENSG00000100348) | [TXN2](http://www.ensembl.org/Homo_sapiens/Gene/Summary?db=core;g=ENSG00000100348) |
| [ENSG00000128298](http://www.ensembl.org/Homo_sapiens/Gene/Summary?db=core;g=ENSG00000128298) | [BAIAP2L2](http://www.ensembl.org/Homo_sapiens/Gene/Summary?db=core;g=ENSG00000128298) |
| [ENSG00000221890](http://www.ensembl.org/Homo_sapiens/Gene/Summary?db=core;g=ENSG00000221890) | [NPTXR](http://www.ensembl.org/Homo_sapiens/Gene/Summary?db=core;g=ENSG00000221890) |
| [ENSG00000168135](http://www.ensembl.org/Homo_sapiens/Gene/Summary?db=core;g=ENSG00000168135) | [KCNJ4](http://www.ensembl.org/Homo_sapiens/Gene/Summary?db=core;g=ENSG00000168135) |
| [ENSG00000100302](http://www.ensembl.org/Homo_sapiens/Gene/Summary?db=core;g=ENSG00000100302) | [RASD2](http://www.ensembl.org/Homo_sapiens/Gene/Summary?db=core;g=ENSG00000100302) |
| [ENSG00000100191](http://www.ensembl.org/Homo_sapiens/Gene/Summary?db=core;g=ENSG00000100191) | [SLC5A4](http://www.ensembl.org/Homo_sapiens/Gene/Summary?db=core;g=ENSG00000100191) |
| [ENSG00000133466](http://www.ensembl.org/Homo_sapiens/Gene/Summary?db=core;g=ENSG00000133466) | [C1QTNF6](http://www.ensembl.org/Homo_sapiens/Gene/Summary?db=core;g=ENSG00000133466) |
| [ENSG00000175329](http://www.ensembl.org/Homo_sapiens/Gene/Summary?db=core;g=ENSG00000175329) | [ISX](http://www.ensembl.org/Homo_sapiens/Gene/Summary?db=core;g=ENSG00000175329) |
| [ENSG00000100201](http://www.ensembl.org/Homo_sapiens/Gene/Summary?db=core;g=ENSG00000100201) | [DDX17](http://www.ensembl.org/Homo_sapiens/Gene/Summary?db=core;g=ENSG00000100201) |
| [ENSG00000100105](http://www.ensembl.org/Homo_sapiens/Gene/Summary?db=core;g=ENSG00000100105) | [PATZ1](http://www.ensembl.org/Homo_sapiens/Gene/Summary?db=core;g=ENSG00000100105) |
| [ENSG00000100139](http://www.ensembl.org/Homo_sapiens/Gene/Summary?db=core;g=ENSG00000100139) | [MICALL1](http://www.ensembl.org/Homo_sapiens/Gene/Summary?db=core;g=ENSG00000100139) |
| [ENSG00000187045](http://www.ensembl.org/Homo_sapiens/Gene/Summary?db=core;g=ENSG00000187045) | [TMPRSS6](http://www.ensembl.org/Homo_sapiens/Gene/Summary?db=core;g=ENSG00000187045) |
| [ENSG00000128342](http://www.ensembl.org/Homo_sapiens/Gene/Summary?db=core;g=ENSG00000128342) | [LIF](http://www.ensembl.org/Homo_sapiens/Gene/Summary?db=core;g=ENSG00000128342) |
| [ENSG00000100151](http://www.ensembl.org/Homo_sapiens/Gene/Summary?db=core;g=ENSG00000100151) | [PICK1](http://www.ensembl.org/Homo_sapiens/Gene/Summary?db=core;g=ENSG00000100151) |
| [ENSG00000185339](http://www.ensembl.org/Homo_sapiens/Gene/Summary?db=core;g=ENSG00000185339) | [TCN2](http://www.ensembl.org/Homo_sapiens/Gene/Summary?db=core;g=ENSG00000185339) |
| [ENSG00000100060](http://www.ensembl.org/Homo_sapiens/Gene/Summary?db=core;g=ENSG00000100060) | [MFNG](http://www.ensembl.org/Homo_sapiens/Gene/Summary?db=core;g=ENSG00000100060) |
| [ENSG00000100196](http://www.ensembl.org/Homo_sapiens/Gene/Summary?db=core;g=ENSG00000100196) | [KDELR3](http://www.ensembl.org/Homo_sapiens/Gene/Summary?db=core;g=ENSG00000100196) |
| [ENSG00000100106](http://www.ensembl.org/Homo_sapiens/Gene/Summary?db=core;g=ENSG00000100106) | [TRIOBP](http://www.ensembl.org/Homo_sapiens/Gene/Summary?db=core;g=ENSG00000100106) |
| [ENSG00000100225](http://www.ensembl.org/Homo_sapiens/Gene/Summary?db=core;g=ENSG00000100225) | [FBXO7](http://www.ensembl.org/Homo_sapiens/Gene/Summary?db=core;g=ENSG00000100225) |
| [ENSG00000184381](http://www.ensembl.org/Homo_sapiens/Gene/Summary?db=core;g=ENSG00000184381) | [PLA2G6](http://www.ensembl.org/Homo_sapiens/Gene/Summary?db=core;g=ENSG00000184381) |
| [ENSG00000128340](http://www.ensembl.org/Homo_sapiens/Gene/Summary?db=core;g=ENSG00000128340) | [RAC2](http://www.ensembl.org/Homo_sapiens/Gene/Summary?db=core;g=ENSG00000128340) |
| [ENSG00000100335](http://www.ensembl.org/Homo_sapiens/Gene/Summary?db=core;g=ENSG00000100335) | [MIEF1](http://www.ensembl.org/Homo_sapiens/Gene/Summary?db=core;g=ENSG00000100335) |
| [ENSG00000198792](http://www.ensembl.org/Homo_sapiens/Gene/Summary?db=core;g=ENSG00000198792) | [TMEM184B](http://www.ensembl.org/Homo_sapiens/Gene/Summary?db=core;g=ENSG00000198792) |
| [ENSG00000100003](http://www.ensembl.org/Homo_sapiens/Gene/Summary?db=core;g=ENSG00000100003) | [SEC14L2](http://www.ensembl.org/Homo_sapiens/Gene/Summary?db=core;g=ENSG00000100003) |
| [ENSG00000100324](http://www.ensembl.org/Homo_sapiens/Gene/Summary?db=core;g=ENSG00000100324) | [TAB1](http://www.ensembl.org/Homo_sapiens/Gene/Summary?db=core;g=ENSG00000100324) |
| [ENSG00000100292](http://www.ensembl.org/Homo_sapiens/Gene/Summary?db=core;g=ENSG00000100292) | [HMOX1](http://www.ensembl.org/Homo_sapiens/Gene/Summary?db=core;g=ENSG00000100292) |
| [ENSG00000128346](http://www.ensembl.org/Homo_sapiens/Gene/Summary?db=core;g=ENSG00000128346) | [C22orf23](http://www.ensembl.org/Homo_sapiens/Gene/Summary?db=core;g=ENSG00000128346) |
| [ENSG00000100368](http://www.ensembl.org/Homo_sapiens/Gene/Summary?db=core;g=ENSG00000100368) | [CSF2RB](http://www.ensembl.org/Homo_sapiens/Gene/Summary?db=core;g=ENSG00000100368) |
| [ENSG00000128253](http://www.ensembl.org/Homo_sapiens/Gene/Summary?db=core;g=ENSG00000128253) | [RFPL2](http://www.ensembl.org/Homo_sapiens/Gene/Summary?db=core;g=ENSG00000128253) |
| [ENSG00000128383](http://www.ensembl.org/Homo_sapiens/Gene/Summary?db=core;g=ENSG00000128383) | [APOBEC3A](http://www.ensembl.org/Homo_sapiens/Gene/Summary?db=core;g=ENSG00000128383) |
| [ENSG00000099992](http://www.ensembl.org/Homo_sapiens/Gene/Summary?db=core;g=ENSG00000099992) | [TBC1D10A](http://www.ensembl.org/Homo_sapiens/Gene/Summary?db=core;g=ENSG00000099992) |
| [ENSG00000128335](http://www.ensembl.org/Homo_sapiens/Gene/Summary?db=core;g=ENSG00000128335) | [APOL2](http://www.ensembl.org/Homo_sapiens/Gene/Summary?db=core;g=ENSG00000128335) |
| [ENSG00000100379](http://www.ensembl.org/Homo_sapiens/Gene/Summary?db=core;g=ENSG00000100379) | [KCTD17](http://www.ensembl.org/Homo_sapiens/Gene/Summary?db=core;g=ENSG00000100379) |
| [ENSG00000100362](http://www.ensembl.org/Homo_sapiens/Gene/Summary?db=core;g=ENSG00000100362) | [PVALB](http://www.ensembl.org/Homo_sapiens/Gene/Summary?db=core;g=ENSG00000100362) |
| [ENSG00000100385](http://www.ensembl.org/Homo_sapiens/Gene/Summary?db=core;g=ENSG00000100385) | [IL2RB](http://www.ensembl.org/Homo_sapiens/Gene/Summary?db=core;g=ENSG00000100385) |
| [ENSG00000100216](http://www.ensembl.org/Homo_sapiens/Gene/Summary?db=core;g=ENSG00000100216) | [TOMM22](http://www.ensembl.org/Homo_sapiens/Gene/Summary?db=core;g=ENSG00000100216) |
| [ENSG00000241360](http://www.ensembl.org/Homo_sapiens/Gene/Summary?db=core;g=ENSG00000241360) | [PDXP](http://www.ensembl.org/Homo_sapiens/Gene/Summary?db=core;g=ENSG00000241360) |
| [ENSG00000198089](http://www.ensembl.org/Homo_sapiens/Gene/Summary?db=core;g=ENSG00000198089) | [SFI1](http://www.ensembl.org/Homo_sapiens/Gene/Summary?db=core;g=ENSG00000198089) |
| [ENSG00000128242](http://www.ensembl.org/Homo_sapiens/Gene/Summary?db=core;g=ENSG00000128242) | [GAL3ST1](http://www.ensembl.org/Homo_sapiens/Gene/Summary?db=core;g=ENSG00000128242) |
| [ENSG00000100097](http://www.ensembl.org/Homo_sapiens/Gene/Summary?db=core;g=ENSG00000100097) | [LGALS1](http://www.ensembl.org/Homo_sapiens/Gene/Summary?db=core;g=ENSG00000100097) |
| [ENSG00000243811](http://www.ensembl.org/Homo_sapiens/Gene/Summary?db=core;g=ENSG00000243811) | [APOBEC3D](http://www.ensembl.org/Homo_sapiens/Gene/Summary?db=core;g=ENSG00000243811) |
| [ENSG00000278195](http://www.ensembl.org/Homo_sapiens/Gene/Summary?db=core;g=ENSG00000278195) | [SSTR3](http://www.ensembl.org/Homo_sapiens/Gene/Summary?db=core;g=ENSG00000278195) |
| [ENSG00000273899](http://www.ensembl.org/Homo_sapiens/Gene/Summary?db=core;g=ENSG00000273899) | [NOL12](http://www.ensembl.org/Homo_sapiens/Gene/Summary?db=core;g=ENSG00000273899) |
| [ENSG00000100345](http://www.ensembl.org/Homo_sapiens/Gene/Summary?db=core;g=ENSG00000100345) | [MYH9](http://www.ensembl.org/Homo_sapiens/Gene/Summary?db=core;g=ENSG00000100345) |
| [ENSG00000100170](http://www.ensembl.org/Homo_sapiens/Gene/Summary?db=core;g=ENSG00000100170) | [SLC5A1](http://www.ensembl.org/Homo_sapiens/Gene/Summary?db=core;g=ENSG00000100170) |
| [ENSG00000100365](http://www.ensembl.org/Homo_sapiens/Gene/Summary?db=core;g=ENSG00000100365) | [NCF4](http://www.ensembl.org/Homo_sapiens/Gene/Summary?db=core;g=ENSG00000100365) |
| [ENSG00000100321](http://www.ensembl.org/Homo_sapiens/Gene/Summary?db=core;g=ENSG00000100321) | [SYNGR1](http://www.ensembl.org/Homo_sapiens/Gene/Summary?db=core;g=ENSG00000100321) |
| [ENSG00000179750](http://www.ensembl.org/Homo_sapiens/Gene/Summary?db=core;g=ENSG00000179750) | [APOBEC3B](http://www.ensembl.org/Homo_sapiens/Gene/Summary?db=core;g=ENSG00000179750) |
| [ENSG00000100156](http://www.ensembl.org/Homo_sapiens/Gene/Summary?db=core;g=ENSG00000100156) | [SLC16A8](http://www.ensembl.org/Homo_sapiens/Gene/Summary?db=core;g=ENSG00000100156) |
| [ENSG00000100142](http://www.ensembl.org/Homo_sapiens/Gene/Summary?db=core;g=ENSG00000100142) | [POLR2F](http://www.ensembl.org/Homo_sapiens/Gene/Summary?db=core;g=ENSG00000100142) |
| [ENSG00000100083](http://www.ensembl.org/Homo_sapiens/Gene/Summary?db=core;g=ENSG00000100083) | [GGA1](http://www.ensembl.org/Homo_sapiens/Gene/Summary?db=core;g=ENSG00000100083) |
| [ENSG00000128310](http://www.ensembl.org/Homo_sapiens/Gene/Summary?db=core;g=ENSG00000128310) | [GALR3](http://www.ensembl.org/Homo_sapiens/Gene/Summary?db=core;g=ENSG00000128310) |
| [ENSG00000100146](http://www.ensembl.org/Homo_sapiens/Gene/Summary?db=core;g=ENSG00000100146) | [SOX10](http://www.ensembl.org/Homo_sapiens/Gene/Summary?db=core;g=ENSG00000100146) |
| [ENSG00000176635](http://www.ensembl.org/Homo_sapiens/Gene/Summary?db=core;g=ENSG00000176635) | [HORMAD2](http://www.ensembl.org/Homo_sapiens/Gene/Summary?db=core;g=ENSG00000176635) |
| [ENSG00000100221](http://www.ensembl.org/Homo_sapiens/Gene/Summary?db=core;g=ENSG00000100221) | [JOSD1](http://www.ensembl.org/Homo_sapiens/Gene/Summary?db=core;g=ENSG00000100221) |
| [ENSG00000241878](http://www.ensembl.org/Homo_sapiens/Gene/Summary?db=core;g=ENSG00000241878) | [PISD](http://www.ensembl.org/Homo_sapiens/Gene/Summary?db=core;g=ENSG00000241878) |
| [ENSG00000189060](http://www.ensembl.org/Homo_sapiens/Gene/Summary?db=core;g=ENSG00000189060) | [H1F0](http://www.ensembl.org/Homo_sapiens/Gene/Summary?db=core;g=ENSG00000189060) |
| [ENSG00000099995](http://www.ensembl.org/Homo_sapiens/Gene/Summary?db=core;g=ENSG00000099995) | [SF3A1](http://www.ensembl.org/Homo_sapiens/Gene/Summary?db=core;g=ENSG00000099995) |
| [ENSG00000242114](http://www.ensembl.org/Homo_sapiens/Gene/Summary?db=core;g=ENSG00000242114) | [MTFP1](http://www.ensembl.org/Homo_sapiens/Gene/Summary?db=core;g=ENSG00000242114) |
| [ENSG00000099999](http://www.ensembl.org/Homo_sapiens/Gene/Summary?db=core;g=ENSG00000099999) | [RNF215](http://www.ensembl.org/Homo_sapiens/Gene/Summary?db=core;g=ENSG00000099999) |
| [ENSG00000100092](http://www.ensembl.org/Homo_sapiens/Gene/Summary?db=core;g=ENSG00000100092) | [SH3BP1](http://www.ensembl.org/Homo_sapiens/Gene/Summary?db=core;g=ENSG00000100092) |
| [ENSG00000100036](http://www.ensembl.org/Homo_sapiens/Gene/Summary?db=core;g=ENSG00000100036) | [SLC35E4](http://www.ensembl.org/Homo_sapiens/Gene/Summary?db=core;g=ENSG00000100036) |
| [ENSG00000100226](http://www.ensembl.org/Homo_sapiens/Gene/Summary?db=core;g=ENSG00000100226) | [GTPBP1](http://www.ensembl.org/Homo_sapiens/Gene/Summary?db=core;g=ENSG00000100226) |
| [ENSG00000128272](http://www.ensembl.org/Homo_sapiens/Gene/Summary?db=core;g=ENSG00000128272) | [ATF4](http://www.ensembl.org/Homo_sapiens/Gene/Summary?db=core;g=ENSG00000128272) |
| [ENSG00000213923](http://www.ensembl.org/Homo_sapiens/Gene/Summary?db=core;g=ENSG00000213923) | [CSNK1E](http://www.ensembl.org/Homo_sapiens/Gene/Summary?db=core;g=ENSG00000213923) |
| [ENSG00000100206](http://www.ensembl.org/Homo_sapiens/Gene/Summary?db=core;g=ENSG00000100206) | [DMC1](http://www.ensembl.org/Homo_sapiens/Gene/Summary?db=core;g=ENSG00000100206) |
| [ENSG00000128283](http://www.ensembl.org/Homo_sapiens/Gene/Summary?db=core;g=ENSG00000128283) | [CDC42EP1](http://www.ensembl.org/Homo_sapiens/Gene/Summary?db=core;g=ENSG00000128283) |
| [ENSG00000244509](http://www.ensembl.org/Homo_sapiens/Gene/Summary?db=core;g=ENSG00000244509) | [APOBEC3C](http://www.ensembl.org/Homo_sapiens/Gene/Summary?db=core;g=ENSG00000244509) |
| [ENSG00000167065](http://www.ensembl.org/Homo_sapiens/Gene/Summary?db=core;g=ENSG00000167065) | [DUSP18](http://www.ensembl.org/Homo_sapiens/Gene/Summary?db=core;g=ENSG00000167065) |
| [ENSG00000184459](http://www.ensembl.org/Homo_sapiens/Gene/Summary?db=core;g=ENSG00000184459) | [BPIFC](http://www.ensembl.org/Homo_sapiens/Gene/Summary?db=core;g=ENSG00000184459) |
| [ENSG00000133488](http://www.ensembl.org/Homo_sapiens/Gene/Summary?db=core;g=ENSG00000133488) | [SEC14L4](http://www.ensembl.org/Homo_sapiens/Gene/Summary?db=core;g=ENSG00000133488) |
| [ENSG00000100129](http://www.ensembl.org/Homo_sapiens/Gene/Summary?db=core;g=ENSG00000100129) | [EIF3L](http://www.ensembl.org/Homo_sapiens/Gene/Summary?db=core;g=ENSG00000100129) |
| [ENSG00000183963](http://www.ensembl.org/Homo_sapiens/Gene/Summary?db=core;g=ENSG00000183963) | [SMTN](http://www.ensembl.org/Homo_sapiens/Gene/Summary?db=core;g=ENSG00000183963) |
| [ENSG00000100346](http://www.ensembl.org/Homo_sapiens/Gene/Summary?db=core;g=ENSG00000100346) | [CACNA1I](http://www.ensembl.org/Homo_sapiens/Gene/Summary?db=core;g=ENSG00000100346) |
| [ENSG00000100284](http://www.ensembl.org/Homo_sapiens/Gene/Summary?db=core;g=ENSG00000100284) | [TOM1](http://www.ensembl.org/Homo_sapiens/Gene/Summary?db=core;g=ENSG00000100284) |
| [ENSG00000100330](http://www.ensembl.org/Homo_sapiens/Gene/Summary?db=core;g=ENSG00000100330) | [MTMR3](http://www.ensembl.org/Homo_sapiens/Gene/Summary?db=core;g=ENSG00000100330) |
| [ENSG00000214491](http://www.ensembl.org/Homo_sapiens/Gene/Summary?db=core;g=ENSG00000214491) | [SEC14L6](http://www.ensembl.org/Homo_sapiens/Gene/Summary?db=core;g=ENSG00000214491) |
| [ENSG00000100360](http://www.ensembl.org/Homo_sapiens/Gene/Summary?db=core;g=ENSG00000100360) | [IFT27](http://www.ensembl.org/Homo_sapiens/Gene/Summary?db=core;g=ENSG00000100360) |
| [ENSG00000100100](http://www.ensembl.org/Homo_sapiens/Gene/Summary?db=core;g=ENSG00000100100) | [PIK3IP1](http://www.ensembl.org/Homo_sapiens/Gene/Summary?db=core;g=ENSG00000100100) |
| [ENSG00000100242](http://www.ensembl.org/Homo_sapiens/Gene/Summary?db=core;g=ENSG00000100242) | [SUN2](http://www.ensembl.org/Homo_sapiens/Gene/Summary?db=core;g=ENSG00000100242) |
| [ENSG00000100012](http://www.ensembl.org/Homo_sapiens/Gene/Summary?db=core;g=ENSG00000100012) | [SEC14L3](http://www.ensembl.org/Homo_sapiens/Gene/Summary?db=core;g=ENSG00000100012) |
| [ENSG00000138942](http://www.ensembl.org/Homo_sapiens/Gene/Summary?db=core;g=ENSG00000138942) | [RNF185](http://www.ensembl.org/Homo_sapiens/Gene/Summary?db=core;g=ENSG00000138942) |
| [ENSG00000128276](http://www.ensembl.org/Homo_sapiens/Gene/Summary?db=core;g=ENSG00000128276) | [RFPL3](http://www.ensembl.org/Homo_sapiens/Gene/Summary?db=core;g=ENSG00000128276) |
| [ENSG00000100350](http://www.ensembl.org/Homo_sapiens/Gene/Summary?db=core;g=ENSG00000100350) | [FOXRED2](http://www.ensembl.org/Homo_sapiens/Gene/Summary?db=core;g=ENSG00000100350) |
| [ENSG00000185022](http://www.ensembl.org/Homo_sapiens/Gene/Summary?db=core;g=ENSG00000185022) | [MAFF](http://www.ensembl.org/Homo_sapiens/Gene/Summary?db=core;g=ENSG00000185022) |
| [ENSG00000100281](http://www.ensembl.org/Homo_sapiens/Gene/Summary?db=core;g=ENSG00000100281) | [HMGXB4](http://www.ensembl.org/Homo_sapiens/Gene/Summary?db=core;g=ENSG00000100281) |
| [ENSG00000100320](http://www.ensembl.org/Homo_sapiens/Gene/Summary?db=core;g=ENSG00000100320) | [RBFOX2](http://www.ensembl.org/Homo_sapiens/Gene/Summary?db=core;g=ENSG00000100320) |
| [ENSG00000128309](http://www.ensembl.org/Homo_sapiens/Gene/Summary?db=core;g=ENSG00000128309) | [MPST](http://www.ensembl.org/Homo_sapiens/Gene/Summary?db=core;g=ENSG00000128309) |
| [ENSG00000100116](http://www.ensembl.org/Homo_sapiens/Gene/Summary?db=core;g=ENSG00000100116) | [GCAT](http://www.ensembl.org/Homo_sapiens/Gene/Summary?db=core;g=ENSG00000100116) |
| [ENSG00000187051](http://www.ensembl.org/Homo_sapiens/Gene/Summary?db=core;g=ENSG00000187051) | [RPS19BP1](http://www.ensembl.org/Homo_sapiens/Gene/Summary?db=core;g=ENSG00000187051) |
| [ENSG00000133424](http://www.ensembl.org/Homo_sapiens/Gene/Summary?db=core;g=ENSG00000133424) | [LARGE](http://www.ensembl.org/Homo_sapiens/Gene/Summary?db=core;g=ENSG00000133424) |
| [ENSG00000128313](http://www.ensembl.org/Homo_sapiens/Gene/Summary?db=core;g=ENSG00000128313) | [APOL5](http://www.ensembl.org/Homo_sapiens/Gene/Summary?db=core;g=ENSG00000128313) |
| [ENSG00000100307](http://www.ensembl.org/Homo_sapiens/Gene/Summary?db=core;g=ENSG00000100307) | [CBX7](http://www.ensembl.org/Homo_sapiens/Gene/Summary?db=core;g=ENSG00000100307) |
| [ENSG00000100234](http://www.ensembl.org/Homo_sapiens/Gene/Summary?db=core;g=ENSG00000100234) | [TIMP3](http://www.ensembl.org/Homo_sapiens/Gene/Summary?db=core;g=ENSG00000100234) |
| [ENSG00000128284](http://www.ensembl.org/Homo_sapiens/Gene/Summary?db=core;g=ENSG00000128284) | [APOL3](http://www.ensembl.org/Homo_sapiens/Gene/Summary?db=core;g=ENSG00000128284) |
| [ENSG00000185721](http://www.ensembl.org/Homo_sapiens/Gene/Summary?db=core;g=ENSG00000185721) | [DRG1](http://www.ensembl.org/Homo_sapiens/Gene/Summary?db=core;g=ENSG00000185721) |
| [ENSG00000239282](http://www.ensembl.org/Homo_sapiens/Gene/Summary?db=core;g=ENSG00000239282) | [GATSL3](http://www.ensembl.org/Homo_sapiens/Gene/Summary?db=core;g=ENSG00000239282) |
| [ENSG00000128311](http://www.ensembl.org/Homo_sapiens/Gene/Summary?db=core;g=ENSG00000128311) | [TST](http://www.ensembl.org/Homo_sapiens/Gene/Summary?db=core;g=ENSG00000128311) |
| [ENSG00000100342](http://www.ensembl.org/Homo_sapiens/Gene/Summary?db=core;g=ENSG00000100342) | [APOL1](http://www.ensembl.org/Homo_sapiens/Gene/Summary?db=core;g=ENSG00000100342) |
| [ENSG00000100055](http://www.ensembl.org/Homo_sapiens/Gene/Summary?db=core;g=ENSG00000100055) | [CYTH4](http://www.ensembl.org/Homo_sapiens/Gene/Summary?db=core;g=ENSG00000100055) |
| [ENSG00000099985](http://www.ensembl.org/Homo_sapiens/Gene/Summary?db=core;g=ENSG00000099985) | [OSM](http://www.ensembl.org/Homo_sapiens/Gene/Summary?db=core;g=ENSG00000099985) |
| [ENSG00000100311](http://www.ensembl.org/Homo_sapiens/Gene/Summary?db=core;g=ENSG00000100311) | [PDGFB](http://www.ensembl.org/Homo_sapiens/Gene/Summary?db=core;g=ENSG00000100311) |
| [ENSG00000185666](http://www.ensembl.org/Homo_sapiens/Gene/Summary?db=core;g=ENSG00000185666) | [SYN3](http://www.ensembl.org/Homo_sapiens/Gene/Summary?db=core;g=ENSG00000185666) |
| [ENSG00000166862](http://www.ensembl.org/Homo_sapiens/Gene/Summary?db=core;g=ENSG00000166862) | [CACNG2](http://www.ensembl.org/Homo_sapiens/Gene/Summary?db=core;g=ENSG00000166862) |
| [ENSG00000100298](http://www.ensembl.org/Homo_sapiens/Gene/Summary?db=core;g=ENSG00000100298) | [APOBEC3H](http://www.ensembl.org/Homo_sapiens/Gene/Summary?db=core;g=ENSG00000100298) |
| [ENSG00000100211](http://www.ensembl.org/Homo_sapiens/Gene/Summary?db=core;g=ENSG00000100211) | [CBY1](http://www.ensembl.org/Homo_sapiens/Gene/Summary?db=core;g=ENSG00000100211) |
| [ENSG00000100316](http://www.ensembl.org/Homo_sapiens/Gene/Summary?db=core;g=ENSG00000100316) | [RPL3](http://www.ensembl.org/Homo_sapiens/Gene/Summary?db=core;g=ENSG00000100316) |
| [ENSG00000184792](http://www.ensembl.org/Homo_sapiens/Gene/Summary?db=core;g=ENSG00000184792) | [OSBP2](http://www.ensembl.org/Homo_sapiens/Gene/Summary?db=core;g=ENSG00000184792) |
| [ENSG00000100029](http://www.ensembl.org/Homo_sapiens/Gene/Summary?db=core;g=ENSG00000100029) | [PES1](http://www.ensembl.org/Homo_sapiens/Gene/Summary?db=core;g=ENSG00000100029) |
| [ENSG00000198832](http://www.ensembl.org/Homo_sapiens/Gene/Summary?db=core;g=ENSG00000198832) | [SELM](http://www.ensembl.org/Homo_sapiens/Gene/Summary?db=core;g=ENSG00000198832) |
| [ENSG00000166897](http://www.ensembl.org/Homo_sapiens/Gene/Summary?db=core;g=ENSG00000166897) | [ELFN2](http://www.ensembl.org/Homo_sapiens/Gene/Summary?db=core;g=ENSG00000166897) |
| [ENSG00000185133](http://www.ensembl.org/Homo_sapiens/Gene/Summary?db=core;g=ENSG00000185133) | [INPP5J](http://www.ensembl.org/Homo_sapiens/Gene/Summary?db=core;g=ENSG00000185133) |
| [ENSG00000100353](http://www.ensembl.org/Homo_sapiens/Gene/Summary?db=core;g=ENSG00000100353) | [EIF3D](http://www.ensembl.org/Homo_sapiens/Gene/Summary?db=core;g=ENSG00000100353) |
| [ENSG00000184708](http://www.ensembl.org/Homo_sapiens/Gene/Summary?db=core;g=ENSG00000184708) | [EIF4ENIF1](http://www.ensembl.org/Homo_sapiens/Gene/Summary?db=core;g=ENSG00000184708) |
| [ENSG00000100325](http://www.ensembl.org/Homo_sapiens/Gene/Summary?db=core;g=ENSG00000100325) | [ASCC2](http://www.ensembl.org/Homo_sapiens/Gene/Summary?db=core;g=ENSG00000100325) |
| [ENSG00000100124](http://www.ensembl.org/Homo_sapiens/Gene/Summary?db=core;g=ENSG00000100124) | [ANKRD54](http://www.ensembl.org/Homo_sapiens/Gene/Summary?db=core;g=ENSG00000100124) |
| [ENSG00000128268](http://www.ensembl.org/Homo_sapiens/Gene/Summary?db=core;g=ENSG00000128268) | [MGAT3](http://www.ensembl.org/Homo_sapiens/Gene/Summary?db=core;g=ENSG00000128268) |
| [ENSG00000100078](http://www.ensembl.org/Homo_sapiens/Gene/Summary?db=core;g=ENSG00000100078) | [PLA2G3](http://www.ensembl.org/Homo_sapiens/Gene/Summary?db=core;g=ENSG00000100078) |
| [ENSG00000128245](http://www.ensembl.org/Homo_sapiens/Gene/Summary?db=core;g=ENSG00000128245) | [YWHAH](http://www.ensembl.org/Homo_sapiens/Gene/Summary?db=core;g=ENSG00000128245) |
| [ENSG00000221963](http://www.ensembl.org/Homo_sapiens/Gene/Summary?db=core;g=ENSG00000221963) | [APOL6](http://www.ensembl.org/Homo_sapiens/Gene/Summary?db=core;g=ENSG00000221963) |
| [ENSG00000100079](http://www.ensembl.org/Homo_sapiens/Gene/Summary?db=core;g=ENSG00000100079) | [LGALS2](http://www.ensembl.org/Homo_sapiens/Gene/Summary?db=core;g=ENSG00000100079) |
| [ENSG00000100336](http://www.ensembl.org/Homo_sapiens/Gene/Summary?db=core;g=ENSG00000100336) | [APOL4](http://www.ensembl.org/Homo_sapiens/Gene/Summary?db=core;g=ENSG00000100336) |
| [ENSG00000100065](http://www.ensembl.org/Homo_sapiens/Gene/Summary?db=core;g=ENSG00000100065) | [CARD10](http://www.ensembl.org/Homo_sapiens/Gene/Summary?db=core;g=ENSG00000100065) |
| [ENSG00000249590](http://www.ensembl.org/Homo_sapiens/Gene/Summary?db=core;g=ENSG00000249590) | [RP4-539M6.19](http://www.ensembl.org/Homo_sapiens/Gene/Summary?db=core;g=ENSG00000249590) |
| [ENSG00000198125](http://www.ensembl.org/Homo_sapiens/Gene/Summary?db=core;g=ENSG00000198125) | [MB](http://www.ensembl.org/Homo_sapiens/Gene/Summary?db=core;g=ENSG00000198125) |
| [ENSG00000133422](http://www.ensembl.org/Homo_sapiens/Gene/Summary?db=core;g=ENSG00000133422) | [MORC2](http://www.ensembl.org/Homo_sapiens/Gene/Summary?db=core;g=ENSG00000133422) |
| [ENSG00000183741](http://www.ensembl.org/Homo_sapiens/Gene/Summary?db=core;g=ENSG00000183741) | [CBX6](http://www.ensembl.org/Homo_sapiens/Gene/Summary?db=core;g=ENSG00000183741) |
| [ENSG00000239713](http://www.ensembl.org/Homo_sapiens/Gene/Summary?db=core;g=ENSG00000239713) | [APOBEC3G](http://www.ensembl.org/Homo_sapiens/Gene/Summary?db=core;g=ENSG00000239713) |
| [ENSG00000128394](http://www.ensembl.org/Homo_sapiens/Gene/Summary?db=core;g=ENSG00000128394) | [APOBEC3F](http://www.ensembl.org/Homo_sapiens/Gene/Summary?db=core;g=ENSG00000128394) |
| [ENSG00000100150](http://www.ensembl.org/Homo_sapiens/Gene/Summary?db=core;g=ENSG00000100150) | [DEPDC5](http://www.ensembl.org/Homo_sapiens/Gene/Summary?db=core;g=ENSG00000100150) |
| [ENSG00000100246](http://www.ensembl.org/Homo_sapiens/Gene/Summary?db=core;g=ENSG00000100246) | [DNAL4](http://www.ensembl.org/Homo_sapiens/Gene/Summary?db=core;g=ENSG00000100246) |
| [ENSG00000144908](http://www.ensembl.org/Homo_sapiens/Gene/Summary?db=core;g=ENSG00000144908) | [ALDH1L1](http://www.ensembl.org/Homo_sapiens/Gene/Summary?db=core;g=ENSG00000144908) |
| [ENSG00000138496](http://www.ensembl.org/Homo_sapiens/Gene/Summary?db=core;g=ENSG00000138496) | [PARP9](http://www.ensembl.org/Homo_sapiens/Gene/Summary?db=core;g=ENSG00000138496) |
| [ENSG00000145088](http://www.ensembl.org/Homo_sapiens/Gene/Summary?db=core;g=ENSG00000145088) | [EAF2](http://www.ensembl.org/Homo_sapiens/Gene/Summary?db=core;g=ENSG00000145088) |
| [ENSG00000221955](http://www.ensembl.org/Homo_sapiens/Gene/Summary?db=core;g=ENSG00000221955) | [SLC12A8](http://www.ensembl.org/Homo_sapiens/Gene/Summary?db=core;g=ENSG00000221955) |
| [ENSG00000121853](http://www.ensembl.org/Homo_sapiens/Gene/Summary?db=core;g=ENSG00000121853) | [GHSR](http://www.ensembl.org/Homo_sapiens/Gene/Summary?db=core;g=ENSG00000121853) |
| [ENSG00000114023](http://www.ensembl.org/Homo_sapiens/Gene/Summary?db=core;g=ENSG00000114023) | [FAM162A](http://www.ensembl.org/Homo_sapiens/Gene/Summary?db=core;g=ENSG00000114023) |
| [ENSG00000186103](http://www.ensembl.org/Homo_sapiens/Gene/Summary?db=core;g=ENSG00000186103) | [ARGFX](http://www.ensembl.org/Homo_sapiens/Gene/Summary?db=core;g=ENSG00000186103) |
| [ENSG00000144909](http://www.ensembl.org/Homo_sapiens/Gene/Summary?db=core;g=ENSG00000144909) | [OSBPL11](http://www.ensembl.org/Homo_sapiens/Gene/Summary?db=core;g=ENSG00000144909) |
| [ENSG00000177565](http://www.ensembl.org/Homo_sapiens/Gene/Summary?db=core;g=ENSG00000177565) | [TBL1XR1](http://www.ensembl.org/Homo_sapiens/Gene/Summary?db=core;g=ENSG00000177565) |
| [ENSG00000075651](http://www.ensembl.org/Homo_sapiens/Gene/Summary?db=core;g=ENSG00000075651) | [PLD1](http://www.ensembl.org/Homo_sapiens/Gene/Summary?db=core;g=ENSG00000075651) |
| [ENSG00000154310](http://www.ensembl.org/Homo_sapiens/Gene/Summary?db=core;g=ENSG00000154310) | [TNIK](http://www.ensembl.org/Homo_sapiens/Gene/Summary?db=core;g=ENSG00000154310) |
| [ENSG00000160145](http://www.ensembl.org/Homo_sapiens/Gene/Summary?db=core;g=ENSG00000160145) | [KALRN](http://www.ensembl.org/Homo_sapiens/Gene/Summary?db=core;g=ENSG00000160145) |
| [ENSG00000075785](http://www.ensembl.org/Homo_sapiens/Gene/Summary?db=core;g=ENSG00000075785) | [RAB7A](http://www.ensembl.org/Homo_sapiens/Gene/Summary?db=core;g=ENSG00000075785) |
| [ENSG00000183833](http://www.ensembl.org/Homo_sapiens/Gene/Summary?db=core;g=ENSG00000183833) | [MAATS1](http://www.ensembl.org/Homo_sapiens/Gene/Summary?db=core;g=ENSG00000183833) |
| [ENSG00000145103](http://www.ensembl.org/Homo_sapiens/Gene/Summary?db=core;g=ENSG00000145103) | [ILDR1](http://www.ensembl.org/Homo_sapiens/Gene/Summary?db=core;g=ENSG00000145103) |
| [ENSG00000196981](http://www.ensembl.org/Homo_sapiens/Gene/Summary?db=core;g=ENSG00000196981) | [WDR5B](http://www.ensembl.org/Homo_sapiens/Gene/Summary?db=core;g=ENSG00000196981) |
| [ENSG00000169714](http://www.ensembl.org/Homo_sapiens/Gene/Summary?db=core;g=ENSG00000169714) | [CNBP](http://www.ensembl.org/Homo_sapiens/Gene/Summary?db=core;g=ENSG00000169714) |
| [ENSG00000163902](http://www.ensembl.org/Homo_sapiens/Gene/Summary?db=core;g=ENSG00000163902) | [RPN1](http://www.ensembl.org/Homo_sapiens/Gene/Summary?db=core;g=ENSG00000163902) |
| [ENSG00000145075](http://www.ensembl.org/Homo_sapiens/Gene/Summary?db=core;g=ENSG00000145075) | [CCDC39](http://www.ensembl.org/Homo_sapiens/Gene/Summary?db=core;g=ENSG00000145075) |
| [ENSG00000179407](http://www.ensembl.org/Homo_sapiens/Gene/Summary?db=core;g=ENSG00000179407) | [DNAJB8](http://www.ensembl.org/Homo_sapiens/Gene/Summary?db=core;g=ENSG00000179407) |
| [ENSG00000114544](http://www.ensembl.org/Homo_sapiens/Gene/Summary?db=core;g=ENSG00000114544) | [SLC41A3](http://www.ensembl.org/Homo_sapiens/Gene/Summary?db=core;g=ENSG00000114544) |
| [ENSG00000113924](http://www.ensembl.org/Homo_sapiens/Gene/Summary?db=core;g=ENSG00000113924) | [HGD](http://www.ensembl.org/Homo_sapiens/Gene/Summary?db=core;g=ENSG00000113924) |
| [ENSG00000144837](http://www.ensembl.org/Homo_sapiens/Gene/Summary?db=core;g=ENSG00000144837) | [PLA1A](http://www.ensembl.org/Homo_sapiens/Gene/Summary?db=core;g=ENSG00000144837) |
| [ENSG00000121879](http://www.ensembl.org/Homo_sapiens/Gene/Summary?db=core;g=ENSG00000121879) | [PIK3CA](http://www.ensembl.org/Homo_sapiens/Gene/Summary?db=core;g=ENSG00000121879) |
| [ENSG00000176142](http://www.ensembl.org/Homo_sapiens/Gene/Summary?db=core;g=ENSG00000176142) | [TMEM39A](http://www.ensembl.org/Homo_sapiens/Gene/Summary?db=core;g=ENSG00000176142) |
| [ENSG00000172780](http://www.ensembl.org/Homo_sapiens/Gene/Summary?db=core;g=ENSG00000172780) | [RAB43](http://www.ensembl.org/Homo_sapiens/Gene/Summary?db=core;g=ENSG00000172780) |
| [ENSG00000082781](http://www.ensembl.org/Homo_sapiens/Gene/Summary?db=core;g=ENSG00000082781) | [ITGB5](http://www.ensembl.org/Homo_sapiens/Gene/Summary?db=core;g=ENSG00000082781) |
| [ENSG00000132394](http://www.ensembl.org/Homo_sapiens/Gene/Summary?db=core;g=ENSG00000132394) | [EEFSEC](http://www.ensembl.org/Homo_sapiens/Gene/Summary?db=core;g=ENSG00000132394) |
| [ENSG00000114030](http://www.ensembl.org/Homo_sapiens/Gene/Summary?db=core;g=ENSG00000114030) | [KPNA1](http://www.ensembl.org/Homo_sapiens/Gene/Summary?db=core;g=ENSG00000114030) |
| [ENSG00000144852](http://www.ensembl.org/Homo_sapiens/Gene/Summary?db=core;g=ENSG00000144852) | [NR1I2](http://www.ensembl.org/Homo_sapiens/Gene/Summary?db=core;g=ENSG00000144852) |
| [ENSG00000163406](http://www.ensembl.org/Homo_sapiens/Gene/Summary?db=core;g=ENSG00000163406) | [SLC15A2](http://www.ensembl.org/Homo_sapiens/Gene/Summary?db=core;g=ENSG00000163406) |
| [ENSG00000163728](http://www.ensembl.org/Homo_sapiens/Gene/Summary?db=core;g=ENSG00000163728) | [TTC14](http://www.ensembl.org/Homo_sapiens/Gene/Summary?db=core;g=ENSG00000163728) |
| [ENSG00000179348](http://www.ensembl.org/Homo_sapiens/Gene/Summary?db=core;g=ENSG00000179348) | [GATA2](http://www.ensembl.org/Homo_sapiens/Gene/Summary?db=core;g=ENSG00000179348) |
| [ENSG00000240682](http://www.ensembl.org/Homo_sapiens/Gene/Summary?db=core;g=ENSG00000240682) | [ISY1](http://www.ensembl.org/Homo_sapiens/Gene/Summary?db=core;g=ENSG00000240682) |
| [ENSG00000070476](http://www.ensembl.org/Homo_sapiens/Gene/Summary?db=core;g=ENSG00000070476) | [ZXDC](http://www.ensembl.org/Homo_sapiens/Gene/Summary?db=core;g=ENSG00000070476) |
| [ENSG00000163840](http://www.ensembl.org/Homo_sapiens/Gene/Summary?db=core;g=ENSG00000163840) | [DTX3L](http://www.ensembl.org/Homo_sapiens/Gene/Summary?db=core;g=ENSG00000163840) |
| [ENSG00000169704](http://www.ensembl.org/Homo_sapiens/Gene/Summary?db=core;g=ENSG00000169704) | [GP9](http://www.ensembl.org/Homo_sapiens/Gene/Summary?db=core;g=ENSG00000169704) |
| [ENSG00000075420](http://www.ensembl.org/Homo_sapiens/Gene/Summary?db=core;g=ENSG00000075420) | [FNDC3B](http://www.ensembl.org/Homo_sapiens/Gene/Summary?db=core;g=ENSG00000075420) |
| [ENSG00000114554](http://www.ensembl.org/Homo_sapiens/Gene/Summary?db=core;g=ENSG00000114554) | [PLXNA1](http://www.ensembl.org/Homo_sapiens/Gene/Summary?db=core;g=ENSG00000114554) |
| [ENSG00000171121](http://www.ensembl.org/Homo_sapiens/Gene/Summary?db=core;g=ENSG00000171121) | [KCNMB3](http://www.ensembl.org/Homo_sapiens/Gene/Summary?db=core;g=ENSG00000171121) |
| [ENSG00000163584](http://www.ensembl.org/Homo_sapiens/Gene/Summary?db=core;g=ENSG00000163584) | [RPL22L1](http://www.ensembl.org/Homo_sapiens/Gene/Summary?db=core;g=ENSG00000163584) |
| [ENSG00000114013](http://www.ensembl.org/Homo_sapiens/Gene/Summary?db=core;g=ENSG00000114013) | [CD86](http://www.ensembl.org/Homo_sapiens/Gene/Summary?db=core;g=ENSG00000114013) |
| [ENSG00000175792](http://www.ensembl.org/Homo_sapiens/Gene/Summary?db=core;g=ENSG00000175792) | [RUVBL1](http://www.ensembl.org/Homo_sapiens/Gene/Summary?db=core;g=ENSG00000175792) |
| [ENSG00000065518](http://www.ensembl.org/Homo_sapiens/Gene/Summary?db=core;g=ENSG00000065518) | [NDUFB4](http://www.ensembl.org/Homo_sapiens/Gene/Summary?db=core;g=ENSG00000065518) |
| [ENSG00000058262](http://www.ensembl.org/Homo_sapiens/Gene/Summary?db=core;g=ENSG00000058262) | [SEC61A1](http://www.ensembl.org/Homo_sapiens/Gene/Summary?db=core;g=ENSG00000058262) |
| [ENSG00000138463](http://www.ensembl.org/Homo_sapiens/Gene/Summary?db=core;g=ENSG00000138463) | [DIRC2](http://www.ensembl.org/Homo_sapiens/Gene/Summary?db=core;g=ENSG00000138463) |
| [ENSG00000144840](http://www.ensembl.org/Homo_sapiens/Gene/Summary?db=core;g=ENSG00000144840) | [RABL3](http://www.ensembl.org/Homo_sapiens/Gene/Summary?db=core;g=ENSG00000144840) |
| [ENSG00000163913](http://www.ensembl.org/Homo_sapiens/Gene/Summary?db=core;g=ENSG00000163913) | [IFT122](http://www.ensembl.org/Homo_sapiens/Gene/Summary?db=core;g=ENSG00000163913) |
| [ENSG00000163430](http://www.ensembl.org/Homo_sapiens/Gene/Summary?db=core;g=ENSG00000163430) | [FSTL1](http://www.ensembl.org/Homo_sapiens/Gene/Summary?db=core;g=ENSG00000163430) |
| [ENSG00000180353](http://www.ensembl.org/Homo_sapiens/Gene/Summary?db=core;g=ENSG00000180353) | [HCLS1](http://www.ensembl.org/Homo_sapiens/Gene/Summary?db=core;g=ENSG00000180353) |
| [ENSG00000173230](http://www.ensembl.org/Homo_sapiens/Gene/Summary?db=core;g=ENSG00000173230) | [GOLGB1](http://www.ensembl.org/Homo_sapiens/Gene/Summary?db=core;g=ENSG00000173230) |
| [ENSG00000065371](http://www.ensembl.org/Homo_sapiens/Gene/Summary?db=core;g=ENSG00000065371) | [ROPN1](http://www.ensembl.org/Homo_sapiens/Gene/Summary?db=core;g=ENSG00000065371) |
| [ENSG00000163428](http://www.ensembl.org/Homo_sapiens/Gene/Summary?db=core;g=ENSG00000163428) | [LRRC58](http://www.ensembl.org/Homo_sapiens/Gene/Summary?db=core;g=ENSG00000163428) |
| [ENSG00000172667](http://www.ensembl.org/Homo_sapiens/Gene/Summary?db=core;g=ENSG00000172667) | [ZMAT3](http://www.ensembl.org/Homo_sapiens/Gene/Summary?db=core;g=ENSG00000172667) |
| [ENSG00000163389](http://www.ensembl.org/Homo_sapiens/Gene/Summary?db=core;g=ENSG00000163389) | [POGLUT1](http://www.ensembl.org/Homo_sapiens/Gene/Summary?db=core;g=ENSG00000163389) |
| [ENSG00000169760](http://www.ensembl.org/Homo_sapiens/Gene/Summary?db=core;g=ENSG00000169760) | [NLGN1](http://www.ensembl.org/Homo_sapiens/Gene/Summary?db=core;g=ENSG00000169760) |
| [ENSG00000114520](http://www.ensembl.org/Homo_sapiens/Gene/Summary?db=core;g=ENSG00000114520) | [SNX4](http://www.ensembl.org/Homo_sapiens/Gene/Summary?db=core;g=ENSG00000114520) |
| [ENSG00000173193](http://www.ensembl.org/Homo_sapiens/Gene/Summary?db=core;g=ENSG00000173193) | [PARP14](http://www.ensembl.org/Homo_sapiens/Gene/Summary?db=core;g=ENSG00000173193) |
| [ENSG00000065534](http://www.ensembl.org/Homo_sapiens/Gene/Summary?db=core;g=ENSG00000065534) | [MYLK](http://www.ensembl.org/Homo_sapiens/Gene/Summary?db=core;g=ENSG00000065534) |
| [ENSG00000171109](http://www.ensembl.org/Homo_sapiens/Gene/Summary?db=core;g=ENSG00000171109) | [MFN1](http://www.ensembl.org/Homo_sapiens/Gene/Summary?db=core;g=ENSG00000171109) |
| [ENSG00000163870](http://www.ensembl.org/Homo_sapiens/Gene/Summary?db=core;g=ENSG00000163870) | [TPRA1](http://www.ensembl.org/Homo_sapiens/Gene/Summary?db=core;g=ENSG00000163870) |
| [ENSG00000065485](http://www.ensembl.org/Homo_sapiens/Gene/Summary?db=core;g=ENSG00000065485) | [PDIA5](http://www.ensembl.org/Homo_sapiens/Gene/Summary?db=core;g=ENSG00000065485) |
| [ENSG00000197584](http://www.ensembl.org/Homo_sapiens/Gene/Summary?db=core;g=ENSG00000197584) | [KCNMB2](http://www.ensembl.org/Homo_sapiens/Gene/Summary?db=core;g=ENSG00000197584) |
| [ENSG00000160124](http://www.ensembl.org/Homo_sapiens/Gene/Summary?db=core;g=ENSG00000160124) | [CCDC58](http://www.ensembl.org/Homo_sapiens/Gene/Summary?db=core;g=ENSG00000160124) |
| [ENSG00000073111](http://www.ensembl.org/Homo_sapiens/Gene/Summary?db=core;g=ENSG00000073111) | [MCM2](http://www.ensembl.org/Homo_sapiens/Gene/Summary?db=core;g=ENSG00000073111) |
| [ENSG00000173175](http://www.ensembl.org/Homo_sapiens/Gene/Summary?db=core;g=ENSG00000173175) | [ADCY5](http://www.ensembl.org/Homo_sapiens/Gene/Summary?db=core;g=ENSG00000173175) |
| [ENSG00000159685](http://www.ensembl.org/Homo_sapiens/Gene/Summary?db=core;g=ENSG00000159685) | [CHCHD6](http://www.ensembl.org/Homo_sapiens/Gene/Summary?db=core;g=ENSG00000159685) |
| [ENSG00000082684](http://www.ensembl.org/Homo_sapiens/Gene/Summary?db=core;g=ENSG00000082684) | [SEMA5B](http://www.ensembl.org/Homo_sapiens/Gene/Summary?db=core;g=ENSG00000082684) |
| [ENSG00000153767](http://www.ensembl.org/Homo_sapiens/Gene/Summary?db=core;g=ENSG00000153767) | [GTF2E1](http://www.ensembl.org/Homo_sapiens/Gene/Summary?db=core;g=ENSG00000153767) |
| [ENSG00000187715](http://www.ensembl.org/Homo_sapiens/Gene/Summary?db=core;g=ENSG00000187715) | [KBTBD12](http://www.ensembl.org/Homo_sapiens/Gene/Summary?db=core;g=ENSG00000187715) |
| [ENSG00000163581](http://www.ensembl.org/Homo_sapiens/Gene/Summary?db=core;g=ENSG00000163581) | [SLC2A2](http://www.ensembl.org/Homo_sapiens/Gene/Summary?db=core;g=ENSG00000163581) |
| [ENSG00000173226](http://www.ensembl.org/Homo_sapiens/Gene/Summary?db=core;g=ENSG00000173226) | [IQCB1](http://www.ensembl.org/Homo_sapiens/Gene/Summary?db=core;g=ENSG00000173226) |
| [ENSG00000275163](http://www.ensembl.org/Homo_sapiens/Gene/Summary?db=core;g=ENSG00000275163) | [RP11-385J1.3](http://www.ensembl.org/Homo_sapiens/Gene/Summary?db=core;g=ENSG00000275163) |
| [ENSG00000136521](http://www.ensembl.org/Homo_sapiens/Gene/Summary?db=core;g=ENSG00000136521) | [NDUFB5](http://www.ensembl.org/Homo_sapiens/Gene/Summary?db=core;g=ENSG00000136521) |
| [ENSG00000163848](http://www.ensembl.org/Homo_sapiens/Gene/Summary?db=core;g=ENSG00000163848) | [ZNF148](http://www.ensembl.org/Homo_sapiens/Gene/Summary?db=core;g=ENSG00000163848) |
| [ENSG00000144962](http://www.ensembl.org/Homo_sapiens/Gene/Summary?db=core;g=ENSG00000144962) | [SPATA16](http://www.ensembl.org/Homo_sapiens/Gene/Summary?db=core;g=ENSG00000144962) |
| [ENSG00000144843](http://www.ensembl.org/Homo_sapiens/Gene/Summary?db=core;g=ENSG00000144843) | [ADPRH](http://www.ensembl.org/Homo_sapiens/Gene/Summary?db=core;g=ENSG00000144843) |
| [ENSG00000114450](http://www.ensembl.org/Homo_sapiens/Gene/Summary?db=core;g=ENSG00000114450) | [GNB4](http://www.ensembl.org/Homo_sapiens/Gene/Summary?db=core;g=ENSG00000114450) |
| [ENSG00000121552](http://www.ensembl.org/Homo_sapiens/Gene/Summary?db=core;g=ENSG00000121552) | [CSTA](http://www.ensembl.org/Homo_sapiens/Gene/Summary?db=core;g=ENSG00000121552) |
| [ENSG00000175455](http://www.ensembl.org/Homo_sapiens/Gene/Summary?db=core;g=ENSG00000175455) | [CCDC14](http://www.ensembl.org/Homo_sapiens/Gene/Summary?db=core;g=ENSG00000175455) |
| [ENSG00000145087](http://www.ensembl.org/Homo_sapiens/Gene/Summary?db=core;g=ENSG00000145087) | [STXBP5L](http://www.ensembl.org/Homo_sapiens/Gene/Summary?db=core;g=ENSG00000145087) |
| [ENSG00000173702](http://www.ensembl.org/Homo_sapiens/Gene/Summary?db=core;g=ENSG00000173702) | [MUC13](http://www.ensembl.org/Homo_sapiens/Gene/Summary?db=core;g=ENSG00000173702) |
| [ENSG00000177646](http://www.ensembl.org/Homo_sapiens/Gene/Summary?db=core;g=ENSG00000177646) | [ACAD9](http://www.ensembl.org/Homo_sapiens/Gene/Summary?db=core;g=ENSG00000177646) |
| [ENSG00000180767](http://www.ensembl.org/Homo_sapiens/Gene/Summary?db=core;g=ENSG00000180767) | [CHST13](http://www.ensembl.org/Homo_sapiens/Gene/Summary?db=core;g=ENSG00000180767) |
| [ENSG00000082701](http://www.ensembl.org/Homo_sapiens/Gene/Summary?db=core;g=ENSG00000082701) | [GSK3B](http://www.ensembl.org/Homo_sapiens/Gene/Summary?db=core;g=ENSG00000082701) |
| [ENSG00000121594](http://www.ensembl.org/Homo_sapiens/Gene/Summary?db=core;g=ENSG00000121594) | [CD80](http://www.ensembl.org/Homo_sapiens/Gene/Summary?db=core;g=ENSG00000121594) |
| [ENSG00000114757](http://www.ensembl.org/Homo_sapiens/Gene/Summary?db=core;g=ENSG00000114757) | [PEX5L](http://www.ensembl.org/Homo_sapiens/Gene/Summary?db=core;g=ENSG00000114757) |
| [ENSG00000159650](http://www.ensembl.org/Homo_sapiens/Gene/Summary?db=core;g=ENSG00000159650) | [UROC1](http://www.ensembl.org/Homo_sapiens/Gene/Summary?db=core;g=ENSG00000159650) |
| [ENSG00000121858](http://www.ensembl.org/Homo_sapiens/Gene/Summary?db=core;g=ENSG00000121858) | [TNFSF10](http://www.ensembl.org/Homo_sapiens/Gene/Summary?db=core;g=ENSG00000121858) |
| [ENSG00000177694](http://www.ensembl.org/Homo_sapiens/Gene/Summary?db=core;g=ENSG00000177694) | [NAALADL2](http://www.ensembl.org/Homo_sapiens/Gene/Summary?db=core;g=ENSG00000177694) |
| [ENSG00000163884](http://www.ensembl.org/Homo_sapiens/Gene/Summary?db=core;g=ENSG00000163884) | [KLF15](http://www.ensembl.org/Homo_sapiens/Gene/Summary?db=core;g=ENSG00000163884) |
| [ENSG00000121577](http://www.ensembl.org/Homo_sapiens/Gene/Summary?db=core;g=ENSG00000121577) | [POPDC2](http://www.ensembl.org/Homo_sapiens/Gene/Summary?db=core;g=ENSG00000121577) |
| [ENSG00000138495](http://www.ensembl.org/Homo_sapiens/Gene/Summary?db=core;g=ENSG00000138495) | [COX17](http://www.ensembl.org/Homo_sapiens/Gene/Summary?db=core;g=ENSG00000138495) |
| [ENSG00000173706](http://www.ensembl.org/Homo_sapiens/Gene/Summary?db=core;g=ENSG00000173706) | [HEG1](http://www.ensembl.org/Homo_sapiens/Gene/Summary?db=core;g=ENSG00000173706) |
| [ENSG00000113845](http://www.ensembl.org/Homo_sapiens/Gene/Summary?db=core;g=ENSG00000113845) | [TIMMDC1](http://www.ensembl.org/Homo_sapiens/Gene/Summary?db=core;g=ENSG00000113845) |
| [ENSG00000058056](http://www.ensembl.org/Homo_sapiens/Gene/Summary?db=core;g=ENSG00000058056) | [USP13](http://www.ensembl.org/Homo_sapiens/Gene/Summary?db=core;g=ENSG00000058056) |
| [ENSG00000114547](http://www.ensembl.org/Homo_sapiens/Gene/Summary?db=core;g=ENSG00000114547) | [ROPN1B](http://www.ensembl.org/Homo_sapiens/Gene/Summary?db=core;g=ENSG00000114547) |
| [ENSG00000121864](http://www.ensembl.org/Homo_sapiens/Gene/Summary?db=core;g=ENSG00000121864) | [ZNF639](http://www.ensembl.org/Homo_sapiens/Gene/Summary?db=core;g=ENSG00000121864) |
| [ENSG00000114631](http://www.ensembl.org/Homo_sapiens/Gene/Summary?db=core;g=ENSG00000114631) | [PODXL2](http://www.ensembl.org/Homo_sapiens/Gene/Summary?db=core;g=ENSG00000114631) |
| [ENSG00000172771](http://www.ensembl.org/Homo_sapiens/Gene/Summary?db=core;g=ENSG00000172771) | [EFCAB12](http://www.ensembl.org/Homo_sapiens/Gene/Summary?db=core;g=ENSG00000172771) |
| [ENSG00000184897](http://www.ensembl.org/Homo_sapiens/Gene/Summary?db=core;g=ENSG00000184897) | [H1FX](http://www.ensembl.org/Homo_sapiens/Gene/Summary?db=core;g=ENSG00000184897) |
| [ENSG00000114346](http://www.ensembl.org/Homo_sapiens/Gene/Summary?db=core;g=ENSG00000114346) | [ECT2](http://www.ensembl.org/Homo_sapiens/Gene/Summary?db=core;g=ENSG00000114346) |
| [ENSG00000144959](http://www.ensembl.org/Homo_sapiens/Gene/Summary?db=core;g=ENSG00000144959) | [NCEH1](http://www.ensembl.org/Homo_sapiens/Gene/Summary?db=core;g=ENSG00000144959) |
| [ENSG00000136522](http://www.ensembl.org/Homo_sapiens/Gene/Summary?db=core;g=ENSG00000136522) | [MRPL47](http://www.ensembl.org/Homo_sapiens/Gene/Summary?db=core;g=ENSG00000136522) |
| [ENSG00000189366](http://www.ensembl.org/Homo_sapiens/Gene/Summary?db=core;g=ENSG00000189366) | [ALG1L](http://www.ensembl.org/Homo_sapiens/Gene/Summary?db=core;g=ENSG00000189366) |
| [ENSG00000036828](http://www.ensembl.org/Homo_sapiens/Gene/Summary?db=core;g=ENSG00000036828) | [CASR](http://www.ensembl.org/Homo_sapiens/Gene/Summary?db=core;g=ENSG00000036828) |
| [ENSG00000197763](http://www.ensembl.org/Homo_sapiens/Gene/Summary?db=core;g=ENSG00000197763) | [TXNRD3](http://www.ensembl.org/Homo_sapiens/Gene/Summary?db=core;g=ENSG00000197763) |
| [ENSG00000169087](http://www.ensembl.org/Homo_sapiens/Gene/Summary?db=core;g=ENSG00000169087) | [HSPBAP1](http://www.ensembl.org/Homo_sapiens/Gene/Summary?db=core;g=ENSG00000169087) |
| [ENSG00000114491](http://www.ensembl.org/Homo_sapiens/Gene/Summary?db=core;g=ENSG00000114491) | [UMPS](http://www.ensembl.org/Homo_sapiens/Gene/Summary?db=core;g=ENSG00000114491) |
| [ENSG00000175697](http://www.ensembl.org/Homo_sapiens/Gene/Summary?db=core;g=ENSG00000175697) | [GPR156](http://www.ensembl.org/Homo_sapiens/Gene/Summary?db=core;g=ENSG00000175697) |
| [ENSG00000186329](http://www.ensembl.org/Homo_sapiens/Gene/Summary?db=core;g=ENSG00000186329) | [TMEM212](http://www.ensembl.org/Homo_sapiens/Gene/Summary?db=core;g=ENSG00000186329) |
| [ENSG00000163577](http://www.ensembl.org/Homo_sapiens/Gene/Summary?db=core;g=ENSG00000163577) | [EIF5A2](http://www.ensembl.org/Homo_sapiens/Gene/Summary?db=core;g=ENSG00000163577) |
| [ENSG00000183624](http://www.ensembl.org/Homo_sapiens/Gene/Summary?db=core;g=ENSG00000183624) | [HMCES](http://www.ensembl.org/Homo_sapiens/Gene/Summary?db=core;g=ENSG00000183624) |
| [ENSG00000013297](http://www.ensembl.org/Homo_sapiens/Gene/Summary?db=core;g=ENSG00000013297) | [CLDN11](http://www.ensembl.org/Homo_sapiens/Gene/Summary?db=core;g=ENSG00000013297) |
| [ENSG00000206527](http://www.ensembl.org/Homo_sapiens/Gene/Summary?db=core;g=ENSG00000206527) | [HACD2](http://www.ensembl.org/Homo_sapiens/Gene/Summary?db=core;g=ENSG00000206527) |
| [ENSG00000136518](http://www.ensembl.org/Homo_sapiens/Gene/Summary?db=core;g=ENSG00000136518) | [ACTL6A](http://www.ensembl.org/Homo_sapiens/Gene/Summary?db=core;g=ENSG00000136518) |
| [ENSG00000074416](http://www.ensembl.org/Homo_sapiens/Gene/Summary?db=core;g=ENSG00000074416) | [MGLL](http://www.ensembl.org/Homo_sapiens/Gene/Summary?db=core;g=ENSG00000074416) |
| [ENSG00000051341](http://www.ensembl.org/Homo_sapiens/Gene/Summary?db=core;g=ENSG00000051341) | [POLQ](http://www.ensembl.org/Homo_sapiens/Gene/Summary?db=core;g=ENSG00000051341) |
| [ENSG00000114626](http://www.ensembl.org/Homo_sapiens/Gene/Summary?db=core;g=ENSG00000114626) | [ABTB1](http://www.ensembl.org/Homo_sapiens/Gene/Summary?db=core;g=ENSG00000114626) |
| [ENSG00000121542](http://www.ensembl.org/Homo_sapiens/Gene/Summary?db=core;g=ENSG00000121542) | [SEC22A](http://www.ensembl.org/Homo_sapiens/Gene/Summary?db=core;g=ENSG00000121542) |
| [ENSG00000129071](http://www.ensembl.org/Homo_sapiens/Gene/Summary?db=core;g=ENSG00000129071) | [MBD4](http://www.ensembl.org/Homo_sapiens/Gene/Summary?db=core;g=ENSG00000129071) |
| [ENSG00000163833](http://www.ensembl.org/Homo_sapiens/Gene/Summary?db=core;g=ENSG00000163833) | [FBXO40](http://www.ensembl.org/Homo_sapiens/Gene/Summary?db=core;g=ENSG00000163833) |
| [ENSG00000173200](http://www.ensembl.org/Homo_sapiens/Gene/Summary?db=core;g=ENSG00000173200) | [PARP15](http://www.ensembl.org/Homo_sapiens/Gene/Summary?db=core;g=ENSG00000173200) |
| [ENSG00000114654](http://www.ensembl.org/Homo_sapiens/Gene/Summary?db=core;g=ENSG00000114654) | [EFCC1](http://www.ensembl.org/Homo_sapiens/Gene/Summary?db=core;g=ENSG00000114654) |
| [ENSG00000181789](http://www.ensembl.org/Homo_sapiens/Gene/Summary?db=core;g=ENSG00000181789) | [COPG1](http://www.ensembl.org/Homo_sapiens/Gene/Summary?db=core;g=ENSG00000181789) |
| [ENSG00000151612](http://www.ensembl.org/Homo_sapiens/Gene/Summary?db=core;g=ENSG00000151612) | [ZNF827](http://www.ensembl.org/Homo_sapiens/Gene/Summary?db=core;g=ENSG00000151612) |
| [ENSG00000185758](http://www.ensembl.org/Homo_sapiens/Gene/Summary?db=core;g=ENSG00000185758) | [CLDN24](http://www.ensembl.org/Homo_sapiens/Gene/Summary?db=core;g=ENSG00000185758) |
| [ENSG00000109654](http://www.ensembl.org/Homo_sapiens/Gene/Summary?db=core;g=ENSG00000109654) | [TRIM2](http://www.ensembl.org/Homo_sapiens/Gene/Summary?db=core;g=ENSG00000109654) |
| [ENSG00000179059](http://www.ensembl.org/Homo_sapiens/Gene/Summary?db=core;g=ENSG00000179059) | [ZFP42](http://www.ensembl.org/Homo_sapiens/Gene/Summary?db=core;g=ENSG00000179059) |
| [ENSG00000189099](http://www.ensembl.org/Homo_sapiens/Gene/Summary?db=core;g=ENSG00000189099) | [PRSS48](http://www.ensembl.org/Homo_sapiens/Gene/Summary?db=core;g=ENSG00000189099) |
| [ENSG00000272297](http://www.ensembl.org/Homo_sapiens/Gene/Summary?db=core;g=ENSG00000272297) | [RP11-215A19.2](http://www.ensembl.org/Homo_sapiens/Gene/Summary?db=core;g=ENSG00000272297) |
| [ENSG00000151615](http://www.ensembl.org/Homo_sapiens/Gene/Summary?db=core;g=ENSG00000151615) | [POU4F2](http://www.ensembl.org/Homo_sapiens/Gene/Summary?db=core;g=ENSG00000151615) |
| [ENSG00000184108](http://www.ensembl.org/Homo_sapiens/Gene/Summary?db=core;g=ENSG00000184108) | [TRIML1](http://www.ensembl.org/Homo_sapiens/Gene/Summary?db=core;g=ENSG00000184108) |
| [ENSG00000260596](http://www.ensembl.org/Homo_sapiens/Gene/Summary?db=core;g=ENSG00000260596) | [DUX4](http://www.ensembl.org/Homo_sapiens/Gene/Summary?db=core;g=ENSG00000260596) |
| [ENSG00000120519](http://www.ensembl.org/Homo_sapiens/Gene/Summary?db=core;g=ENSG00000120519) | [SLC10A7](http://www.ensembl.org/Homo_sapiens/Gene/Summary?db=core;g=ENSG00000120519) |
| [ENSG00000088926](http://www.ensembl.org/Homo_sapiens/Gene/Summary?db=core;g=ENSG00000088926) | [F11](http://www.ensembl.org/Homo_sapiens/Gene/Summary?db=core;g=ENSG00000088926) |
| [ENSG00000164306](http://www.ensembl.org/Homo_sapiens/Gene/Summary?db=core;g=ENSG00000164306) | [PRIMPOL](http://www.ensembl.org/Homo_sapiens/Gene/Summary?db=core;g=ENSG00000164306) |
| [ENSG00000153147](http://www.ensembl.org/Homo_sapiens/Gene/Summary?db=core;g=ENSG00000153147) | [SMARCA5](http://www.ensembl.org/Homo_sapiens/Gene/Summary?db=core;g=ENSG00000153147) |
| [ENSG00000083857](http://www.ensembl.org/Homo_sapiens/Gene/Summary?db=core;g=ENSG00000083857) | [FAT1](http://www.ensembl.org/Homo_sapiens/Gene/Summary?db=core;g=ENSG00000083857) |
| [ENSG00000151726](http://www.ensembl.org/Homo_sapiens/Gene/Summary?db=core;g=ENSG00000151726) | [ACSL1](http://www.ensembl.org/Homo_sapiens/Gene/Summary?db=core;g=ENSG00000151726) |
| [ENSG00000164161](http://www.ensembl.org/Homo_sapiens/Gene/Summary?db=core;g=ENSG00000164161) | [HHIP](http://www.ensembl.org/Homo_sapiens/Gene/Summary?db=core;g=ENSG00000164161) |
| [ENSG00000168412](http://www.ensembl.org/Homo_sapiens/Gene/Summary?db=core;g=ENSG00000168412) | [MTNR1A](http://www.ensembl.org/Homo_sapiens/Gene/Summary?db=core;g=ENSG00000168412) |
| [ENSG00000164169](http://www.ensembl.org/Homo_sapiens/Gene/Summary?db=core;g=ENSG00000164169) | [PRMT9](http://www.ensembl.org/Homo_sapiens/Gene/Summary?db=core;g=ENSG00000164169) |
| [ENSG00000168556](http://www.ensembl.org/Homo_sapiens/Gene/Summary?db=core;g=ENSG00000168556) | [ING2](http://www.ensembl.org/Homo_sapiens/Gene/Summary?db=core;g=ENSG00000168556) |
| [ENSG00000137473](http://www.ensembl.org/Homo_sapiens/Gene/Summary?db=core;g=ENSG00000137473) | [TTC29](http://www.ensembl.org/Homo_sapiens/Gene/Summary?db=core;g=ENSG00000137473) |
| [ENSG00000109670](http://www.ensembl.org/Homo_sapiens/Gene/Summary?db=core;g=ENSG00000109670) | [FBXW7](http://www.ensembl.org/Homo_sapiens/Gene/Summary?db=core;g=ENSG00000109670) |
| [ENSG00000170185](http://www.ensembl.org/Homo_sapiens/Gene/Summary?db=core;g=ENSG00000170185) | [USP38](http://www.ensembl.org/Homo_sapiens/Gene/Summary?db=core;g=ENSG00000170185) |
| [ENSG00000164162](http://www.ensembl.org/Homo_sapiens/Gene/Summary?db=core;g=ENSG00000164162) | [ANAPC10](http://www.ensembl.org/Homo_sapiens/Gene/Summary?db=core;g=ENSG00000164162) |
| [ENSG00000170365](http://www.ensembl.org/Homo_sapiens/Gene/Summary?db=core;g=ENSG00000170365) | [SMAD1](http://www.ensembl.org/Homo_sapiens/Gene/Summary?db=core;g=ENSG00000170365) |
| [ENSG00000168538](http://www.ensembl.org/Homo_sapiens/Gene/Summary?db=core;g=ENSG00000168538) | [TRAPPC11](http://www.ensembl.org/Homo_sapiens/Gene/Summary?db=core;g=ENSG00000168538) |
| [ENSG00000197465](http://www.ensembl.org/Homo_sapiens/Gene/Summary?db=core;g=ENSG00000197465) | [GYPE](http://www.ensembl.org/Homo_sapiens/Gene/Summary?db=core;g=ENSG00000197465) |
| [ENSG00000181541](http://www.ensembl.org/Homo_sapiens/Gene/Summary?db=core;g=ENSG00000181541) | [MAB21L2](http://www.ensembl.org/Homo_sapiens/Gene/Summary?db=core;g=ENSG00000181541) |
| [ENSG00000173320](http://www.ensembl.org/Homo_sapiens/Gene/Summary?db=core;g=ENSG00000173320) | [STOX2](http://www.ensembl.org/Homo_sapiens/Gene/Summary?db=core;g=ENSG00000173320) |
| [ENSG00000164303](http://www.ensembl.org/Homo_sapiens/Gene/Summary?db=core;g=ENSG00000164303) | [ENPP6](http://www.ensembl.org/Homo_sapiens/Gene/Summary?db=core;g=ENSG00000164303) |
| [ENSG00000164168](http://www.ensembl.org/Homo_sapiens/Gene/Summary?db=core;g=ENSG00000164168) | [TMEM184C](http://www.ensembl.org/Homo_sapiens/Gene/Summary?db=core;g=ENSG00000164168) |
| [ENSG00000164342](http://www.ensembl.org/Homo_sapiens/Gene/Summary?db=core;g=ENSG00000164342) | [TLR3](http://www.ensembl.org/Homo_sapiens/Gene/Summary?db=core;g=ENSG00000164342) |
| [ENSG00000218336](http://www.ensembl.org/Homo_sapiens/Gene/Summary?db=core;g=ENSG00000218336) | [TENM3](http://www.ensembl.org/Homo_sapiens/Gene/Summary?db=core;g=ENSG00000218336) |
| [ENSG00000198589](http://www.ensembl.org/Homo_sapiens/Gene/Summary?db=core;g=ENSG00000198589) | [LRBA](http://www.ensembl.org/Homo_sapiens/Gene/Summary?db=core;g=ENSG00000198589) |
| [ENSG00000168491](http://www.ensembl.org/Homo_sapiens/Gene/Summary?db=core;g=ENSG00000168491) | [CCDC110](http://www.ensembl.org/Homo_sapiens/Gene/Summary?db=core;g=ENSG00000168491) |
| [ENSG00000205129](http://www.ensembl.org/Homo_sapiens/Gene/Summary?db=core;g=ENSG00000205129) | [C4orf47](http://www.ensembl.org/Homo_sapiens/Gene/Summary?db=core;g=ENSG00000205129) |
| [ENSG00000164305](http://www.ensembl.org/Homo_sapiens/Gene/Summary?db=core;g=ENSG00000164305) | [CASP3](http://www.ensembl.org/Homo_sapiens/Gene/Summary?db=core;g=ENSG00000164305) |
| [ENSG00000168310](http://www.ensembl.org/Homo_sapiens/Gene/Summary?db=core;g=ENSG00000168310) | [IRF2](http://www.ensembl.org/Homo_sapiens/Gene/Summary?db=core;g=ENSG00000168310) |
| [ENSG00000154553](http://www.ensembl.org/Homo_sapiens/Gene/Summary?db=core;g=ENSG00000154553) | [PDLIM3](http://www.ensembl.org/Homo_sapiens/Gene/Summary?db=core;g=ENSG00000154553) |
| [ENSG00000164164](http://www.ensembl.org/Homo_sapiens/Gene/Summary?db=core;g=ENSG00000164164) | [OTUD4](http://www.ensembl.org/Homo_sapiens/Gene/Summary?db=core;g=ENSG00000164164) |
| [ENSG00000187821](http://www.ensembl.org/Homo_sapiens/Gene/Summary?db=core;g=ENSG00000187821) | [HELT](http://www.ensembl.org/Homo_sapiens/Gene/Summary?db=core;g=ENSG00000187821) |
| [ENSG00000151725](http://www.ensembl.org/Homo_sapiens/Gene/Summary?db=core;g=ENSG00000151725) | [CENPU](http://www.ensembl.org/Homo_sapiens/Gene/Summary?db=core;g=ENSG00000151725) |
| [ENSG00000109762](http://www.ensembl.org/Homo_sapiens/Gene/Summary?db=core;g=ENSG00000109762) | [SNX25](http://www.ensembl.org/Homo_sapiens/Gene/Summary?db=core;g=ENSG00000109762) |
| [ENSG00000170006](http://www.ensembl.org/Homo_sapiens/Gene/Summary?db=core;g=ENSG00000170006) | [TMEM154](http://www.ensembl.org/Homo_sapiens/Gene/Summary?db=core;g=ENSG00000170006) |
| [ENSG00000145476](http://www.ensembl.org/Homo_sapiens/Gene/Summary?db=core;g=ENSG00000145476) | [CYP4V2](http://www.ensembl.org/Homo_sapiens/Gene/Summary?db=core;g=ENSG00000145476) |
| [ENSG00000250361](http://www.ensembl.org/Homo_sapiens/Gene/Summary?db=core;g=ENSG00000250361) | [GYPB](http://www.ensembl.org/Homo_sapiens/Gene/Summary?db=core;g=ENSG00000250361) |
| [ENSG00000205097](http://www.ensembl.org/Homo_sapiens/Gene/Summary?db=core;g=ENSG00000205097) | [FRG2](http://www.ensembl.org/Homo_sapiens/Gene/Summary?db=core;g=ENSG00000205097) |
| [ENSG00000164344](http://www.ensembl.org/Homo_sapiens/Gene/Summary?db=core;g=ENSG00000164344) | [KLKB1](http://www.ensembl.org/Homo_sapiens/Gene/Summary?db=core;g=ENSG00000164344) |
| [ENSG00000154556](http://www.ensembl.org/Homo_sapiens/Gene/Summary?db=core;g=ENSG00000154556) | [SORBS2](http://www.ensembl.org/Homo_sapiens/Gene/Summary?db=core;g=ENSG00000154556) |
| [ENSG00000151611](http://www.ensembl.org/Homo_sapiens/Gene/Summary?db=core;g=ENSG00000151611) | [MMAA](http://www.ensembl.org/Homo_sapiens/Gene/Summary?db=core;g=ENSG00000151611) |
| [ENSG00000151729](http://www.ensembl.org/Homo_sapiens/Gene/Summary?db=core;g=ENSG00000151729) | [SLC25A4](http://www.ensembl.org/Homo_sapiens/Gene/Summary?db=core;g=ENSG00000151729) |
| [ENSG00000179046](http://www.ensembl.org/Homo_sapiens/Gene/Summary?db=core;g=ENSG00000179046) | [TRIML2](http://www.ensembl.org/Homo_sapiens/Gene/Summary?db=core;g=ENSG00000179046) |
| [ENSG00000186352](http://www.ensembl.org/Homo_sapiens/Gene/Summary?db=core;g=ENSG00000186352) | [ANKRD37](http://www.ensembl.org/Homo_sapiens/Gene/Summary?db=core;g=ENSG00000186352) |
| [ENSG00000071205](http://www.ensembl.org/Homo_sapiens/Gene/Summary?db=core;g=ENSG00000071205) | [ARHGAP10](http://www.ensembl.org/Homo_sapiens/Gene/Summary?db=core;g=ENSG00000071205) |
| [ENSG00000145425](http://www.ensembl.org/Homo_sapiens/Gene/Summary?db=core;g=ENSG00000145425) | [RPS3A](http://www.ensembl.org/Homo_sapiens/Gene/Summary?db=core;g=ENSG00000145425) |
| [ENSG00000164167](http://www.ensembl.org/Homo_sapiens/Gene/Summary?db=core;g=ENSG00000164167) | [LSM6](http://www.ensembl.org/Homo_sapiens/Gene/Summary?db=core;g=ENSG00000164167) |
| [ENSG00000151617](http://www.ensembl.org/Homo_sapiens/Gene/Summary?db=core;g=ENSG00000151617) | [EDNRA](http://www.ensembl.org/Homo_sapiens/Gene/Summary?db=core;g=ENSG00000151617) |
| [ENSG00000109775](http://www.ensembl.org/Homo_sapiens/Gene/Summary?db=core;g=ENSG00000109775) | [UFSP2](http://www.ensembl.org/Homo_sapiens/Gene/Summary?db=core;g=ENSG00000109775) |
| [ENSG00000109536](http://www.ensembl.org/Homo_sapiens/Gene/Summary?db=core;g=ENSG00000109536) | [FRG1](http://www.ensembl.org/Homo_sapiens/Gene/Summary?db=core;g=ENSG00000109536) |
| [ENSG00000164163](http://www.ensembl.org/Homo_sapiens/Gene/Summary?db=core;g=ENSG00000164163) | [ABCE1](http://www.ensembl.org/Homo_sapiens/Gene/Summary?db=core;g=ENSG00000164163) |
| [ENSG00000109458](http://www.ensembl.org/Homo_sapiens/Gene/Summary?db=core;g=ENSG00000109458) | [GAB1](http://www.ensembl.org/Homo_sapiens/Gene/Summary?db=core;g=ENSG00000109458) |
| [ENSG00000170180](http://www.ensembl.org/Homo_sapiens/Gene/Summary?db=core;g=ENSG00000170180) | [GYPA](http://www.ensembl.org/Homo_sapiens/Gene/Summary?db=core;g=ENSG00000170180) |
| [ENSG00000182552](http://www.ensembl.org/Homo_sapiens/Gene/Summary?db=core;g=ENSG00000182552) | [RWDD4](http://www.ensembl.org/Homo_sapiens/Gene/Summary?db=core;g=ENSG00000182552) |
| [ENSG00000109771](http://www.ensembl.org/Homo_sapiens/Gene/Summary?db=core;g=ENSG00000109771) | [LRP2BP](http://www.ensembl.org/Homo_sapiens/Gene/Summary?db=core;g=ENSG00000109771) |
| [ENSG00000151623](http://www.ensembl.org/Homo_sapiens/Gene/Summary?db=core;g=ENSG00000151623) | [NR3C2](http://www.ensembl.org/Homo_sapiens/Gene/Summary?db=core;g=ENSG00000151623) |
| [ENSG00000183090](http://www.ensembl.org/Homo_sapiens/Gene/Summary?db=core;g=ENSG00000183090) | [FREM3](http://www.ensembl.org/Homo_sapiens/Gene/Summary?db=core;g=ENSG00000183090) |
| [ENSG00000109686](http://www.ensembl.org/Homo_sapiens/Gene/Summary?db=core;g=ENSG00000109686) | [SH3D19](http://www.ensembl.org/Homo_sapiens/Gene/Summary?db=core;g=ENSG00000109686) |
| [ENSG00000177300](http://www.ensembl.org/Homo_sapiens/Gene/Summary?db=core;g=ENSG00000177300) | [CLDN22](http://www.ensembl.org/Homo_sapiens/Gene/Summary?db=core;g=ENSG00000177300) |
| [ENSG00000059691](http://www.ensembl.org/Homo_sapiens/Gene/Summary?db=core;g=ENSG00000059691) | [GATB](http://www.ensembl.org/Homo_sapiens/Gene/Summary?db=core;g=ENSG00000059691) |
| [ENSG00000151718](http://www.ensembl.org/Homo_sapiens/Gene/Summary?db=core;g=ENSG00000151718) | [WWC2](http://www.ensembl.org/Homo_sapiens/Gene/Summary?db=core;g=ENSG00000151718) |
| [ENSG00000170390](http://www.ensembl.org/Homo_sapiens/Gene/Summary?db=core;g=ENSG00000170390) | [DCLK2](http://www.ensembl.org/Homo_sapiens/Gene/Summary?db=core;g=ENSG00000170390) |
| [ENSG00000129187](http://www.ensembl.org/Homo_sapiens/Gene/Summary?db=core;g=ENSG00000129187) | [DCTD](http://www.ensembl.org/Homo_sapiens/Gene/Summary?db=core;g=ENSG00000129187) |
| [ENSG00000168564](http://www.ensembl.org/Homo_sapiens/Gene/Summary?db=core;g=ENSG00000168564) | [CDKN2AIP](http://www.ensembl.org/Homo_sapiens/Gene/Summary?db=core;g=ENSG00000168564) |
| [ENSG00000169989](http://www.ensembl.org/Homo_sapiens/Gene/Summary?db=core;g=ENSG00000169989) | [TIGD4](http://www.ensembl.org/Homo_sapiens/Gene/Summary?db=core;g=ENSG00000169989) |
| [ENSG00000164144](http://www.ensembl.org/Homo_sapiens/Gene/Summary?db=core;g=ENSG00000164144) | [ARFIP1](http://www.ensembl.org/Homo_sapiens/Gene/Summary?db=core;g=ENSG00000164144) |
| [ENSG00000148335](http://www.ensembl.org/Homo_sapiens/Gene/Summary?db=core;g=ENSG00000148335) | [NTMT1](http://www.ensembl.org/Homo_sapiens/Gene/Summary?db=core;g=ENSG00000148335) |
| [ENSG00000167081](http://www.ensembl.org/Homo_sapiens/Gene/Summary?db=core;g=ENSG00000167081) | [PBX3](http://www.ensembl.org/Homo_sapiens/Gene/Summary?db=core;g=ENSG00000167081) |
| [ENSG00000119421](http://www.ensembl.org/Homo_sapiens/Gene/Summary?db=core;g=ENSG00000119421) | [NDUFA8](http://www.ensembl.org/Homo_sapiens/Gene/Summary?db=core;g=ENSG00000119421) |
| [ENSG00000136827](http://www.ensembl.org/Homo_sapiens/Gene/Summary?db=core;g=ENSG00000136827) | [TOR1A](http://www.ensembl.org/Homo_sapiens/Gene/Summary?db=core;g=ENSG00000136827) |
| [ENSG00000167157](http://www.ensembl.org/Homo_sapiens/Gene/Summary?db=core;g=ENSG00000167157) | [PRRX2](http://www.ensembl.org/Homo_sapiens/Gene/Summary?db=core;g=ENSG00000167157) |
| [ENSG00000119397](http://www.ensembl.org/Homo_sapiens/Gene/Summary?db=core;g=ENSG00000119397) | [CNTRL](http://www.ensembl.org/Homo_sapiens/Gene/Summary?db=core;g=ENSG00000119397) |
| [ENSG00000136861](http://www.ensembl.org/Homo_sapiens/Gene/Summary?db=core;g=ENSG00000136861) | [CDK5RAP2](http://www.ensembl.org/Homo_sapiens/Gene/Summary?db=core;g=ENSG00000136861) |
| [ENSG00000056558](http://www.ensembl.org/Homo_sapiens/Gene/Summary?db=core;g=ENSG00000056558) | [TRAF1](http://www.ensembl.org/Homo_sapiens/Gene/Summary?db=core;g=ENSG00000056558) |
| [ENSG00000136848](http://www.ensembl.org/Homo_sapiens/Gene/Summary?db=core;g=ENSG00000136848) | [DAB2IP](http://www.ensembl.org/Homo_sapiens/Gene/Summary?db=core;g=ENSG00000136848) |
| [ENSG00000136811](http://www.ensembl.org/Homo_sapiens/Gene/Summary?db=core;g=ENSG00000136811) | [ODF2](http://www.ensembl.org/Homo_sapiens/Gene/Summary?db=core;g=ENSG00000136811) |
| [ENSG00000148358](http://www.ensembl.org/Homo_sapiens/Gene/Summary?db=core;g=ENSG00000148358) | [GPR107](http://www.ensembl.org/Homo_sapiens/Gene/Summary?db=core;g=ENSG00000148358) |
| [ENSG00000136816](http://www.ensembl.org/Homo_sapiens/Gene/Summary?db=core;g=ENSG00000136816) | [TOR1B](http://www.ensembl.org/Homo_sapiens/Gene/Summary?db=core;g=ENSG00000136816) |
| [ENSG00000148331](http://www.ensembl.org/Homo_sapiens/Gene/Summary?db=core;g=ENSG00000148331) | [ASB6](http://www.ensembl.org/Homo_sapiens/Gene/Summary?db=core;g=ENSG00000148331) |
| [ENSG00000167123](http://www.ensembl.org/Homo_sapiens/Gene/Summary?db=core;g=ENSG00000167123) | [CERCAM](http://www.ensembl.org/Homo_sapiens/Gene/Summary?db=core;g=ENSG00000167123) |
| [ENSG00000011454](http://www.ensembl.org/Homo_sapiens/Gene/Summary?db=core;g=ENSG00000011454) | [RABGAP1](http://www.ensembl.org/Homo_sapiens/Gene/Summary?db=core;g=ENSG00000011454) |
| [ENSG00000136933](http://www.ensembl.org/Homo_sapiens/Gene/Summary?db=core;g=ENSG00000136933) | [RABEPK](http://www.ensembl.org/Homo_sapiens/Gene/Summary?db=core;g=ENSG00000136933) |
| [ENSG00000148356](http://www.ensembl.org/Homo_sapiens/Gene/Summary?db=core;g=ENSG00000148356) | [LRSAM1](http://www.ensembl.org/Homo_sapiens/Gene/Summary?db=core;g=ENSG00000148356) |
| [ENSG00000148344](http://www.ensembl.org/Homo_sapiens/Gene/Summary?db=core;g=ENSG00000148344) | [PTGES](http://www.ensembl.org/Homo_sapiens/Gene/Summary?db=core;g=ENSG00000148344) |
| [ENSG00000188394](http://www.ensembl.org/Homo_sapiens/Gene/Summary?db=core;g=ENSG00000188394) | [GPR21](http://www.ensembl.org/Homo_sapiens/Gene/Summary?db=core;g=ENSG00000188394) |
| [ENSG00000119402](http://www.ensembl.org/Homo_sapiens/Gene/Summary?db=core;g=ENSG00000119402) | [FBXW2](http://www.ensembl.org/Homo_sapiens/Gene/Summary?db=core;g=ENSG00000119402) |
| [ENSG00000119335](http://www.ensembl.org/Homo_sapiens/Gene/Summary?db=core;g=ENSG00000119335) | [SET](http://www.ensembl.org/Homo_sapiens/Gene/Summary?db=core;g=ENSG00000119335) |
| [ENSG00000136930](http://www.ensembl.org/Homo_sapiens/Gene/Summary?db=core;g=ENSG00000136930) | [PSMB7](http://www.ensembl.org/Homo_sapiens/Gene/Summary?db=core;g=ENSG00000136930) |
| [ENSG00000197958](http://www.ensembl.org/Homo_sapiens/Gene/Summary?db=core;g=ENSG00000197958) | [RPL12](http://www.ensembl.org/Homo_sapiens/Gene/Summary?db=core;g=ENSG00000197958) |
| [ENSG00000136935](http://www.ensembl.org/Homo_sapiens/Gene/Summary?db=core;g=ENSG00000136935) | [GOLGA1](http://www.ensembl.org/Homo_sapiens/Gene/Summary?db=core;g=ENSG00000136935) |
| [ENSG00000173611](http://www.ensembl.org/Homo_sapiens/Gene/Summary?db=core;g=ENSG00000173611) | [SCAI](http://www.ensembl.org/Homo_sapiens/Gene/Summary?db=core;g=ENSG00000173611) |
| [ENSG00000136807](http://www.ensembl.org/Homo_sapiens/Gene/Summary?db=core;g=ENSG00000136807) | [CDK9](http://www.ensembl.org/Homo_sapiens/Gene/Summary?db=core;g=ENSG00000136807) |
| [ENSG00000180264](http://www.ensembl.org/Homo_sapiens/Gene/Summary?db=core;g=ENSG00000180264) | [ADGRD2](http://www.ensembl.org/Homo_sapiens/Gene/Summary?db=core;g=ENSG00000180264) |
| [ENSG00000095303](http://www.ensembl.org/Homo_sapiens/Gene/Summary?db=core;g=ENSG00000095303) | [PTGS1](http://www.ensembl.org/Homo_sapiens/Gene/Summary?db=core;g=ENSG00000095303) |
| [ENSG00000044574](http://www.ensembl.org/Homo_sapiens/Gene/Summary?db=core;g=ENSG00000044574) | [HSPA5](http://www.ensembl.org/Homo_sapiens/Gene/Summary?db=core;g=ENSG00000044574) |
| [ENSG00000136859](http://www.ensembl.org/Homo_sapiens/Gene/Summary?db=core;g=ENSG00000136859) | [ANGPTL2](http://www.ensembl.org/Homo_sapiens/Gene/Summary?db=core;g=ENSG00000136859) |
| [ENSG00000175287](http://www.ensembl.org/Homo_sapiens/Gene/Summary?db=core;g=ENSG00000175287) | [PHYHD1](http://www.ensembl.org/Homo_sapiens/Gene/Summary?db=core;g=ENSG00000175287) |
| [ENSG00000136828](http://www.ensembl.org/Homo_sapiens/Gene/Summary?db=core;g=ENSG00000136828) | [RALGPS1](http://www.ensembl.org/Homo_sapiens/Gene/Summary?db=core;g=ENSG00000136828) |
| [ENSG00000187239](http://www.ensembl.org/Homo_sapiens/Gene/Summary?db=core;g=ENSG00000187239) | [FNBP1](http://www.ensembl.org/Homo_sapiens/Gene/Summary?db=core;g=ENSG00000187239) |
| [ENSG00000148187](http://www.ensembl.org/Homo_sapiens/Gene/Summary?db=core;g=ENSG00000148187) | [MRRF](http://www.ensembl.org/Homo_sapiens/Gene/Summary?db=core;g=ENSG00000148187) |
| [ENSG00000171169](http://www.ensembl.org/Homo_sapiens/Gene/Summary?db=core;g=ENSG00000171169) | [NAIF1](http://www.ensembl.org/Homo_sapiens/Gene/Summary?db=core;g=ENSG00000171169) |
| [ENSG00000197694](http://www.ensembl.org/Homo_sapiens/Gene/Summary?db=core;g=ENSG00000197694) | [SPTAN1](http://www.ensembl.org/Homo_sapiens/Gene/Summary?db=core;g=ENSG00000197694) |
| [ENSG00000196152](http://www.ensembl.org/Homo_sapiens/Gene/Summary?db=core;g=ENSG00000196152) | [ZNF79](http://www.ensembl.org/Homo_sapiens/Gene/Summary?db=core;g=ENSG00000196152) |
| [ENSG00000106991](http://www.ensembl.org/Homo_sapiens/Gene/Summary?db=core;g=ENSG00000106991) | [ENG](http://www.ensembl.org/Homo_sapiens/Gene/Summary?db=core;g=ENSG00000106991) |
| [ENSG00000148341](http://www.ensembl.org/Homo_sapiens/Gene/Summary?db=core;g=ENSG00000148341) | [SH3GLB2](http://www.ensembl.org/Homo_sapiens/Gene/Summary?db=core;g=ENSG00000148341) |
| [ENSG00000095370](http://www.ensembl.org/Homo_sapiens/Gene/Summary?db=core;g=ENSG00000095370) | [SH2D3C](http://www.ensembl.org/Homo_sapiens/Gene/Summary?db=core;g=ENSG00000095370) |
| [ENSG00000119396](http://www.ensembl.org/Homo_sapiens/Gene/Summary?db=core;g=ENSG00000119396) | [RAB14](http://www.ensembl.org/Homo_sapiens/Gene/Summary?db=core;g=ENSG00000119396) |
| [ENSG00000167103](http://www.ensembl.org/Homo_sapiens/Gene/Summary?db=core;g=ENSG00000167103) | [PIP5KL1](http://www.ensembl.org/Homo_sapiens/Gene/Summary?db=core;g=ENSG00000167103) |
| [ENSG00000148339](http://www.ensembl.org/Homo_sapiens/Gene/Summary?db=core;g=ENSG00000148339) | [SLC25A25](http://www.ensembl.org/Homo_sapiens/Gene/Summary?db=core;g=ENSG00000148339) |
| [ENSG00000198917](http://www.ensembl.org/Homo_sapiens/Gene/Summary?db=core;g=ENSG00000198917) | [C9orf114](http://www.ensembl.org/Homo_sapiens/Gene/Summary?db=core;g=ENSG00000198917) |
| [ENSG00000095321](http://www.ensembl.org/Homo_sapiens/Gene/Summary?db=core;g=ENSG00000095321) | [CRAT](http://www.ensembl.org/Homo_sapiens/Gene/Summary?db=core;g=ENSG00000095321) |
| [ENSG00000136940](http://www.ensembl.org/Homo_sapiens/Gene/Summary?db=core;g=ENSG00000136940) | [PDCL](http://www.ensembl.org/Homo_sapiens/Gene/Summary?db=core;g=ENSG00000136940) |
| [ENSG00000106780](http://www.ensembl.org/Homo_sapiens/Gene/Summary?db=core;g=ENSG00000106780) | [MEGF9](http://www.ensembl.org/Homo_sapiens/Gene/Summary?db=core;g=ENSG00000106780) |
| [ENSG00000106804](http://www.ensembl.org/Homo_sapiens/Gene/Summary?db=core;g=ENSG00000106804) | [C5](http://www.ensembl.org/Homo_sapiens/Gene/Summary?db=core;g=ENSG00000106804) |
| [ENSG00000175283](http://www.ensembl.org/Homo_sapiens/Gene/Summary?db=core;g=ENSG00000175283) | [DOLK](http://www.ensembl.org/Homo_sapiens/Gene/Summary?db=core;g=ENSG00000175283) |
| [ENSG00000167118](http://www.ensembl.org/Homo_sapiens/Gene/Summary?db=core;g=ENSG00000167118) | [URM1](http://www.ensembl.org/Homo_sapiens/Gene/Summary?db=core;g=ENSG00000167118) |
| [ENSG00000167136](http://www.ensembl.org/Homo_sapiens/Gene/Summary?db=core;g=ENSG00000167136) | [ENDOG](http://www.ensembl.org/Homo_sapiens/Gene/Summary?db=core;g=ENSG00000167136) |
| [ENSG00000136856](http://www.ensembl.org/Homo_sapiens/Gene/Summary?db=core;g=ENSG00000136856) | [SLC2A8](http://www.ensembl.org/Homo_sapiens/Gene/Summary?db=core;g=ENSG00000136856) |
| [ENSG00000119446](http://www.ensembl.org/Homo_sapiens/Gene/Summary?db=core;g=ENSG00000119446) | [RBM18](http://www.ensembl.org/Homo_sapiens/Gene/Summary?db=core;g=ENSG00000119446) |
| [ENSG00000160408](http://www.ensembl.org/Homo_sapiens/Gene/Summary?db=core;g=ENSG00000160408) | [ST6GALNAC6](http://www.ensembl.org/Homo_sapiens/Gene/Summary?db=core;g=ENSG00000160408) |
| [ENSG00000165204](http://www.ensembl.org/Homo_sapiens/Gene/Summary?db=core;g=ENSG00000165204) | [OR1K1](http://www.ensembl.org/Homo_sapiens/Gene/Summary?db=core;g=ENSG00000165204) |
| [ENSG00000119383](http://www.ensembl.org/Homo_sapiens/Gene/Summary?db=core;g=ENSG00000119383) | [PPP2R4](http://www.ensembl.org/Homo_sapiens/Gene/Summary?db=core;g=ENSG00000119383) |
| [ENSG00000136950](http://www.ensembl.org/Homo_sapiens/Gene/Summary?db=core;g=ENSG00000136950) | [ARPC5L](http://www.ensembl.org/Homo_sapiens/Gene/Summary?db=core;g=ENSG00000136950) |
| [ENSG00000175854](http://www.ensembl.org/Homo_sapiens/Gene/Summary?db=core;g=ENSG00000175854) | [SWI5](http://www.ensembl.org/Homo_sapiens/Gene/Summary?db=core;g=ENSG00000175854) |
| [ENSG00000167106](http://www.ensembl.org/Homo_sapiens/Gene/Summary?db=core;g=ENSG00000167106) | [FAM102A](http://www.ensembl.org/Homo_sapiens/Gene/Summary?db=core;g=ENSG00000167106) |
| [ENSG00000119392](http://www.ensembl.org/Homo_sapiens/Gene/Summary?db=core;g=ENSG00000119392) | [GLE1](http://www.ensembl.org/Homo_sapiens/Gene/Summary?db=core;g=ENSG00000119392) |
| [ENSG00000160445](http://www.ensembl.org/Homo_sapiens/Gene/Summary?db=core;g=ENSG00000160445) | [ZER1](http://www.ensembl.org/Homo_sapiens/Gene/Summary?db=core;g=ENSG00000160445) |
| [ENSG00000175764](http://www.ensembl.org/Homo_sapiens/Gene/Summary?db=core;g=ENSG00000175764) | [TTLL11](http://www.ensembl.org/Homo_sapiens/Gene/Summary?db=core;g=ENSG00000175764) |
| [ENSG00000095319](http://www.ensembl.org/Homo_sapiens/Gene/Summary?db=core;g=ENSG00000095319) | [NUP188](http://www.ensembl.org/Homo_sapiens/Gene/Summary?db=core;g=ENSG00000095319) |
| [ENSG00000186130](http://www.ensembl.org/Homo_sapiens/Gene/Summary?db=core;g=ENSG00000186130) | [ZBTB6](http://www.ensembl.org/Homo_sapiens/Gene/Summary?db=core;g=ENSG00000186130) |
| [ENSG00000119487](http://www.ensembl.org/Homo_sapiens/Gene/Summary?db=core;g=ENSG00000119487) | [MAPKAP1](http://www.ensembl.org/Homo_sapiens/Gene/Summary?db=core;g=ENSG00000119487) |
| [ENSG00000107021](http://www.ensembl.org/Homo_sapiens/Gene/Summary?db=core;g=ENSG00000107021) | [TBC1D13](http://www.ensembl.org/Homo_sapiens/Gene/Summary?db=core;g=ENSG00000107021) |
| [ENSG00000167114](http://www.ensembl.org/Homo_sapiens/Gene/Summary?db=core;g=ENSG00000167114) | [SLC27A4](http://www.ensembl.org/Homo_sapiens/Gene/Summary?db=core;g=ENSG00000167114) |
| [ENSG00000119414](http://www.ensembl.org/Homo_sapiens/Gene/Summary?db=core;g=ENSG00000119414) | [PPP6C](http://www.ensembl.org/Homo_sapiens/Gene/Summary?db=core;g=ENSG00000119414) |
| [ENSG00000119408](http://www.ensembl.org/Homo_sapiens/Gene/Summary?db=core;g=ENSG00000119408) | [NEK6](http://www.ensembl.org/Homo_sapiens/Gene/Summary?db=core;g=ENSG00000119408) |
| [ENSG00000136908](http://www.ensembl.org/Homo_sapiens/Gene/Summary?db=core;g=ENSG00000136908) | [DPM2](http://www.ensembl.org/Homo_sapiens/Gene/Summary?db=core;g=ENSG00000136908) |
| [ENSG00000136918](http://www.ensembl.org/Homo_sapiens/Gene/Summary?db=core;g=ENSG00000136918) | [WDR38](http://www.ensembl.org/Homo_sapiens/Gene/Summary?db=core;g=ENSG00000136918) |
| [ENSG00000148337](http://www.ensembl.org/Homo_sapiens/Gene/Summary?db=core;g=ENSG00000148337) | [CIZ1](http://www.ensembl.org/Homo_sapiens/Gene/Summary?db=core;g=ENSG00000148337) |
| [ENSG00000136942](http://www.ensembl.org/Homo_sapiens/Gene/Summary?db=core;g=ENSG00000136942) | [RPL35](http://www.ensembl.org/Homo_sapiens/Gene/Summary?db=core;g=ENSG00000136942) |
| [ENSG00000171448](http://www.ensembl.org/Homo_sapiens/Gene/Summary?db=core;g=ENSG00000171448) | [ZBTB26](http://www.ensembl.org/Homo_sapiens/Gene/Summary?db=core;g=ENSG00000171448) |
| [ENSG00000106689](http://www.ensembl.org/Homo_sapiens/Gene/Summary?db=core;g=ENSG00000106689) | [LHX2](http://www.ensembl.org/Homo_sapiens/Gene/Summary?db=core;g=ENSG00000106689) |
| [ENSG00000171484](http://www.ensembl.org/Homo_sapiens/Gene/Summary?db=core;g=ENSG00000171484) | [OR1B1](http://www.ensembl.org/Homo_sapiens/Gene/Summary?db=core;g=ENSG00000171484) |
| [ENSG00000148346](http://www.ensembl.org/Homo_sapiens/Gene/Summary?db=core;g=ENSG00000148346) | [LCN2](http://www.ensembl.org/Homo_sapiens/Gene/Summary?db=core;g=ENSG00000148346) |
| [ENSG00000148334](http://www.ensembl.org/Homo_sapiens/Gene/Summary?db=core;g=ENSG00000148334) | [PTGES2](http://www.ensembl.org/Homo_sapiens/Gene/Summary?db=core;g=ENSG00000148334) |
| [ENSG00000106976](http://www.ensembl.org/Homo_sapiens/Gene/Summary?db=core;g=ENSG00000106976) | [DNM1](http://www.ensembl.org/Homo_sapiens/Gene/Summary?db=core;g=ENSG00000106976) |
| [ENSG00000196814](http://www.ensembl.org/Homo_sapiens/Gene/Summary?db=core;g=ENSG00000196814) | [MVB12B](http://www.ensembl.org/Homo_sapiens/Gene/Summary?db=core;g=ENSG00000196814) |
| [ENSG00000136895](http://www.ensembl.org/Homo_sapiens/Gene/Summary?db=core;g=ENSG00000136895) | [GARNL3](http://www.ensembl.org/Homo_sapiens/Gene/Summary?db=core;g=ENSG00000136895) |
| [ENSG00000136840](http://www.ensembl.org/Homo_sapiens/Gene/Summary?db=core;g=ENSG00000136840) | [ST6GALNAC4](http://www.ensembl.org/Homo_sapiens/Gene/Summary?db=core;g=ENSG00000136840) |
| [ENSG00000136944](http://www.ensembl.org/Homo_sapiens/Gene/Summary?db=core;g=ENSG00000136944) | [LMX1B](http://www.ensembl.org/Homo_sapiens/Gene/Summary?db=core;g=ENSG00000136944) |
| [ENSG00000165209](http://www.ensembl.org/Homo_sapiens/Gene/Summary?db=core;g=ENSG00000165209) | [STRBP](http://www.ensembl.org/Homo_sapiens/Gene/Summary?db=core;g=ENSG00000165209) |
| [ENSG00000169155](http://www.ensembl.org/Homo_sapiens/Gene/Summary?db=core;g=ENSG00000169155) | [ZBTB43](http://www.ensembl.org/Homo_sapiens/Gene/Summary?db=core;g=ENSG00000169155) |
| [ENSG00000160446](http://www.ensembl.org/Homo_sapiens/Gene/Summary?db=core;g=ENSG00000160446) | [ZDHHC12](http://www.ensembl.org/Homo_sapiens/Gene/Summary?db=core;g=ENSG00000160446) |
| [ENSG00000106852](http://www.ensembl.org/Homo_sapiens/Gene/Summary?db=core;g=ENSG00000106852) | [LHX6](http://www.ensembl.org/Homo_sapiens/Gene/Summary?db=core;g=ENSG00000106852) |
| [ENSG00000160447](http://www.ensembl.org/Homo_sapiens/Gene/Summary?db=core;g=ENSG00000160447) | [PKN3](http://www.ensembl.org/Homo_sapiens/Gene/Summary?db=core;g=ENSG00000160447) |
| [ENSG00000136830](http://www.ensembl.org/Homo_sapiens/Gene/Summary?db=core;g=ENSG00000136830) | [FAM129B](http://www.ensembl.org/Homo_sapiens/Gene/Summary?db=core;g=ENSG00000136830) |
| [ENSG00000177125](http://www.ensembl.org/Homo_sapiens/Gene/Summary?db=core;g=ENSG00000177125) | [ZBTB34](http://www.ensembl.org/Homo_sapiens/Gene/Summary?db=core;g=ENSG00000177125) |
| [ENSG00000136819](http://www.ensembl.org/Homo_sapiens/Gene/Summary?db=core;g=ENSG00000136819) | [C9orf78](http://www.ensembl.org/Homo_sapiens/Gene/Summary?db=core;g=ENSG00000136819) |
| [ENSG00000148175](http://www.ensembl.org/Homo_sapiens/Gene/Summary?db=core;g=ENSG00000148175) | [STOM](http://www.ensembl.org/Homo_sapiens/Gene/Summary?db=core;g=ENSG00000148175) |
| [ENSG00000136878](http://www.ensembl.org/Homo_sapiens/Gene/Summary?db=core;g=ENSG00000136878) | [USP20](http://www.ensembl.org/Homo_sapiens/Gene/Summary?db=core;g=ENSG00000136878) |
| [ENSG00000106992](http://www.ensembl.org/Homo_sapiens/Gene/Summary?db=core;g=ENSG00000106992) | [AK1](http://www.ensembl.org/Homo_sapiens/Gene/Summary?db=core;g=ENSG00000106992) |
| [ENSG00000160404](http://www.ensembl.org/Homo_sapiens/Gene/Summary?db=core;g=ENSG00000160404) | [TOR2A](http://www.ensembl.org/Homo_sapiens/Gene/Summary?db=core;g=ENSG00000160404) |
| [ENSG00000165219](http://www.ensembl.org/Homo_sapiens/Gene/Summary?db=core;g=ENSG00000165219) | [GAPVD1](http://www.ensembl.org/Homo_sapiens/Gene/Summary?db=core;g=ENSG00000165219) |
| [ENSG00000148343](http://www.ensembl.org/Homo_sapiens/Gene/Summary?db=core;g=ENSG00000148343) | [FAM73B](http://www.ensembl.org/Homo_sapiens/Gene/Summary?db=core;g=ENSG00000148343) |
| [ENSG00000136931](http://www.ensembl.org/Homo_sapiens/Gene/Summary?db=core;g=ENSG00000136931) | [NR5A1](http://www.ensembl.org/Homo_sapiens/Gene/Summary?db=core;g=ENSG00000136931) |
| [ENSG00000056586](http://www.ensembl.org/Homo_sapiens/Gene/Summary?db=core;g=ENSG00000056586) | [RC3H2](http://www.ensembl.org/Homo_sapiens/Gene/Summary?db=core;g=ENSG00000056586) |
| [ENSG00000095261](http://www.ensembl.org/Homo_sapiens/Gene/Summary?db=core;g=ENSG00000095261) | [PSMD5](http://www.ensembl.org/Homo_sapiens/Gene/Summary?db=core;g=ENSG00000095261) |
| [ENSG00000171159](http://www.ensembl.org/Homo_sapiens/Gene/Summary?db=core;g=ENSG00000171159) | [C9orf16](http://www.ensembl.org/Homo_sapiens/Gene/Summary?db=core;g=ENSG00000171159) |
| [ENSG00000136834](http://www.ensembl.org/Homo_sapiens/Gene/Summary?db=core;g=ENSG00000136834) | [OR1J1](http://www.ensembl.org/Homo_sapiens/Gene/Summary?db=core;g=ENSG00000136834) |
| [ENSG00000167094](http://www.ensembl.org/Homo_sapiens/Gene/Summary?db=core;g=ENSG00000167094) | [TTC16](http://www.ensembl.org/Homo_sapiens/Gene/Summary?db=core;g=ENSG00000167094) |
| [ENSG00000197233](http://www.ensembl.org/Homo_sapiens/Gene/Summary?db=core;g=ENSG00000197233) | [OR1J2](http://www.ensembl.org/Homo_sapiens/Gene/Summary?db=core;g=ENSG00000197233) |
| [ENSG00000148200](http://www.ensembl.org/Homo_sapiens/Gene/Summary?db=core;g=ENSG00000148200) | [NR6A1](http://www.ensembl.org/Homo_sapiens/Gene/Summary?db=core;g=ENSG00000148200) |
| [ENSG00000136854](http://www.ensembl.org/Homo_sapiens/Gene/Summary?db=core;g=ENSG00000136854) | [STXBP1](http://www.ensembl.org/Homo_sapiens/Gene/Summary?db=core;g=ENSG00000136854) |
| [ENSG00000239590](http://www.ensembl.org/Homo_sapiens/Gene/Summary?db=core;g=ENSG00000239590) | [OR1J4](http://www.ensembl.org/Homo_sapiens/Gene/Summary?db=core;g=ENSG00000239590) |
| [ENSG00000171505](http://www.ensembl.org/Homo_sapiens/Gene/Summary?db=core;g=ENSG00000171505) | [OR1N1](http://www.ensembl.org/Homo_sapiens/Gene/Summary?db=core;g=ENSG00000171505) |
| [ENSG00000171501](http://www.ensembl.org/Homo_sapiens/Gene/Summary?db=core;g=ENSG00000171501) | [OR1N2](http://www.ensembl.org/Homo_sapiens/Gene/Summary?db=core;g=ENSG00000171501) |
| [ENSG00000171496](http://www.ensembl.org/Homo_sapiens/Gene/Summary?db=core;g=ENSG00000171496) | [OR1L8](http://www.ensembl.org/Homo_sapiens/Gene/Summary?db=core;g=ENSG00000171496) |
| [ENSG00000280094](http://www.ensembl.org/Homo_sapiens/Gene/Summary?db=core;g=ENSG00000280094) | [OR1B1](http://www.ensembl.org/Homo_sapiens/Gene/Summary?db=core;g=ENSG00000280094) |
| [ENSG00000165202](http://www.ensembl.org/Homo_sapiens/Gene/Summary?db=core;g=ENSG00000165202) | [OR1Q1](http://www.ensembl.org/Homo_sapiens/Gene/Summary?db=core;g=ENSG00000165202) |
| [ENSG00000148180](http://www.ensembl.org/Homo_sapiens/Gene/Summary?db=core;g=ENSG00000148180) | [GSN](http://www.ensembl.org/Homo_sapiens/Gene/Summary?db=core;g=ENSG00000148180) |
| [ENSG00000167113](http://www.ensembl.org/Homo_sapiens/Gene/Summary?db=core;g=ENSG00000167113) | [COQ4](http://www.ensembl.org/Homo_sapiens/Gene/Summary?db=core;g=ENSG00000167113) |
| [ENSG00000173679](http://www.ensembl.org/Homo_sapiens/Gene/Summary?db=core;g=ENSG00000173679) | [OR1L1](http://www.ensembl.org/Homo_sapiens/Gene/Summary?db=core;g=ENSG00000173679) |
| [ENSG00000171481](http://www.ensembl.org/Homo_sapiens/Gene/Summary?db=core;g=ENSG00000171481) | [OR1L3](http://www.ensembl.org/Homo_sapiens/Gene/Summary?db=core;g=ENSG00000171481) |
| [ENSG00000119333](http://www.ensembl.org/Homo_sapiens/Gene/Summary?db=core;g=ENSG00000119333) | [WDR34](http://www.ensembl.org/Homo_sapiens/Gene/Summary?db=core;g=ENSG00000119333) |
| [ENSG00000185585](http://www.ensembl.org/Homo_sapiens/Gene/Summary?db=core;g=ENSG00000185585) | [OLFML2A](http://www.ensembl.org/Homo_sapiens/Gene/Summary?db=core;g=ENSG00000185585) |
| [ENSG00000187024](http://www.ensembl.org/Homo_sapiens/Gene/Summary?db=core;g=ENSG00000187024) | [PTRH1](http://www.ensembl.org/Homo_sapiens/Gene/Summary?db=core;g=ENSG00000187024) |
| [ENSG00000148204](http://www.ensembl.org/Homo_sapiens/Gene/Summary?db=core;g=ENSG00000148204) | [CRB2](http://www.ensembl.org/Homo_sapiens/Gene/Summary?db=core;g=ENSG00000148204) |
| [ENSG00000136939](http://www.ensembl.org/Homo_sapiens/Gene/Summary?db=core;g=ENSG00000136939) | [OR1L4](http://www.ensembl.org/Homo_sapiens/Gene/Summary?db=core;g=ENSG00000136939) |
| [ENSG00000171459](http://www.ensembl.org/Homo_sapiens/Gene/Summary?db=core;g=ENSG00000171459) | [OR1L6](http://www.ensembl.org/Homo_sapiens/Gene/Summary?db=core;g=ENSG00000171459) |
| [ENSG00000136877](http://www.ensembl.org/Homo_sapiens/Gene/Summary?db=core;g=ENSG00000136877) | [FPGS](http://www.ensembl.org/Homo_sapiens/Gene/Summary?db=core;g=ENSG00000136877) |
| [ENSG00000119403](http://www.ensembl.org/Homo_sapiens/Gene/Summary?db=core;g=ENSG00000119403) | [PHF19](http://www.ensembl.org/Homo_sapiens/Gene/Summary?db=core;g=ENSG00000119403) |
| [ENSG00000148215](http://www.ensembl.org/Homo_sapiens/Gene/Summary?db=core;g=ENSG00000148215) | [OR5C1](http://www.ensembl.org/Homo_sapiens/Gene/Summary?db=core;g=ENSG00000148215) |
| [ENSG00000119522](http://www.ensembl.org/Homo_sapiens/Gene/Summary?db=core;g=ENSG00000119522) | [DENND1A](http://www.ensembl.org/Homo_sapiens/Gene/Summary?db=core;g=ENSG00000119522) |
| [ENSG00000167130](http://www.ensembl.org/Homo_sapiens/Gene/Summary?db=core;g=ENSG00000167130) | [DOLPP1](http://www.ensembl.org/Homo_sapiens/Gene/Summary?db=core;g=ENSG00000167130) |
| [ENSG00000167112](http://www.ensembl.org/Homo_sapiens/Gene/Summary?db=core;g=ENSG00000167112) | [TRUB2](http://www.ensembl.org/Homo_sapiens/Gene/Summary?db=core;g=ENSG00000167112) |
| [ENSG00000167110](http://www.ensembl.org/Homo_sapiens/Gene/Summary?db=core;g=ENSG00000167110) | [GOLGA2](http://www.ensembl.org/Homo_sapiens/Gene/Summary?db=core;g=ENSG00000167110) |
| [ENSG00000136802](http://www.ensembl.org/Homo_sapiens/Gene/Summary?db=core;g=ENSG00000136802) | [LRRC8A](http://www.ensembl.org/Homo_sapiens/Gene/Summary?db=core;g=ENSG00000136802) |
| [ENSG00000171097](http://www.ensembl.org/Homo_sapiens/Gene/Summary?db=core;g=ENSG00000171097) | [CCBL1](http://www.ensembl.org/Homo_sapiens/Gene/Summary?db=core;g=ENSG00000171097) |
| [ENSG00000133135](http://www.ensembl.org/Homo_sapiens/Gene/Summary?db=core;g=ENSG00000133135) | [RNF128](http://www.ensembl.org/Homo_sapiens/Gene/Summary?db=core;g=ENSG00000133135) |
| [ENSG00000147224](http://www.ensembl.org/Homo_sapiens/Gene/Summary?db=core;g=ENSG00000147224) | [PRPS1](http://www.ensembl.org/Homo_sapiens/Gene/Summary?db=core;g=ENSG00000147224) |
| [ENSG00000188153](http://www.ensembl.org/Homo_sapiens/Gene/Summary?db=core;g=ENSG00000188153) | [COL4A5](http://www.ensembl.org/Homo_sapiens/Gene/Summary?db=core;g=ENSG00000188153) |
| [ENSG00000133131](http://www.ensembl.org/Homo_sapiens/Gene/Summary?db=core;g=ENSG00000133131) | [MORC4](http://www.ensembl.org/Homo_sapiens/Gene/Summary?db=core;g=ENSG00000133131) |
| [ENSG00000123572](http://www.ensembl.org/Homo_sapiens/Gene/Summary?db=core;g=ENSG00000123572) | [NRK](http://www.ensembl.org/Homo_sapiens/Gene/Summary?db=core;g=ENSG00000123572) |
| [ENSG00000197565](http://www.ensembl.org/Homo_sapiens/Gene/Summary?db=core;g=ENSG00000197565) | [COL4A6](http://www.ensembl.org/Homo_sapiens/Gene/Summary?db=core;g=ENSG00000197565) |
| [ENSG00000077264](http://www.ensembl.org/Homo_sapiens/Gene/Summary?db=core;g=ENSG00000077264) | [PAK3](http://www.ensembl.org/Homo_sapiens/Gene/Summary?db=core;g=ENSG00000077264) |
| [ENSG00000165376](http://www.ensembl.org/Homo_sapiens/Gene/Summary?db=core;g=ENSG00000165376) | [CLDN2](http://www.ensembl.org/Homo_sapiens/Gene/Summary?db=core;g=ENSG00000165376) |
| [ENSG00000133124](http://www.ensembl.org/Homo_sapiens/Gene/Summary?db=core;g=ENSG00000133124) | [IRS4](http://www.ensembl.org/Homo_sapiens/Gene/Summary?db=core;g=ENSG00000133124) |
| [ENSG00000101888](http://www.ensembl.org/Homo_sapiens/Gene/Summary?db=core;g=ENSG00000101888) | [NXT2](http://www.ensembl.org/Homo_sapiens/Gene/Summary?db=core;g=ENSG00000101888) |
| [ENSG00000147223](http://www.ensembl.org/Homo_sapiens/Gene/Summary?db=core;g=ENSG00000147223) | [RIPPLY1](http://www.ensembl.org/Homo_sapiens/Gene/Summary?db=core;g=ENSG00000147223) |
| [ENSG00000101842](http://www.ensembl.org/Homo_sapiens/Gene/Summary?db=core;g=ENSG00000101842) | [VSIG1](http://www.ensembl.org/Homo_sapiens/Gene/Summary?db=core;g=ENSG00000101842) |
| [ENSG00000182508](http://www.ensembl.org/Homo_sapiens/Gene/Summary?db=core;g=ENSG00000182508) | [LHFPL1](http://www.ensembl.org/Homo_sapiens/Gene/Summary?db=core;g=ENSG00000182508) |
| [ENSG00000101935](http://www.ensembl.org/Homo_sapiens/Gene/Summary?db=core;g=ENSG00000101935) | [AMMECR1](http://www.ensembl.org/Homo_sapiens/Gene/Summary?db=core;g=ENSG00000101935) |
| [ENSG00000101890](http://www.ensembl.org/Homo_sapiens/Gene/Summary?db=core;g=ENSG00000101890) | [GUCY2F](http://www.ensembl.org/Homo_sapiens/Gene/Summary?db=core;g=ENSG00000101890) |
| [ENSG00000089682](http://www.ensembl.org/Homo_sapiens/Gene/Summary?db=core;g=ENSG00000089682) | [RBM41](http://www.ensembl.org/Homo_sapiens/Gene/Summary?db=core;g=ENSG00000089682) |
| [ENSG00000068366](http://www.ensembl.org/Homo_sapiens/Gene/Summary?db=core;g=ENSG00000068366) | [ACSL4](http://www.ensembl.org/Homo_sapiens/Gene/Summary?db=core;g=ENSG00000068366) |
| [ENSG00000133138](http://www.ensembl.org/Homo_sapiens/Gene/Summary?db=core;g=ENSG00000133138) | [TBC1D8B](http://www.ensembl.org/Homo_sapiens/Gene/Summary?db=core;g=ENSG00000133138) |
| [ENSG00000101844](http://www.ensembl.org/Homo_sapiens/Gene/Summary?db=core;g=ENSG00000101844) | [ATG4A](http://www.ensembl.org/Homo_sapiens/Gene/Summary?db=core;g=ENSG00000101844) |
| [ENSG00000123561](http://www.ensembl.org/Homo_sapiens/Gene/Summary?db=core;g=ENSG00000123561) | [SERPINA7](http://www.ensembl.org/Homo_sapiens/Gene/Summary?db=core;g=ENSG00000123561) |
| [ENSG00000126016](http://www.ensembl.org/Homo_sapiens/Gene/Summary?db=core;g=ENSG00000126016) | [AMOT](http://www.ensembl.org/Homo_sapiens/Gene/Summary?db=core;g=ENSG00000126016) |
| [ENSG00000147231](http://www.ensembl.org/Homo_sapiens/Gene/Summary?db=core;g=ENSG00000147231) | [CXorf57](http://www.ensembl.org/Homo_sapiens/Gene/Summary?db=core;g=ENSG00000147231) |
| [ENSG00000268629](http://www.ensembl.org/Homo_sapiens/Gene/Summary?db=core;g=ENSG00000268629) | [TEX13A](http://www.ensembl.org/Homo_sapiens/Gene/Summary?db=core;g=ENSG00000268629) |
| [ENSG00000157514](http://www.ensembl.org/Homo_sapiens/Gene/Summary?db=core;g=ENSG00000157514) | [TSC22D3](http://www.ensembl.org/Homo_sapiens/Gene/Summary?db=core;g=ENSG00000157514) |
| [ENSG00000101938](http://www.ensembl.org/Homo_sapiens/Gene/Summary?db=core;g=ENSG00000101938) | [CHRDL1](http://www.ensembl.org/Homo_sapiens/Gene/Summary?db=core;g=ENSG00000101938) |
| [ENSG00000072315](http://www.ensembl.org/Homo_sapiens/Gene/Summary?db=core;g=ENSG00000072315) | [TRPC5](http://www.ensembl.org/Homo_sapiens/Gene/Summary?db=core;g=ENSG00000072315) |
| [ENSG00000077279](http://www.ensembl.org/Homo_sapiens/Gene/Summary?db=core;g=ENSG00000077279) | [DCX](http://www.ensembl.org/Homo_sapiens/Gene/Summary?db=core;g=ENSG00000077279) |
| [ENSG00000198088](http://www.ensembl.org/Homo_sapiens/Gene/Summary?db=core;g=ENSG00000198088) | [NUP62CL](http://www.ensembl.org/Homo_sapiens/Gene/Summary?db=core;g=ENSG00000198088) |
| [ENSG00000101901](http://www.ensembl.org/Homo_sapiens/Gene/Summary?db=core;g=ENSG00000101901) | [ALG13](http://www.ensembl.org/Homo_sapiens/Gene/Summary?db=core;g=ENSG00000101901) |
| [ENSG00000080561](http://www.ensembl.org/Homo_sapiens/Gene/Summary?db=core;g=ENSG00000080561) | [MID2](http://www.ensembl.org/Homo_sapiens/Gene/Summary?db=core;g=ENSG00000080561) |
| [ENSG00000101843](http://www.ensembl.org/Homo_sapiens/Gene/Summary?db=core;g=ENSG00000101843) | [PSMD10](http://www.ensembl.org/Homo_sapiens/Gene/Summary?db=core;g=ENSG00000101843) |
| [ENSG00000157600](http://www.ensembl.org/Homo_sapiens/Gene/Summary?db=core;g=ENSG00000157600) | [TMEM164](http://www.ensembl.org/Homo_sapiens/Gene/Summary?db=core;g=ENSG00000157600) |
| [ENSG00000080572](http://www.ensembl.org/Homo_sapiens/Gene/Summary?db=core;g=ENSG00000080572) | [PIH1D3](http://www.ensembl.org/Homo_sapiens/Gene/Summary?db=core;g=ENSG00000080572) |
| [ENSG00000077274](http://www.ensembl.org/Homo_sapiens/Gene/Summary?db=core;g=ENSG00000077274) | [CAPN6](http://www.ensembl.org/Homo_sapiens/Gene/Summary?db=core;g=ENSG00000077274) |
| [ENSG00000170935](http://www.ensembl.org/Homo_sapiens/Gene/Summary?db=core;g=ENSG00000170935) | [NCBP2L](http://www.ensembl.org/Homo_sapiens/Gene/Summary?db=core;g=ENSG00000170935) |
| [ENSG00000157502](http://www.ensembl.org/Homo_sapiens/Gene/Summary?db=core;g=ENSG00000157502) | [MUM1L1](http://www.ensembl.org/Homo_sapiens/Gene/Summary?db=core;g=ENSG00000157502) |
| [ENSG00000187823](http://www.ensembl.org/Homo_sapiens/Gene/Summary?db=core;g=ENSG00000187823) | [ZCCHC16](http://www.ensembl.org/Homo_sapiens/Gene/Summary?db=core;g=ENSG00000187823) |
| [ENSG00000176076](http://www.ensembl.org/Homo_sapiens/Gene/Summary?db=core;g=ENSG00000176076) | [KCNE5](http://www.ensembl.org/Homo_sapiens/Gene/Summary?db=core;g=ENSG00000176076) |
| [ENSG00000189108](http://www.ensembl.org/Homo_sapiens/Gene/Summary?db=core;g=ENSG00000189108) | [IL1RAPL2](http://www.ensembl.org/Homo_sapiens/Gene/Summary?db=core;g=ENSG00000189108) |
| [ENSG00000147234](http://www.ensembl.org/Homo_sapiens/Gene/Summary?db=core;g=ENSG00000147234) | [FRMPD3](http://www.ensembl.org/Homo_sapiens/Gene/Summary?db=core;g=ENSG00000147234) |
|  |  |
|  |  |
