# Supplementary figures and images for: HPV16 integration probably contributes to cervical oncogenesis through interrupting tumor suppressor genes and inducing chromosome instability
Source: J Exp Clin Cancer Res. 2016 Nov 25;35:180. doi: 10.1186/s13046-016-0454-4 (PMC5123399; doi:10.1186/s13046-016-0454-4)

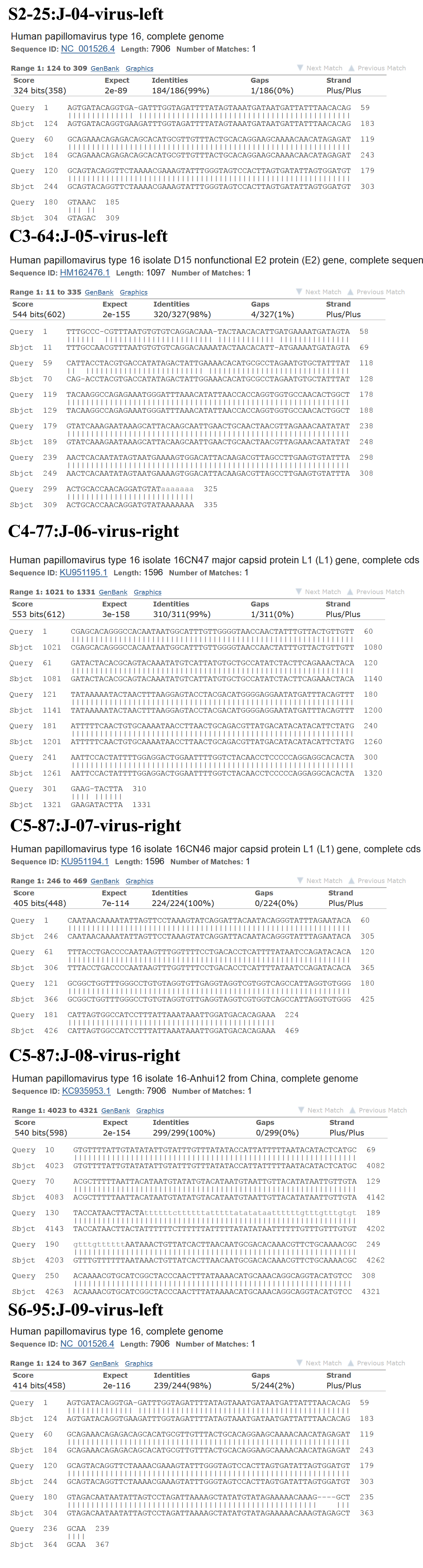

Supplement: Additional file 2: Figure S1. — The alignment between inserted viral elements (S2-25:J-04-virus-left, C3-64:J-05-virus-left, C4-77:J-06-virus-right, C5-87:J-07-virus-right, C5-87:J-08-virus-right, S6-95:J-09-virus-left) and most closely related HPV16 sequences. The “Query” indicates the viral sequences while the “sbjct” indicates the HPV16 sequences acquired from the NCBI database. (TIF 3984 kb) [file 13046_2016_454_MOESM2_ESM.tif]
